# Transforming a Historical Chemical Synthetic Route for Vanillin Starting from Renewable Eugenol to a Cell‐Free Bi‐Enzymatic Cascade

**DOI:** 10.1002/cssc.202500387

**Published:** 2025-04-16

**Authors:** Elisa Lanfranchi, Valerio Ferrario, Somayyeh Gandomkar, Stefan E. Payer, Erna Zukic, Haris Rudalija, Alexandra Musi, Ines Gaberscek, Yuliya Orel, Doreen Schachtschabel, Christian Willrodt, Michael Breuer, Wolfgang Kroutil

**Affiliations:** ^1^ Austrian Center of Industrial Biotechnology (ACIB GmbH) c/o Department of Chemistry University of Graz Heinrichstraße 28 8010 Graz Austria; ^2^ Group Research BASF SE Carl-Bosch-Strasse 38 67056 Ludwigshafen am Rhein Germany; ^3^ Department of Chemistry University of Graz Heinrichstraße 28 8010 Graz Austria; ^4^ BioTechMed Graz 8010 Graz Austria; ^5^ Field of Excellence BioHealth University of Graz 8010 Graz Austria

**Keywords:** vanillin, eugenol, biocatalysis, cascade, C=C bond cleavage •

## Abstract

Vanillin is one of the most important aroma compounds, naturally occurring in vanilla pods. Many routes to access natural vanillin from various renewables have been investigated, including a natural five‐step microbial transformation of eugenol to vanillin. Readily available eugenol was also the starting material for a chemical two‐step sequence to vanillin employed in the 19^th^ century. Here we show that a two‐step sequence can also be realized using biocatalysts only and run it in one‐pot simultaneously. This was achieved by isomerizing the C=C double bond of eugenol by oxidation to coniferyl alcohol followed by oxidative C=C cleavage catalyzed by newly identified enzymes. Thus, two oxidative steps catalyzed by two different biocatalysts ‐ one containing flavin and the other a non‐heme iron(II) cofactor ‐ were successfully run simultaneously just requiring molecular oxygen as oxidant for each step. Using natural eugenol sources, e. g. clove oil, vanillin was obtained with 91 % product formation. This study shows that natural pathways like the microbial transformation of eugenol to vanillin involving five steps can be shortened, hereto just two simultaneous steps, by exploiting and combining the repertoire of promiscuous enzymatic activities present in different organisms leading to new‐to‐nature cascades.

## Graphical abstract

An artificial enzymatic cascade to access natural vanillin from eugenol/clove oil was developed by revisiting the very first vanillin synthetic process from 19^th^ century. The biosynthetic route consists of just two biocatalysts used as cell‐free preparation and molecular oxygen as oxidizing agent, outperforming also the natural microbial pathway which requires 5 steps.

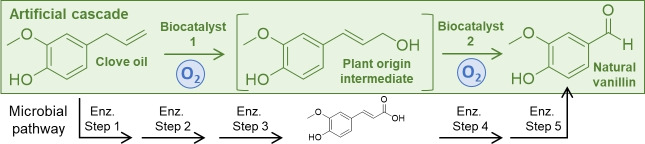

## Introduction

**Scheme 1:**
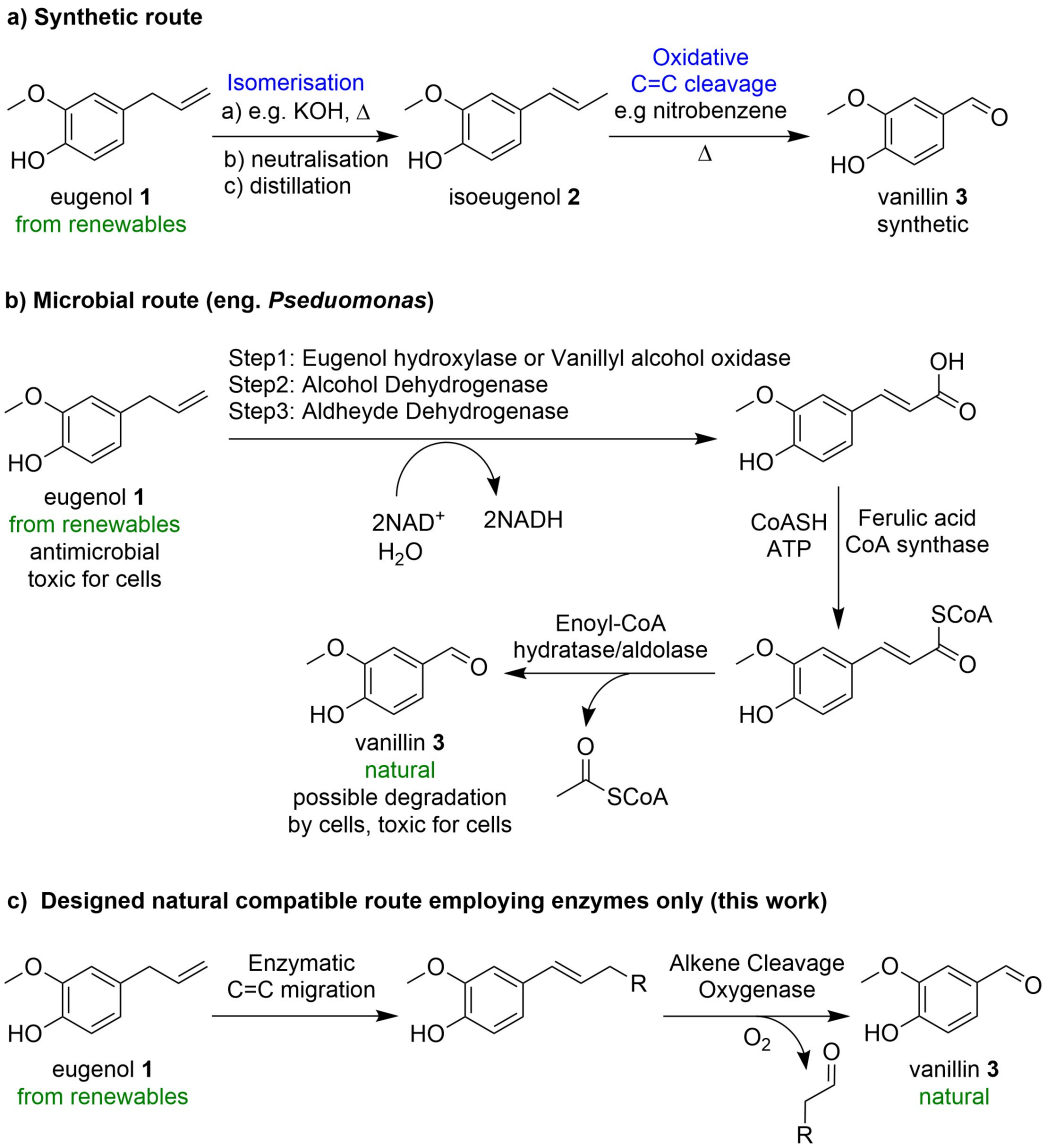
Approaches for the synthesis of vanillin from eugenol.

Vanillin **3** is used in a plethora of products e. g. in food and beverages, cosmetics and pharmaceuticals.[^1^, ^2^, ^3^] In 1874, more than 150 years ago, the very first chemical synthesis of vanillin **3** was patented by Ferdinand Tiemann and Wilhelm Haarmann starting from coniferin.[^4^, ^5^, ^6^, ^7^] Due to technical difficulties to get access to sufficient amounts of coniferin,^[7]^ clove oil/eugenol **1** was shortly after identified as an alternative starting material triggering the historical two‐step process to vanillin **3** involving an isomerization step under alkaline conditions followed by an oxidative C=C cleaving step using for instance permanganate or nitrobenzene as oxidant (Scheme 1a).[^8^, ^9^, ^10^] This route was exploited for the production of vanillin until – at least – the 1920s.^[11]^ Although the starting material at that time was natural, thus from renewables, the product would nowadays not been considered as natural in Europe according to European law due to the chemical steps involved; thus, the vanillin obtained is described as synthetic.^[12]^ Synthetic vanillin is today mostly produced from guaiacol, ferulic acid^[13]^ or lignin.^[14]^ EC regulation No 1334/2008 defines that natural vanillin may be obtained from the vanilla orchid (*vanilla planifolia*)^[15]^ or by taking a precursor from natural sources and transform it into vanillin by biotechnological means, like fermentation or enzymatic synthesis.[^12^, ^16^, ^17^, ^18^, ^19^, ^20^]

Using fermentation, various starting materials such as ferulic acid,[^21^, ^22^, ^23^] described in various commercial process,[^24^, ^25^, ^26^, ^27^, ^28^] but also sugars or L‐tyrosine have been considered for the production of natural vanillin.[^29^, ^30^, ^31^] Significant efforts have focused on using eugenol **1** as it is abundantly present in clove oil; for instance, wild type microorganisms such as *Pseudomonas sp*. HR199 have been described to transform eugenol **1** to vanillin **3** via five metabolic steps including a three step oxidation sequence to ferulic acid followed by activation to the corresponding SCoA‐derivative, hydration and retro‐Aldol reaction (Scheme 1b).^[32]^ Recently, eugenol **1** (50 mM) was transformed to vanillin **3** with 21 % conversion using the fungus *Daldinia* sp.^[33]^ Very recently, an engineered microbial pathway with one step less, thus four steps, was reported.^[34]^ Vanillin **3** has been identified as toxic for microbial cells and it is further metabolised via oxidation (to vanillic acid) and demethylation (to protocatechuic acid).[^21^, ^35^] Consequently, strains were engineered to minimize side reactions by e. g. disrupting the vanillin dehydrogenase gene to achieve a molar yield of 44.6 % reaching 2.9 mM of vanillin **3**.^[36]^ Alternatively, part of the *Pseudomonas* pathway was introduced in *E. coli* together with the vanillyl alcohol oxidase (VAO) from *Penicillum simplicissimum*. In this way, 0.3 g/L of vanillin **3** (equals 2 mM) and 0.1 g/L of vanillyl alcohol were obtained from a procedure involving two microbial strains, whereas longer incubation time led to a complete loss of the vanillin **3**.^[37]^

In comparison, single step enzymatic transformations to vanillin **3** from divers starting materials have been reported like using vanillic acid^[38]^ or vanillyl alcohol employing e. g. in the latter case the eugenol oxidase from *Rhodococcus jostii*,[^16^, ^17^, ^18^, ^19^, ^20^, ^39^] or using isoeugenol **2** employing for instance engineered variants of NOV1 from *Novosphingobium aromaticivorans* DSM 12444.[^40^, ^41^] Furthermore, ferulic acid was transformed via two‐steps to vanillin via decarboxylation and alkene cleavage,[^42^, ^43^, ^44^, ^45^, ^46^, ^47^] or most recently just via C=C cleavage.^[48]^ In a recent work, the enzyme NOV1 S283F was combined with the engineered PROGO^[49]^ to obtain vanillin from 4‐*n*‐propylguaiacol – a component of lignin oil – with 90 % yield.^[50]^ Cascades, as used in the last two examples, have gained in general increasing interest during the last decade as an option to combine several steps simultaneously or sequentially in one pot (see recent examples[^51^, ^52^, ^53^, ^54^, ^55^, ^56^] and selected reviews[^57^, ^58^, ^59^, ^60^, ^61^, ^62^, ^63^, ^64^, ^65^, ^66^, ^67^, ^68^, ^69^]).

Although the original synthetic route from the 19^th^ century for transforming eugenol **1** to vanillin **3** just requires two chemical steps (Scheme 1a), we wondered whether a related sequences just using enzymes is feasible (Scheme 1c).

## Results and Discussion

**Scheme 2:**
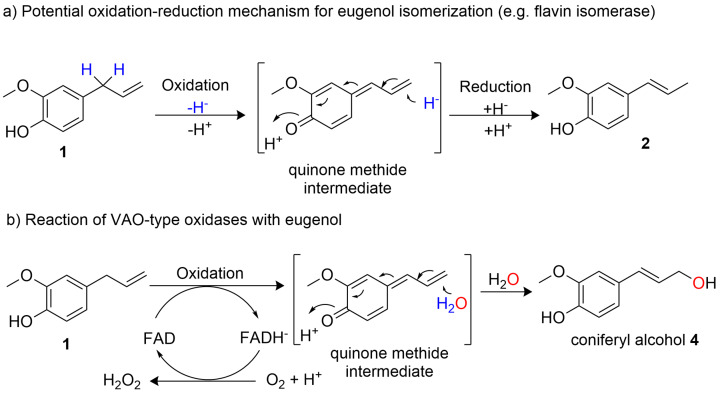
Comparison of envisioned reaction mechanism for the eugenol isomerization (a) and the reaction catalyzed by VAO‐type oxidases (b). **a)** Envisioned mechanism to isomerize eugenol **1** to isoeugenol **2**. Literature known isomerases exploit e. g. the flavin cofactor for abstracting a hydride from the substrate and “repositioning” it on another carbon like the linoleic isomerase from *Propionibacterium acnes*.[^70^, ^71^] Isoeugenol and eugenol synthases (IGS and EGS respectively) are NADPH dependent enzymes, which catalyze the formation of either isoeugenol or eugenol from coniferyl acetate also via quinone methide intermediate. By mutating specific residues of the active site, promiscuous variants have been generated, thereby a mixture of two isomers was obtained as product (eg. *Cb*IGS1‐V84F).[^72^, ^73^] We hypothesized that these variants may catalyze the isomerization with NADP^+^ for hydride abstraction and then returning the hydride in an alternative position. **B** VAO‐type oxidases catalyze the formation of coniferyl alcohol from eugenol via quinone methide intermediate, while the flavin cofactor is regenerated in the second half reaction by transferring electrons to molecular oxygen.^[74]^

Translating the two‐step 19^th^ century synthetic route transforming eugenol **1** to vanillin **3** (Scheme 1a) into an enzymatic cascade would require a C=C‐bond isomerase and an alkene cleaving enzyme. Unfortunately, a suitable isomerase catalyzing the migration of the double bond converting eugenol **1** to isoeugenol **2** is not known. Considering isomerization mechanisms, one notices, that an acid‐base mechanism as in the 19^th^ century route may not be applicable at ambient conditions, as a deprotonation in the benzylic position would lead to a benzylic anion not compatible with the phenolic OH. Consequently, a positive charge at the benzylic position may be favored, inspiring the following plausible mechanism involving an oxidation step (hydride abstraction) to form a *p*‐quinone methide intermediate, followed by a regioselective hydride back‐addition (Scheme 2a).

Consequently, enzymes described with a comparable redox mechanism for other substrates were chosen from literature such as the fatty acid isomerase from *Cutibacterium acnes* (PDB: 2BAB).[^70^, ^71^] Additionally, variants of the unspecific eugenol‐isoeugenol synthases (e. g. *Cb*IGS1 V84F)[^72^, ^73^] were tested for promiscuous activity to isomerize eugenol **1** to isoeugenol **2**. However, no suitable activity was found either because of unsuccessful recombinant expression or due to lack of isomerization activity (Figures S3A and S3B). Also, the retro‐synthesis tool Retrobiocat did not suggests any alternative enzymatic pathway (Figures S1 and S2).[^75^, ^76^, ^77^, ^78^] The game changer was when we noticed a kind of analogy between the suggested required mechanism for positional isomerization and the reaction catalyzed by vanillyl alcohol oxidases (VAO‐type oxidases), which catalyze beside various reactions also the transformation of eugenol to coniferyl alcohol via formation of a *p*‐quinone methide intermediate species (Scheme 2b).[^74^, ^79^] We imagined that the thus formed coniferyl alcohol **4** may subsequently be transformed to vanillin **3** by oxidative cleavage of the C=C bond, raising the question of whether this transformation of **4** is feasible in an efficient manner as it has interestingly not been described at all until very recently.^[48]^

Considering the first step, the flavin dependent eugenol oxidase from *Rhodococcus jostii* (Uniprot: Q0SBK1; *Rj*EUGO)[^80^, ^81^] is a VAO‐type oxidase which catalyzes the desired oxidation of eugenol to coniferyl alcohol with high efficiency in a wide pH window with a preference of slightly basic conditions.^[80]^ Consequently, we selected *Rj*EUGO as biocatalyst for the first step of our envisioned cascade. Without any further optimization, 50 mM of eugenol **1** (25 mg, 0.149 mmol) was transformed with >99 % conversion to coniferyl alcohol **4** (Figure S27); A minor amount (6 %) of the product **4** was oxidized further to coniferyl aldehyde **5** by the endogenous enzymes from *E. coli*.[^82^, ^83^] Finally, after isolation and purification by column chromatography, coniferyl alcohol **4** was obtained in 90 % isolated yield.

Unlike the first step, the subsequent enzymatic C=C cleavage of coniferyl alcohol **4** has not been investigated except one single recent report.^[48]^ Bacterial non‐heme iron oxygenases have been described to catalyze e. g. the C=C cleavage of isoeugenol **2** to give vanillin **3** and acetaldehyde (EC 1.13.11.88).[^84^, ^85^] Although representatives of this class were named isoeugenol monooxygenases, we will refer to them as isoeugenol cleavage oxygenases (IECOs) or – more generally – as alkene cleavage oxygenases (ACOs). Since coniferyl alcohol **4** differentiates from isoeugenol **2** only by a hydroxy group at the allylic carbon, one may hypothesize that these IECOs may also catalyze the target reaction. Consequently, three IECOs were selected originating from *Pseudomonas sp*. (GenBank: AXB59146; *Ps*IECO),^[86]^ *Pseudomonas putida* (GenBank: BAF62888; *Pp*IECO)^[87]^ and *Pseudomonas nitroreducens* (GenBank: ACP17973; *Pn*IECO).[^88^, ^89^, ^90^]

Among these, *Pn*IECO was successfully recombinantly produced in *E. coli* (Figure S3C). To our delight, *Pn*IECO cleaved coniferyl alcohol **4** to yield 0.85 mM vanillin **3** (17 % conversion) within 16 hours (Table S2). To preserve the operability of *Pn*IECO, iron(II) salts (1 mM) antioxidant (ascorbate, 1 mM) as well as bovine liver catalase were added, affording 3.6±0.6 mM vanillin **3**, equal to 73±5% conversion (Figure S4). The beneficial effect of catalase, to protect proteins from reactive oxygen species, has been shown also for other non‐heme enzymes, Cso2 from *Caulobacter segnis* for example.^[91]^

**Scheme 3:**
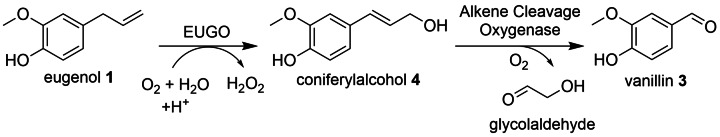
Two‐step enzymatic cascade to transform eugenol **1** to vanillin **3**.

In a next step, the viability of the envisioned cascade (Scheme 3) was tested combining the two selected enzymes – *Rj*EUGO and *Pn*IECO ‐ in a one pot concurrent cascade, thus adding all enzymes and reagents at the onset of the reaction, with 5 mM of starting material eugenol **1**. Running the two molecular oxygen dependent oxidation steps (*Rj*EUGO‐*Pn*IECO) simultaneously led indeed to successful vanillin **3** formation (Figure S22) with 99 % conversion of eugenol **1** within 24 hours. Optimizing the amount of the biocatalysts, led finally to 60 mol‐% vanillin **3** in the product mixture at 97 % substrate conversion (Table 1). Note that using freeze‐dried or freshly prepared (=not freeze‐dried) cell‐free biocatalyst preparations resulted in similar results (Table 1 and Table S3 respectively).

**Table 1:** One pot biocascade to transform eugenol to vanillin using *Rj*EUGO and *Pn*IECO. Variation of enzyme loading.^[a]^

*Rj*EUGO^[b]^ [mg/mL]	*Pn*IECO^[c]^ [mg/mL]	pH	Product distribution [%]^[d]^				Conv. of eugenol **1** ^[d]^ [%]
			Eugenol 1	4	**5**	Vanillin 3	
1	4	8	1	52	≤1	46	99
		9	2	43	≤1	54	98
2	4	8	2	48	2	48	98
		9	3	44	1	52	97
1	6	8	2	47	1	50	98
		9	3	36	≤1	60	97
2	6	8	3	50	3	45	97
		9	3	46	1	50	97
5	20	8	6	79	5	10	94
		9	7	73	5	15	93

[a] Reaction conditions (0.5 mL in 1.5 mL glass vials): *Rj*EUGO (freeze‐dried CFE), *Pn*IECO (freeze‐dried CFE) and bovine liver catalase (10 mg/mL) were mixed in the reaction buffer. The reaction was initiated by the addition of eugenol (25 μL from 100 mM stock in ethanol, final conc. 5 mM). Incubation: 30 °C and 120 rpm, 16 h. The vials were shaken in horizontal position. [b] 1 mg of *Rj*EUGO cell free extract corresponds to 382 mU. The activity of the biocatalyst preparation was measured spectrophotometrically with a reported standard enzymatic assay (vanillyl alcohol 0.5 mM; glycine‐NaOH 50 mM pH 9.0; 25 °C; 349 nm).^[39]^ [c] 1 mg of *Pn*IECO cell free extract corresponds to 2.7 mU. The activity of the biocatalyst preparation was measured spectrophotometrically with a standard enzymatic assay (coniferyl alcohol 0.5 mM; glycine‐NaOH pH 9.0; 25 °C; 349 nm). [d] Determined by HPLC‐UV using calibration curves (see SI, figures S30–S34).

**Figure 1:**
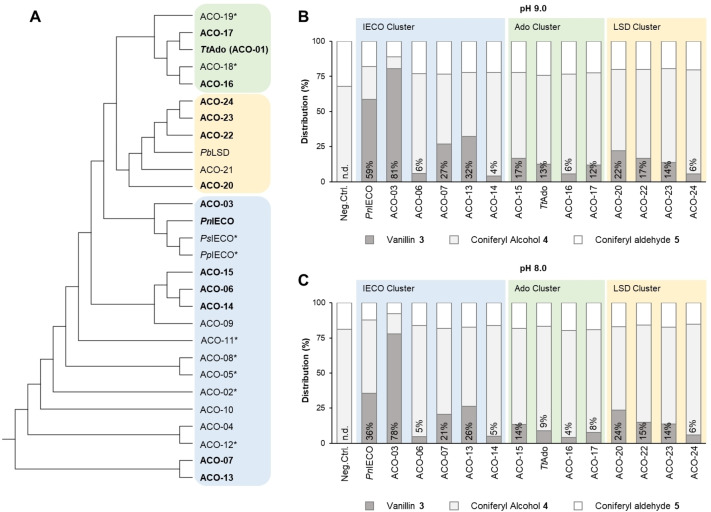
Screening of new cleavage oxygenases. **A**: Phylogenetic tree of selected putative cleavage oxygenases (cladogram representation). The three colored boxes highlight the different protein clusters. Active hits are in bold, while the not soluble expressed proteins are marked with a star. **B**: One pot biocascade with *Rj*EUGO and different ACOs in glycine‐NaOH pH 9.0. **C**: One pot biocascade with *Rj*EUGO and different ACOs in Tris‐HCl pH 8.0. Reaction conditions (**B** and **C**): *Rj*EUGO (freeze‐dried CFE, 1 mg/mL, 382 mU/mL), ACO‐XX (freeze‐dried CFE, 6 mg/mL), bovine liver catalase (10 mg/mL, ≥10 kU/mL), sodium ascorbate (1 mM), FeSO4*7H2O (1 mM), 5 % v/v ethanol and eugenol (5 mM) were mixed in glycine‐NaOH (50 mM pH 9.0; figure B) or Tris‐HCl (50 mM pH 8.0; figure C) to a final volume of 0.5 mL (1.5 mL glass vials). Incubation: 30 °C and 120 rpm for 24 hours. Product distribution was determined by HPLC‐UV based on calibration (see SI, figures S30‐S34). The negative control was done with ACO‐08 CFE (not soluble expressed variant, 0 mU/mg). The uniprot number and the amino acid sequence of each ACO are reported in the supplementary information.

Inspired by the initial positive results as well as the need for a better C=C cleaving enzyme, novel alkene cleavage oxygenases (ACOs) were needed to catalyze the efficient formation of vanillin **3** from coniferyl alcohol **4**. Considering the three IECOs as well as the characterized lignostillbene dioxygenase from *Pseudomonas brassicaceraum* (*Pb*LSD, PDB: 5 V2D)^[92]^ and the aromatic dioxygenase from *Thermothelomyces thermophilus* (*Tt*Ado, CO‐01; GenBank ID: XP_003665585),^[45]^ an enrichment procedure was performed where sequences within a given E‐value^[93]^ threshold were selected from public databases (e. g. UniProt)^[94]^ generating a final dataset of more than 15000 protein sequences. The latter was hierarchal clustered in order to generate an phylogenetic tree.^[95]^ 23 putative ACOs were arbitrary selected from three clusters each containing one of the initial three seed sequences (*Pn*IECO, *Pb*LSD and *Tt*ADO) to cover a broad sequence space of the clusters. These proteins were screened for enzymatic cleavage of coniferyl alcohol **4** by spectrophotometric assay, resulting in 13 positive hits, namely 12 uncharacterized ACOs (ACO 03; 06; 07; 13; 14; 15; 16; 17; 20; 22; 23 and 24) as well as the thermostable *Tt*Ado (Table S4). Results showed that the desired coniferyl alcohol cleaving activity was not cluster specific, as positive variants were found in selected branches of the phylogenetic tree covering a broad sequence space (Pairwise sequence identity≥33 %; Figure S9). Nevertheless, most active variants belonged to the *Pn*IECO cluster, while the specific activity in the other clusters was rather low. Subsequently, the active enzymes (ACO‐03, −06, −07, −13, −16, −17, −20, −22, −23, −24 and *Tt*Ado) were tested in combination with *Rj*EUGO in the one‐pot concurrent cascade (Figure 1B and 1 C). Bioreactions were in line with the activity screening, thus ‐ starting from 5 mM eugenol **1** ‐ vanillin **3** was formed with any selected ACO, even those with poor activity, whereby coniferyl alcohol **4** was better accepted by members of the IECO cluster. Best results were obtained with ACO‐03 giving 88 mol‐% vanillin **3** at >99 % conversion of **1** at pH 9.0 (Table 2, Entry 2). The formation of vanillin **3** upon enzymatic cleavage of coniferyl alcohol was also confirmed by NMR of the isolated and purified compound (Figures S36 and S37).

**Table 2:** One‐pot bi‐enzymatic biocascade to (natural) vanillin **3** from various sources of eugenol **1**.^[a]^

Entry^]^	Eugenol 1 source	Purity of eugenol 1	pH	Product distribution [%]^[b]^				Conv. of eugenol 1 [%]^[b]^
				Eugenol 1	4	5	Vanillin 3	
1	Synthetic eugenol	99 %	8.0	n.d.	8	9	83	>99
2			9.0	n.d	8	4	88	>99
3	Natural eugenol	Food grade ≥98 %	8.0	n.d.	6	10	84	>99
4			9.0	n.d.	6	4	90	>99
5	Clove oil	Food grade ≥76 %^[c]^	8.0	n.d.	9	12	79	>99
6			9.0	n.d.	4	5	91	>99

[a] Reaction condition: *Rj*EUGO (freeze‐dried CFE, 382 mU/mL), ACO‐03 (freeze‐dried CFE; 60 mU/mL), bovine liver catalase (≥10 kU/mL) were mixed in Tris‐HCl buffer (50 mM, pH 8.0) or Glycine‐NaOH buffer (50 mM, pH 9.0) containing FeSO_4_ (1 mM), sodium ascorbate (1 mM), eugenol **1** (5 mM) and 5 % v/v ethanol. The results are an average of two replicates. [b] Determined by HPLC‐UV using calibration curves (see SI, figures S30‐S34). N.d.: not detected. [c] Determined by GC‐MS (Figure S21): 6 % were eugenyl acetate, the remaining terpenes. The amount of clove oil was adjusted to have 5 mM eugenol in the reaction mixture.

Up to this point the biocatalytically produced vanillin **3** has still to be considered as “synthetic”, because synthetic substrate was used. To bio‐synthesize natural vanillin **3**, the cascade was subsequently run by using natural eugenol **1** as well as clove oil (Table 2, entries 3–6). Gratifyingly, biobased eugenol was completely converted, even when the more complex starting material ‐ clove oil ‐ was used, reaching up to 91 % of natural vanillin **3** formation. Even the eugenyl acetate present in clove oil was consumed after spontaneous hydrolysis.

Finally, the cascade was tested also at 50 mM substrate concentration. For these experiments the C=C cleaving enzyme ACO‐03 was modified by exchanging a cysteine (C26) located on the surface to an asparagine, as this cysteine was expected to be prone to oxidation and higher oxygen input is required at higher substrate loading.^[96]^ The obtained variant ACO‐03 C26 N turned to be comparable active as the wild type, also in the one pot cascade with clove oil (Figure S12). Nevertheless, at this elevated substrate concentration, the overall reaction turned out to be limited by the stability of ACO‐03 (Figure S11C and S12 C) as well as its reduced activity at increased concentrations of eugenol **1** and vanillin **3** (Figure S10 and S11D). These challenges were best addressed by performing the cascade in a one‐pot sequential fashion. In a first experiment at increased concentration of antioxidant as well as hourly feeding of fresh iron(II), eugenol **1** (50 mM) was completely converted to coniferyl alcohol **4** and up to 30 % vanillin **3** was formed (Table S6). An additional challenge was the supply of molecular oxygen for two reaction steps at this elevated substrate concentration. The low solubility of oxygen in water is a notorious challenge for the industrial application of oxidative enzymatic reactions.[^97^, ^98^] Especially the alkene cleaving enzyme seemed to have low affinity to molecular oxygen compared to *Rj*EUGO, which went easily to completion under the conditions established (table 3, entry 1). To address the challenge of molecular oxygen for the alkene cleaving enzyme, molecular oxygen for the transformation of **4** was supplied by continuously bubbling into the reaction mixture (Table 3, entries 3 and 4). The results showed that ACO‐03 C26 N benefited from steady oxygen supply, hence vanillin **3** formation reached 64 % and 50 % on a 3 mL and 10 mL scale, respectively. Considering the results of the two‐step transformation, 60 % conversion of eugenol 1 at 50 mM concentration (25 mg eugenol) to **3** was achieved, which corresponds to 30 mM (4.6 g/L) of vanillin **3**. This corresponds to a 10‐fold increase of product concentration in comparison to fermentative processes with eugenol as starting material where about 3 mM vanillin **1** was obtained,[^36^, ^37^] a 3‐fold improvement using the fungus *Daldinia* sp.^[33]^ and a comparable result to highly engineered strains.^[34]^

**Table 3:** Two step biosynthesis of vanillin from 50 mM eugenol.^[a]^

Entry	Step	Enzyme	Oxygen supply	Flask	Scale	Conv. ^[e]^	Product^[e]^	
					(mL)	[%]	[mM]	[%]
1	Comp. **1**→Comp. **4**	*Rj*EUGO^[a]^	stirring and open vessel	round bottom	3	>99	47	94
2	Comp. **4**→Comp. **3**	ACO‐03 C26N^[b]^	shaking^[c]^	Erlenmeyer	10	45	18	36
3	Comp. **4**→Comp. **3**	ACO‐03 C26N^[b]^	bubbling and stirring^[d]^	round bottom	3	71	32	64
4	Comp. **4**→Comp. **3**	ACO‐03 C26N^[b]^	bubbling and stirring^[d]^	Erlenmeyer	10	62	25	50

[a] Reaction condition: *Rj*EUGO (freeze‐dried CFE 10 mg/mL; 3.8 U/mL U), bovine liver catalase (10 mg/mL; ≥10 kU/mL) were mixed in Tris‐HCl buffer (50 mM, pH 8.0) synthetic eugenol (Fc 50 mM) and 5 % v/v DMSO. The reaction was stirred at room temperature for 18 hours. [b] Reaction condition: ACO‐03 C26N (freeze‐dried CFE 15 mg/mL; 150 mU/mL), bovine liver catalase (10 mg/mL; ≥10 kU/mL) were mixed in Tris‐HCl buffer (50 mM, pH 8.0) containing FeSO_4_ (Fc 1 mM), sodium ascorbate (Fc 1 mM), DTT (Fc 1 mM), 5 % v/v DMSO and coniferyl alcohol (Fc 50 mM). During incubation fresh iron salt was added every two hours (Fc 1 mM). [c] The reaction was shaken at 120 rpm for 17 hours at 30 °C and 120 rpm. [d] The reaction was incubated for 17 hours at 30 °C. Oxygen was bubbled for the first 7 hours. [e] Determined by HPLC‐UV based on calibration (see SI, figures S30‐S34).

## Conclusions

In summary, we describe the first cell‐free biocatalytic route to produce natural vanillin from eugenol requiring only two enzymes and molecular oxygen as the only oxidant. By this approach, a historical chemical route was mimicked by a biocatalytic cascade providing access to natural vanillin and setting the basis for a more techno economically and more efficient industrial production of vanillin from eugenol. Running the two oxidation steps in a concurrent fashion at 5 mM substrate concentration allowed to reach complete conversion and up to 91 % of vanillin formation. This was achieved by mining the carotenoid oxygenase family, identifying 14 enzymes catalyzing the cleavage of coniferyl alcohol, which were combined in a one‐pot concurrent reaction with *Rj*EUGO for the synthesis of vanillin. Best conversion was obtained with ACO‐03 from *Sphingomonadales bacterium*. The low sequence identity between the active variants suggests that many more are accessible in the same or different clusters. It was demonstrated that both eugenol and crude clove oil can be equally used as starting material to access vanillin. Though elevated substrate loading is desired, the C=C cleaving enzyme needs to be further improved for higher stability e. g. for 50 mM substrate concentration. Nevertheless, the concentrations of vanillin reached when using 50 mM substrate exceeded all biotechnological approaches starting from eugenol by 1.5–10 folds. Probably most important, the study showed that a designed new‐to‐nature cascade can outperform the biosynthetic pathways present in microorganisms as the number of steps can be reduced from five in the microbial pathway to just two. This shows the potential of artificial cascades combining enzymes from different organisms to achieve an even shorter synthetic route. This approach will inspire further development to cut‐short synthetic routes and stimulate to revisit also natural biosynthetic pathways to identify possible even shorter routes.

## Supporting Information

The authors have cited additional references within the Supporting Information.[^99^, ^100^, ^101^, ^102^, ^103^, ^104^, ^105^]

## Conflict of Interests

Elisa Lanfranchi, Valerio Ferrario, Christian Willrodt, Michael Breuer, Wolfgang Kroutil are co‐authors of a patent related to the cascade presented here

1

## Supporting information

As a service to our authors and readers, this journal provides supporting information supplied by the authors. Such materials are peer reviewed and may be re‐organized for online delivery, but are not copy‐edited or typeset. Technical support issues arising from supporting information (other than missing files) should be addressed to the authors.

Supporting Information

## Data Availability

The data that support the findings of this study are available in the supplementary material of this article.
