# Supplementary material for: Transforming a Historical Chemical Synthetic Route for Vanillin Starting from Renewable Eugenol to a Cell‐Free Bi‐Enzymatic Cascade
Source: ChemSusChem. 2025 Apr 16;18(11):e202500387. doi: 10.1002/cssc.202500387 (PMC12131681; doi:10.1002/cssc.202500387)
Supplement: Supplementary file 1 — Supporting Information [file CSSC-18-e202500387-s001.pdf]

ChemSusChem

Supporting Information

Transforming a Historical Chemical Synthetic Route for Vanillin Starting from Renewable Eugenol to a Cell-Free Bi-Enzymatic Cascade

Elisa Lanfranchi, Valerio Ferrario, Somayyeh Gandomkar, Stefan E. Payer, Erna Zukic, Haris Rudalija, Alexandra Musi, Ines Gaberscek, Yuliya Orel, Doreen Schachtschabel, Christian Willrodt, Michael Breuer, and Wolfgang Kroutil*

Transforming a Historical Chemical Synthetic Route for Vanillin Starting from Eugenol to a Cell-Free Bi-Enzymatic Cascade

Elisa Lanfranchi,[a] Valerio Ferrario,[b] Somayyeh Gandomkar,[a] Stefan E. Payer,[c] Erna Zukic,[a] Haris Rudalija,[a,c] Alexandra Musi,[a,c] Ines Gaberscek,[a,c] Yuliya Orel,[a,c] Doreen Schachtschabel,[b] Christian Willrodt,[b] Michael Breuer,[b] and Wolfgang Kroutil[a,c,d,e],*

Supporting Information

TABLE OF CONTENTS

MATERIAL AND METHODS 3
SUPPLEMENTARY DATA 9
CHROMATOGRAMS - References 27
CHROMATOGRAMS – Cascade and controls at 0.5 mL scale 31
CHROMATOGRAMS – Reactions at 3 mL scale and 50 mM substrate loading 35
CALIBRATION CURVES 36
NMRs 39
LIST OF PROTEIN SEQUENCES 41
LIST OF DNA SEQUENCES 46
LIST OF PLASMIDS 57
REFERENCES 59

MATERIAL AND METHODS

General

General reagents for microbial growth were obtained from Carl Roth GmbH. Catalase, coniferyl alcohol, eugenol and other chemicals were purchased from Sigma Aldrich. A commercial bovine liver catalase was used in all the experiments (Sigma/Merk product# C40, ≥10,000 units/mg protein). HPLC-grade solvents were obtained from Honeywell or Chempur. For plasmid isolation, kits from New England Biolab Inc. or Qiagen GmbH were used. Other reagents for molecular biology and protein analysis were purchased from Thermofisher Scientific or GenScript. Synthetic genes were obtained from Biocat GmbH. DNA sequencing was performed at Microsynth AG. Protein quantification was performed with Bradford method (Bio-Rad Protein Assay Dye Reagent Concentrate).

Buffers and substrate stocks

Unless otherwise stated buffers and stock solutions were prepared according to the following protocols.

Reaction buffer pH 9.0
The reaction buffer was freshly assembled in a dry falcon tube by adding each component in the following order: sodium ascorbate (9 mg, 0.05 mmol, final conc. 1 mM), $FeSO_4*7H_2O$ (13 mg, 0.05 mmol, final conc. 1 mM) and Glycine-NaOH buffer pH 9.0 (50 mL, 50 mM). The solution was then gently mixed by inversion or slow stirring and kept on ice for maximum one working day.

Reaction buffer pH 8.0
The reaction buffer was freshly assembled in a dry falcon tube by adding each component in the following order: sodium ascorbate (9 mg, 0.05 mmol, final conc. 1 mM), $FeSO_4*7H_2O$ (13 mg, 0.05 mmol, final conc. 1 mM) and Tris-HCl buffer pH 8.0 (50 mL, 50 mM). The solution was then gently mixed by inversion or slow stirring and kept on ice for maximum one working day.

Coniferyl alcohol 100 mM (Sigma-Aldrich/Merk product# 223735*)*
Coniferyl alcohol (90 mg, 0.5 mmol, final conc. 100 mM) was dissolved in ethanol (5 mL) and stored at -20 °C. Note that in some experiments DMSO was used as cosolvent instead of ethanol.

Eugenol 100 mM (Sigma Aldrich/Merk product# E51791)
Eugenol (0.077 mL, 0.5 mmol, final conc. 100 mM) was dissolved in ethanol (5 mL) and stored at -20 °C. Note that in some experiments DMSO was used as cosolvent instead of ethanol.

Phylogenetic tree and cluster analysis

Enrichment
During the sequence enrichment phase, the seed sequences were searched in UniProt database to come up with an expanded set of homologues sequences. The search was performed using the HHblits,[1] a sensitive sequence search algorithm based on profile hidden markov models.[2] Homologus sequences were considered according to an E-value[3] threshold of $1e^{-10}$. In this specific case the seed sequences considered were 3 isoeugenol cleavage oxygenases (IECOs) from *Pseudomonas*: *Pseudomonas sp.* (GenBank: AXB59146),[4] *Pseudomonas putida* (GenBank: BAF62888)[5] and *Pseudomonas nitroreducens* (GenBank: ACP17973)[6] as well as the lignostillbene dioxygenase from *Pseudomonas brassicaceraum* (PDB: 5V2D)[7] and the aromatic dioxygenase from *Thermothelomyces*

thermophilus (GenBank: XP_003665585).[8] Finally, an enriched dataset containing in total 16780 protein sequences was obtained.

Clustering

Distance among the different sequences of the dataset were calculated using the clustal Ω algorithm[9] with default settings. Once the global distance matrix for all the sequences was calculated (all vs all calculation), a hierarchical clustering was performed where the most similar sequences or clusters are grouped iteratively into clusters, until all sequences are clustered together at a root. Average linkage method was used to compute the distance from sequences to clusters and from clusters to clusters. Such combination of hierarchical clustering with average linkage is also called UPGMA Algorithm.[10]

Expression of *Rj*EUGO and preparation of cell free extract (CFE)

The codon optimised sequence of *Rj*EUGO was obtained in pBAD vector with a 6XHis-Tag fused at the C-term of the amino acid sequence. Competent cells of *E. coli* NEB 10 Beta were transformed with pBAD_EUGO plasmid and colonies were selected on LB-ampicillin (100 mg /L) agar plates (Protocol from New England Biolabs Inc.; C3019H). *Rj*EUGO was recombinantly expressed according to a previously described protocol.[11] In summary, a preculture was set up in LB-ampicillin (final conc. 100 mg/mL) by resuspending a single colony of *E. coli* NEB 10 Beta harbouring pBAD_EUGO plasmid. After overnight incubation at 37°C, 1% v/v of preculture was transferred to a baffled flask containing fresh TB medium supplemented with ampicillin (100 mg/L). Cells were grown at 37 °C and 120 rpm and induced with arabinose (0.02%) at OD_{600} ~0.6. The protein expression was conducted for c.a. 20 hours at 30 °C. Cell were harvested by centrifugation (R10A3 rotor, 4500 rpm, 4 °C, 15 min), washed once with Tris-HCl (10 mM, pH 8.0) and stored at -20 °C. For the preparation of the CFE, the cell pellet was resuspended in tris-HCl (50 mM, pH 8.0; c.a. 4 mL per g of frozen wet cell weight). Finally, the cells were disrupted by sonication (2 or 3 cycles with short ice incubation in between: 30% amplitude; 1 sec on; 2 sec off; 2 mins total pulse on; 6 mins total time). CFE was separated from the insoluble fraction by centrifugation (R20A2 rotor, 15000 rpm, 30 min, 4 °C), freeze-dried and stored at -20 °C. An example of the results is reported in Table S1.

Expression of alkene cleavage oxygenase variants and preparation of cell free extract (CFE)

The codon optimised sequence of each ACO was obtained in pET-51b(+) vector, with a Strep-Tag-II fused to the N-terminus of the amino acid sequence. Competent cells of *E. coli* BL21(DE3) were transformed via heat shock with the pET-51b(+)_ACO plasmid according to the standard protocol provided by New England Biolabs Inc. (Product number C2527). Finally, positive colonies were selected on LB-ampicillin (100 mg/L) agar plates after overnight incubation at 37 °C. The day later one single colony was resuspended in LB liquid medium (5 mL) supplemented with ampicillin (100 mg /L) and incubated overnight at 37 °C or 24 hours at 30 °C and 120 rpm. Then, 1% v/v of the pre-culture was transferred to a baffled flask containing terrific broth medium (TB) supplemented with ampicillin (100 mg/L), and incubated at 37 °C and 120 rpm. At OD_{600} ~0.5, iron salts were added to the culture to a final conc. of 100 mg/L each [Iron(II)sulphate heptahydrate, ammonium iron (II) sulphate hexahydrate and ammonium iron(III) citrate], then the cells were cooled at 20 °C and 100 rpm for 15-20 min. Protein expression was induced with IPTG (0.5 mM) at OD_{600} ~0.8-1, and the culture was incubated for ~20 hours at 20°C and 100 rpm. At the end of the cultivation, the cells were harvested by centrifugation (R10A3 rotor, 4500 rpm, 4 °C, 15 min), washed once with glycine-NaOH buffer (10 mM, pH 9.0) and stored at -20 °C. Next, the cell pellet was resuspended in the lysis buffer (50 mM

glycine-NaOH, 1 mM $FeSO_4*7H_2O$, 1 mM sodium ascorbate, pH 9.0; ~ 4 mL of buffer per g of frozen wet cell pellet or 10 mL of buffer for 50 mL of cell culture) and disrupted by sonication (2 or 3 cycles with short ice incubation in between: 30% amplitude; 1 sec on; 2 sec off; 2 mins total pulse on; 6 min total time). CFE was separated from the insoluble fraction by centrifugation (R20A2 rotor, 15000 rpm, 30 min, 4 °C) and directly used for biotransformation experiments (fresh CFE) or freeze-dried and stored at -20°C (freeze-dried CFE). An example of the results is reported in Table S1.

Standard enzymatic assay

200X stock solution of coniferyl alcohol (90 mg, 0.5 mmol, final conc. 100 mM) was prepared in ethanol (5 mL) and stored at -20 °C. From this, a new 20X stock solution of coniferyl alcohol (10 mM) was freshly prepared by 1:10 dilution in glycine-NaOH (50 mM, pH 9.0). A cuvette containing 0.750 mL glycine-NaOH (50 mM, pH 9.0) and 0.05 mL of the substrate 20X stock was pre-warmed at 25 °C for 1.5 mins. The reaction was initiated by the addition of 0.2 mL of the enzyme sample. The cleaving activity was measured spectrophotometrically following the production of vanillin at 349 nm (ε_{349}=26.7 $mM^{-1}cm^{-1}$). One unit of activity (U) is defined as the amount of enzyme required to produce 1 µmol of vanillin per min. Agilent Cary 60 UV-Vis spectrophotometer was used for all the measurements. 2 mg/mL of freeze-dried CFE was found a suitable concentration to obtain good curves in the range of 0-15 mU/mg.

Biotransformation of coniferyl alcohol to vanillin with *Pn*IECO

The reactions (final vol. 0.5 mL) were set up in 1.5 mL glass vials as follows: fresh CFE containing *Rj*IECO (0-4 mg/mL) was diluted in the reaction buffer pH 9.0 together with bovine liver catalase (final conc. 0-10 mg/mL, 0-100 kU/mL). The reaction was initiated by the addition of coniferyl alcohol (0.025 mL from 100 mM stock, final conc. 5 mM). Note that the reaction contained 5% v/v ethanol deriving from the substrate stock. Each vial was incubated at 30 °C and 120 rpm (rotary shaker with a 25 mm shaking diameter) for 24 hours. To improve the oxygenation, the vials were placed horizontally. At certain time points, reaction samples (0.2 mL) were taken and quenched with methanol (0.4 mL, 2 vol. equiv.). The mixture was centrifuged for 5-10 mins at 20238 x g. Then supernatant was finally transferred in a HPLC vial for analysis.

Biotransformation of eugenol to vanillin (on-pot cascade with 5 mM substrate loading)

Unless otherwise stated, the reactions (final vol. 0.5 mL) were set up in 1.5 mL glass vials as follows: *Rj*EUGO (freeze-dried CFE, 1 mg/mL), *Pn*IECO or ACO-## (fresh or freeze-dried CFE, 6 mg/mL) and bovine liver catalase (10 mg/mL, ≥100 kU/mL) were mixed in the reaction buffer pH 9.0 or pH 8.0. The reaction was initiated by the addition of eugenol (0.025 mL from 100 mM stock, final conc. 5 mM). Note at the final content of cosolvent (5% v/v) from the substrate stock. Each vial was incubated at 30 °C and 120 rpm for 18-24 hours (rotary shaker with a 25 mm shaking diameter). To improve oxygenation, the vials were placed horizontally. At certain time points, reaction samples (0.2 mL) were quenched with methanol (0.4 mL, 2 vol. equiv.) and the mixture was centrifuged at 20238 x g for 5-10 mins. Finally, the supernatant was analysed by HPLC.

ACO-03/ACO-03 C26N characterization

Unless otherwise stated under the respective figure/table, the reactions (final vol. 0.5 mL) were set up in 4 mL glass vials as follows: ACO-03 (freeze-dried CFE, 10 mg/mL) was dissolved in the reaction buffer

pH 8.0 together with bovine liver catalase (final conc. 10 mg/mL, ≥100 kU/mL). The reaction was initiated by the addition of coniferyl alcohol (0.025 mL from 100 mM stock, final conc. 5 mM or 0.025 mL from 1 M stock in DMSO, final conc. 50 mM). Note that the reaction contained 5% v/v ethanol or DMSO deriving from the substrate stock. Each vial was incubated for 4 hours at 30 °C and 120 rpm (rotary shaker with a 25 mm shaking diameter). To improve the oxygenation, the vials were placed horizontally. At certain time points, reaction samples (0.2 mL) were taken and quenched with methanol (0.4 mL, 2 vol. equiv.). The mixture was centrifuged for 5-10 mins at 20238 x g. Then supernatant was finally transferred in a HPLC vial for analysis.

ACO-03/ACO-03 C26N stability

Unless otherwise stated under the respective figure/table, the stability tests were carried out as following. ACO-03 (freeze-dried CFE, 5X stock conc. 50 mg/mL) and bovine liver catalase (5X stock conc. 50 mg/mL) were freshly reconstituted in the reaction buffer pH 8 and kept on ice. Twelve identical reactions (final vol. 0.5 mL) were set up in 4 mL glass vials as follows: ACO-03 (0.1 mL of the 5X stock, 5 mg, final conc. 10 mg/mL), bovine liver catalase (0.1 mL of the 5X stock, 5 mg, ≥ 100 kU/mL, final conc. 10 mg/mL) were mixed in the reaction buffer pH 8. Then, the vials were incubated at 30 °C and 120 rpm for 0, 15, 30, 60, 120, 240 min (rotary shaker with a 25 mm shaking diameter). At the end of each incubation time, the reaction was initiated by the addition of coniferyl alcohol (0.025 mL of the 100 mM stock, 0.0025 mmol, final conc. 5 mM). Due to the substrate stock, the mixture also contained 5% v/v ethanol. Then the reactions were incubated for 3 hours horizontally at 30 °C and 120 rpm. Samples (0.2 mL) were quenched with methanol (0.4 mL, 2 vol. equiv.), centrifuged at 20238 x g for 10 mins and finally, vanillin formation was followed by HPLC at 280 nm.

Stepwise cascade with 50 mM substrate loading

A stock solution of eugenol (1 M) was prepared in DMSO. *Rj*EUGO (freeze-dried CFE, 5X stock conc. 50 mg/mL), ACO-03 C26N (freeze-dried CFE, 3.33X stock conc. 50 mg/mL) and catalase (5X stock conc. 50 mg/mL) were freshly reconstituted in the reaction buffer pH 8 or pH 9. The cascade was performed in one-pot two step fashion. In the first step, reaction mixtures (500 µL) were set up in 4 mL glass vials as follows: *Rj*EUGO (100 µL of the stock solution, 5 mg, final conc. 10 mg/mL), bovine liver catalase (100 µL of the stock solution, 5 mg, ≥ 100 kU/mL, final conc. 10 mg/mL), and eugenol (25 µL of the stock solution, final conc. 50 mM) were mixed in the reaction buffer pH 8 or pH 9. Then the vials were incubated at 30 °C and 120 rpm (25 mm rotary shaker) for 17 hours. Once the first step went to completion, ACO-03 C26N (150 µL of the stock solution, 7.5 mg, final conc. 15 mg/mL), bovine liver catalase (100 µL of the stock solution, 5 mg, ≥ 100 kU/mL, final conc. 10 mg/mL), and DTT (5 µL of 100 mM stock solution, final conc. 1 mM) were added to the reaction mixture. The vials were again incubated at 30 °C and 120 rpm (25 mm rotary shaker) for 17 hours. Every 2 hours from starting the reaction till 7 hours, 1 mM $FeSO_4$ (5 µL of the stock solution, final conc. 1 mM) was added to the reaction mixture. Samples (100 or 200 µL) were quenched with methanol (800 or 400 µL), centrifuged at 20238 x g for 10 mins and finally, the supernatant was analysed by HPLC.

Biotransformation of coniferyl alcohol to vanillin with oxygen bubbling

Stock solution of coniferyl alcohol (90 mg, 0.499 mmol, final conc. 1 M) was prepared in DMSO (0.5 mL) and stored at -20°C. For HPLC analysis, samples (0.2 mL) were taken, quenched with methanol (0.4 mL, 2 vol. equiv.) and centrifuged at 20238 x g for 10 mins.

3 mL scale

ACO-03 C26N (freeze-dried CFE, 2X stock conc. 50 mg/mL) and catalase (5X stock conc. 50 mg/mL) were freshly reconstituted in the reaction buffer pH 8. The reaction was set up in 10 mL round bottom flask. Specifically, ACO-03 C26N (1.5 mL of the stock solution, 75 mg, final conc. 25 mg/mL), bovine liver catalase (0.6 mL of the stock solution, 30 mg, ≥ 100 kU/mL, final conc. 10 mg/mL), DTT (0.03 mL of 100 mM stock solution in water, final conc. 1 mM) and coniferyl alcohol (0.150 mL of 1 M stock solution, final conc. 50 mM) were mixed in the reaction buffer pH 8. Due to the substrate stock, the mixture also contained 5% v/v DMSO. The reaction was incubated at 30°C using a water bath stirred for 17 hours. Oxygen was bubbled for the first 7 hours, then the reaction vessel was closed for additional 10 hours. Every 2 hours, $FeSO_4$ (0.03 mL of a 100 mM stock solution in buffer, final conc. 1 mM) was added to the reaction.

10 mL scale

The reaction was set up in a 50 mL Erlenmeyer flask. ACO-03C26N (free-dried CFE 150 mg, reconstituted in 4 mL buffer, final conc. 15 mg/mL), bovine liver catalase (100 mg, reconstituted in 3 mL buffer, final conc. 10 mg/mL), DTT (0.1 mL of the 100 mM solution, final conc. 1 mM) and coniferyl alcohol (0.5 mL of 1 M stock solution, final conc. 50 mM) were mixed in the reaction buffer pH 8. Due to the substrate stock, the mixture also contained 5% v/v DMSO. The reaction (was incubated at 30 °C using water bath and stirred for 17 hours. Oxygen was bubbled for 6 hours, then the reaction vessel was closed for the other 11 hours. Every 2 hours, $FeSO_4$ (100 µL of the stock solution, final conc. 1 mM) was added to the reaction.

Semi-preparative biotransformation of eugenol to coniferyl alcohol with *Rj*EUGO, product isolation and purification

The biotransformation (total volume 3 mL) was performed in a 10 mL round bottom flask with a stir bar. Eugenol (0.023 mL, 25 mg, 0.15 mmol, 1.0 equiv., 50 mM final conc.) was dissolved in DMSO (0.150 mL). Then, the solution was diluted with tris-HCl buffer (50 mM, pH 8.0, 2.827 mL). To obtain a homogeneous dispersion of the eugenol droplets, the mixture was stirred for about 15–20 mins. Then bovine liver catalase (30 mg, ≥300 kU) and *Rj*EUGO (30 mg of freeze-dried CFE, tris base salt content was neglected in the calculation) were added consecutively (note that *Rj*EUGO was added after the catalase had completely dissolved). The reaction was incubated at room temperature and gentle stirring (40 rpm) for 17–24 hours. At certain time points, samples (0.1 mL) were quenched with methanol (0.8 mL, 8 vol equiv.). The samples were centrifuged at 20238 × g for 10 mins and the supernatant was analyzed by HPLC. When the reaction was completed, the products were extracted with ethyl acetate (3 × 1.5 mL EtOAc, in 2 mL microcentrifuge tubes). The aqueous and organic phases were separated by centrifugation (20238 × g for ~ 3 mins). Then, the organic phase was dried with $NaSO_4$ and concentrated under reduced pressure (40 °C, 200 mbar), but not dried completely to avoid polymerization. The concentrate was directly purified by silica gel chromatography (10 mL column volume) using a mixture of 1:1 etylacetate:cyclohexane as eluent. Fractions containing coniferyl

alcohol (according to TLC) were combined and concentrated under reduced pressure (40 °C, 200 mbar). Final drying under high vacuum (1.5 mbar) and inert gas flow yielded the title compound as yellowish solid (24.2 mg, 0.134 mmol, 90% yield). Notes: The obtained product was stored at -20 °C under inert gas atmosphere. Evaporation of a product solution in chloroform induced polymerization, therefore, no crude NMR was taken before product purification. ^{1}H NMR (300 MHz, $CDCl_3$) δ 6.94 – 6.83 (m, 3H), 6.52 (dt, *J* = 15.9, 1.5 Hz, 1H), 6.21 (dt, *J* = 15.9, 5.9 Hz, 1H), 5.75 (s, 1H), 4.29 (dd, *J* = 5.9, 1.5 Hz, 2H), 3.89 (s, 3H), 1.78 (s, 1H). Data matches a commercial reference compound (Figure S35) and literature.[12]

Semi-preparative biotransformation of coniferyl alcohol to vanillin with ACO-03 C26N, product isolation and purification

The biotransformation (total volume 10 mL) was performed in a 100 mL baffled flask closed with a gas permeable adhesive seal (Thermofisher product# 241205). ACO-03 C26N (freeze-dried CFE 100 mg, final conc. 10 mg/mL), bovine liver catalase (100 mg, ≥100 kU, final conc. 10 mg/mL) and *Rj*EUGO (freeze-dried CFE 10 mg, final conc. 1 mg/mL) were mixed in the reaction buffer pH 8, supplemented with DTT (0.01 mL from 1 M substrate stock in water, 0.01 mmol, final conc. 1 mM). Once the solution looked homogeneous, coniferyl alcohol was dissolved in 0.5 mL of DMSO and straight added to the reaction mixture (90 mg, 0.499 mmol, final conc. 50 mM). The flask was closed and incubated at 30 °C and 180 rpm (rotary shaker with a 25 mm shaking diameter). Samples were taken for HPLC analysis (0.1 mL), quenched with acetonitrile with 0.1% TFA (0.9 mL, 9 equiv. vol.) and centrifuged for 20230 x g for 10 mins. After 20 hours, the solution was acidified with few drops of HCl and extraction was performed with ethyl acetate (total vol. 15 mL). The aqueous and organic phases were separated by centrifugation (20238 × g for ~ 3 mins). Then, the organic phase was dried with $NaSO_4$ and concentrated under reduced pressure (40 °C, start from 200 mbar to 50 mbar). Reaction products and unreacted substrate were purified by flash chromatography (Biotage® Sfär Duo) using a 5 g silica gel column (product # FSRS-0445-0005). For the separation a mixture of ethyl acetate in cyclohexane was applied with the following method: 4-4% EtOAc 1CV, 4-15% EtOAc 2CV, 15-100% EtOAc 8 CV, 100-100% EtOAc 1.8 CV and 100-100% EtOAc 3.2CV. Fractions containing vanillin only (according to TLC) were combined and dried under reduce pressure and air flow. The main product vanillin was a solid (8 mg, 0.052 mmol, 10.5% yield). ^{1}H NMR (300 MHz, $CDCl_3$) δ 9.83 (s, 1H), 7.47 – 7.38 (m, 2H), 7.04 (d, *J* = 8.5 Hz, 1H), 6.23 (s, 1H), 3.97 (s, 3H). ^{13}C NMR (75 MHz, CDCl3) δ 191.1, 151.8, 147.3, 130.0, 127.7, 114.5, 108.9, 56.3. The NMR data are in accordance with literature.[13]

HPLC Analysis

The analysis of the reaction was carried out using Agilent Technologies 1260 Infinity HPLC system, equipped with a DAD detector. Compounds were separated by reversed phase with Luna® 5 µm C18(2) 100 Å column, (length 250 mm; internal diameter 4.6 mm; product 00G-4252-E0, Phenomenex®). The mobile phase was composed of water (Eluent A) and acetonitrile (Eluent B) both supplemented with 0.1% trifluoracetic acid. Unless otherwise stated, the following method was set: flow 1 mL/min; injection 5 µL; 0 min 70% A + 30% B; 2 min 70% A + 30% B; 10 min 100% B; 15 min 100% B; 17 min 70% A + 30% B; 22 min 70% A + 30% B. Eugenol, coniferyl alcohol and vanillin were detected at 280 nm, whereas coniferyl aldehyde was followed at 340 nm.

SUPPLEMENTARY DATA

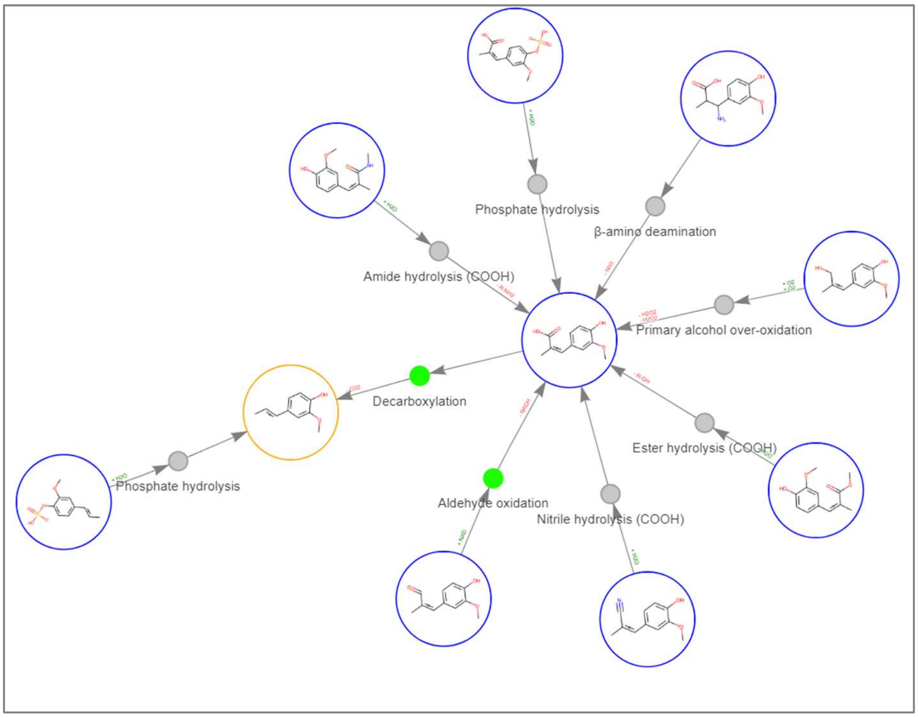

Figure S1. Retro-synthesis analysis of isoeugenol with RetroBioCat tool (Network explorer option).

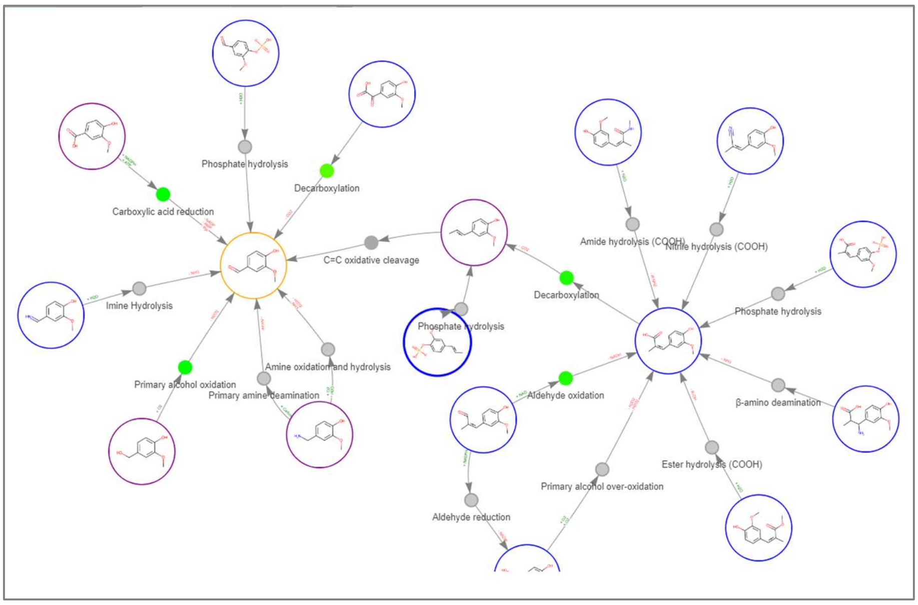

Figure S2. Retro-synthesis analysis of vanillin with RetroBioCat tool (Network explorer option). The oxidative C=C cleavage step was inserted manually.

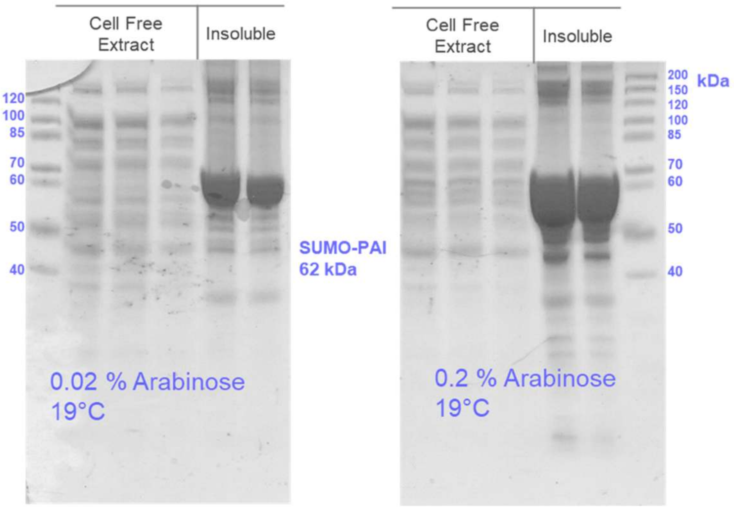

Figure S3A. SDS-PAGE of the fatty acid isomerase from *Cutibacterium acnes* (*Ca*PAI; PDB: 2BAB; MW plus His-SUMO tag 62 kDa) recombinantly expressed in *E. coli* NEB10β after arabinose induction. The protein is only visible in the insoluble fraction (IB) and not in the cell free extract (S).

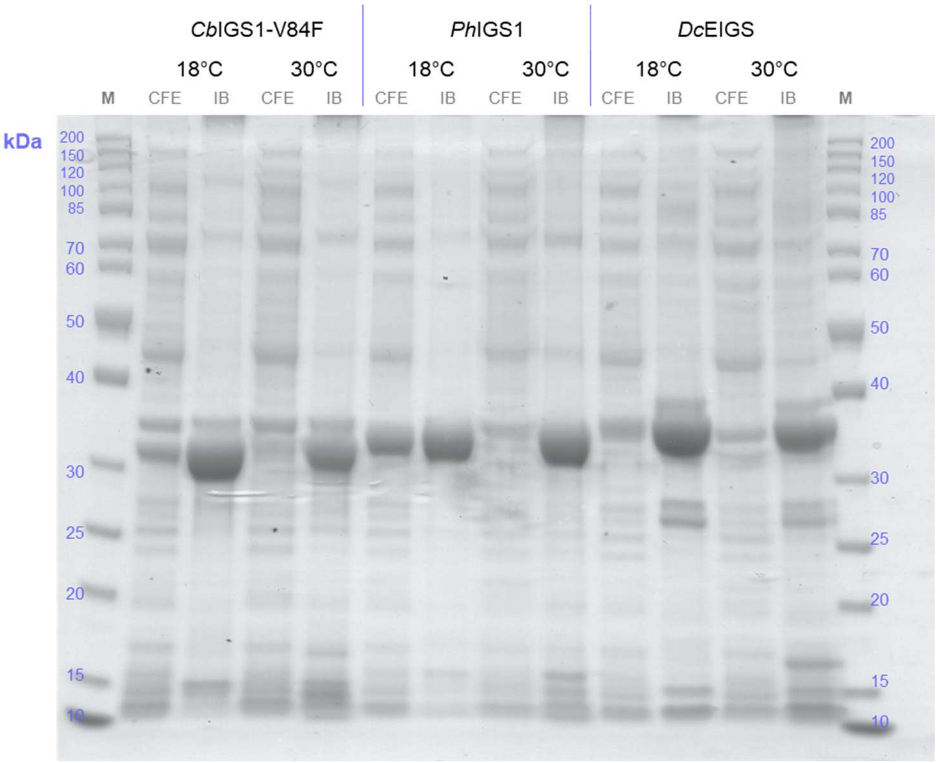

Figure S3B. SDS-PAGE of recombinant expression of isoeugenol synthases in *E. coli* BL21 (DE3). Page Ruler Unstained Protein Ladder, ThermoFisher Scientific (M). 15 µg of cell free extract (CFE) or inclusion bodies (IB) were loaded in each lane. CbIGS1-V84F 36 kDa, PhIGS1 37 kDa and DcEIGS 38 kDa. Gel staining was performed with Coomassie Blue.

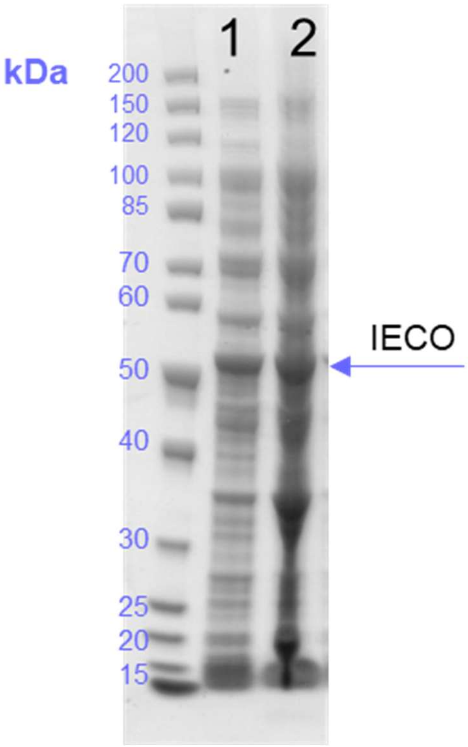

Figure S3C. SDS-PAGE of isoeugenol cleavage oxygenase (IECO) from *Pseudomonas nitroreducens* recombinantly expressed in *E. coli* BL21(DE3). Lane 1: cell free extract; Lane 2: insoluble fraction.

Table S1. Examples of CFE preparation. n.d. not determined.

**Enzyme**	***Rj*EUGO**	***Pn*IECO**	**ACO-03**	
Culture	0.08 L	0.05 L	0.05 L	0.15 L
Wet Cell Weight	1.65 g	1.11 g	1.77 g	6.45 g
Lysis Buffer	11 mL	10 mL	13 mL	30 mL
Freeze-dried powder	210 mg	230 mg	220 mg	890 mg
Buffer Content (w/w)	6 %	22 %	25 %	14 %
Specific Activity (mU/mg of FD-CFE[a])	382 mU/mg	2.8 mU/mg	10 mU/mg	10 mU/mg

[a] mg of FD-CFE refers to the effective freeze-dried powder, which also contains buffer salts.

Table S2. Enzymatic cleavage of coniferyl alcohol **4** with *Pn*IECO (unoptimized reaction conditions).[a]

	CFE Preparation[b]	CFE (mg/mL)	Conversion of 4 [%]	HPLC-Yield of 3 [%]
***Pn*IECO**	Fresh	1	24	9
	Fresh	2	45	17
**Control[d]**	Fresh	1	31	≤1
	Fresh	2	49	≤ 1

[a] The biotransformation (total vol. 1 mL) was carried out in 4 mL glass vials: *Pn*IECO (CFE preparation) was mixed in glycine-NaOH (100 mM, pH 9.0) and $FeSO_4$ (1 mM). The reaction was initiated by the addition of coniferyl alcohol (0.05 mL from 100 mM stock in ethanol, final conc. 5 mM). Incubation: 30°C and 120 rpm, 16 hours.

[b] Lysis buffer: Potassium phosphate (40 mM, pH 7.5), $FeSO_4$ (1 mM), sodium ascorbate (1 mM).

[c] Not determined.

[d] Control: Cell free extract of empty *E. coli* BL21(DE3).

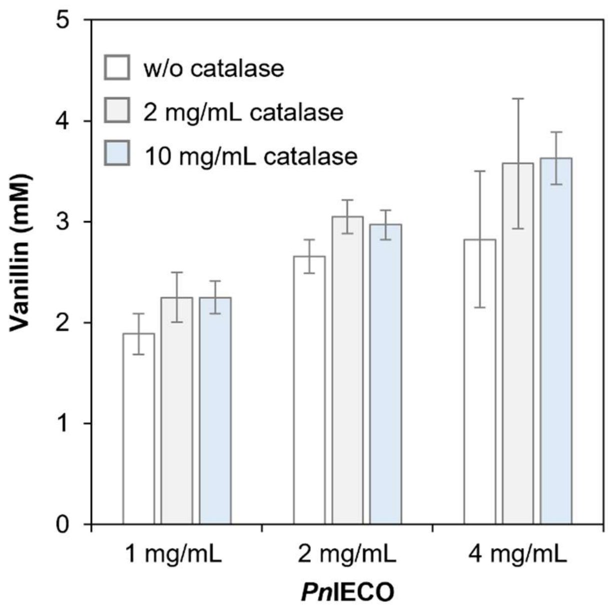

Figure S4. Oxidative cleavage of coniferyl alcohol to vanillin at varied amount of *Pn*IECO and catalase. Each reaction (0.5 mL) was set up in 1.5 mL glass vials with the following conditions: Glycine-NaOH buffer (50 mM, pH 9.0), $FeSO_4$ (1 mM), sodium ascorbate (1 mM), 5% v/v ethanol, *Pn*IECO (fresh CFE 1-4 mg/mL), bovine liver catalase (Sigma C40; 0-10 mg/mL = 0 to ≥100 kU/mL) and coniferyl alcohol (5 mM). Each reaction was incubated at 30°C and 120 rpm for 24 hours. Error bars indicates the standard deviation from at least three independent experiments.

Table S3. One-pot bioconversion of eugenol to vanillin with fresh cell free extract.[a]

***Rj*EUGO** (mg/mL)	***Pn*IECO** (mg/mL)	**Cosolvent** 5% v/v	**Distribution** **1 [%]**	**4 [%]**	**3 [%]**	**5 [%]**	**HPLC-yield of 3 [%]**
1	4	EtOH	4	38	55	3	48
0	4		97	2≤	1≤	1≤	1≤
1	0		1≤	97	1≤	2	1≤
1	4	DMSO	3	49	44	4	42
0	4		97	2≤	1≤	1≤	1≤
1	0		1≤	96	1≤	2	1≤

[a] Reaction conditions (0.5 mL in 1.5 mL glass vials): *Rj*EUGO (fresh CFE), *Pn*IECO (fresh CFE) and catalase (10 mg/mL) were mixed in the reaction buffer pH 8 or pH 9. The reaction was initiated by the addition of eugenol (0.025 mL, from 100 mM stock in organic solvent, final conc. 5 mM). Incubation at 30°C and 120 rpm overnight.

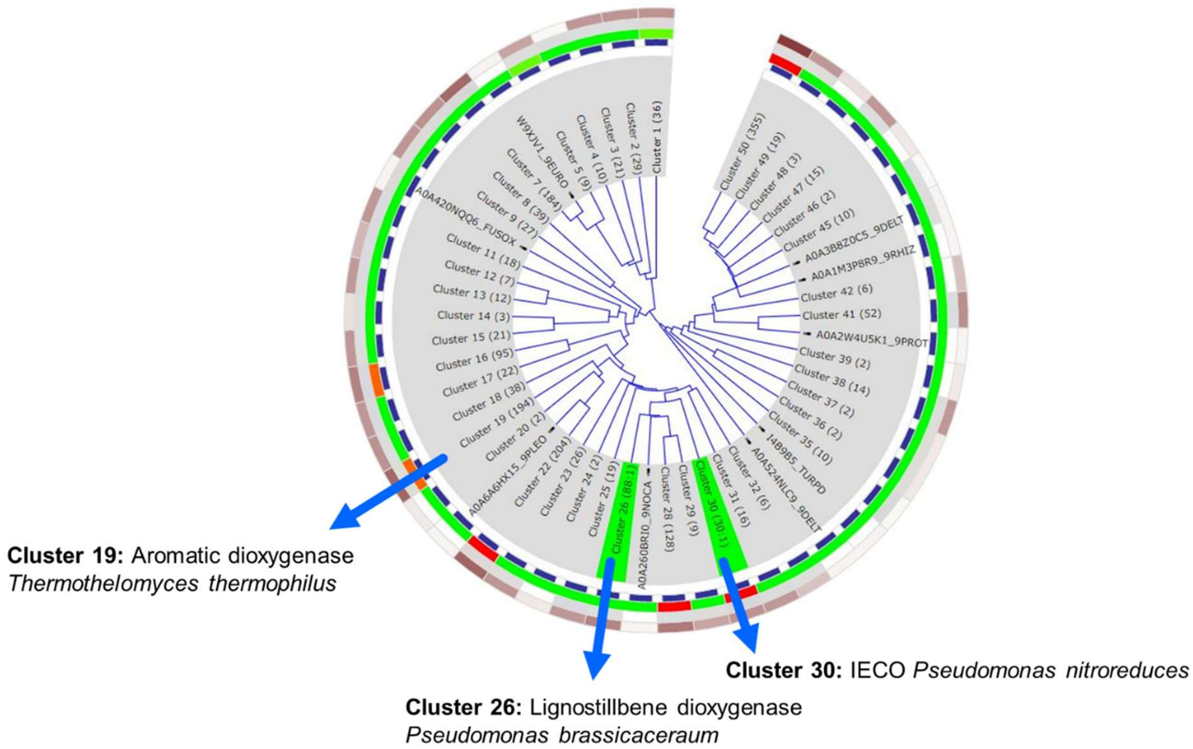

Figure S5. Cluster analysis of carotenoid oxygenase family.

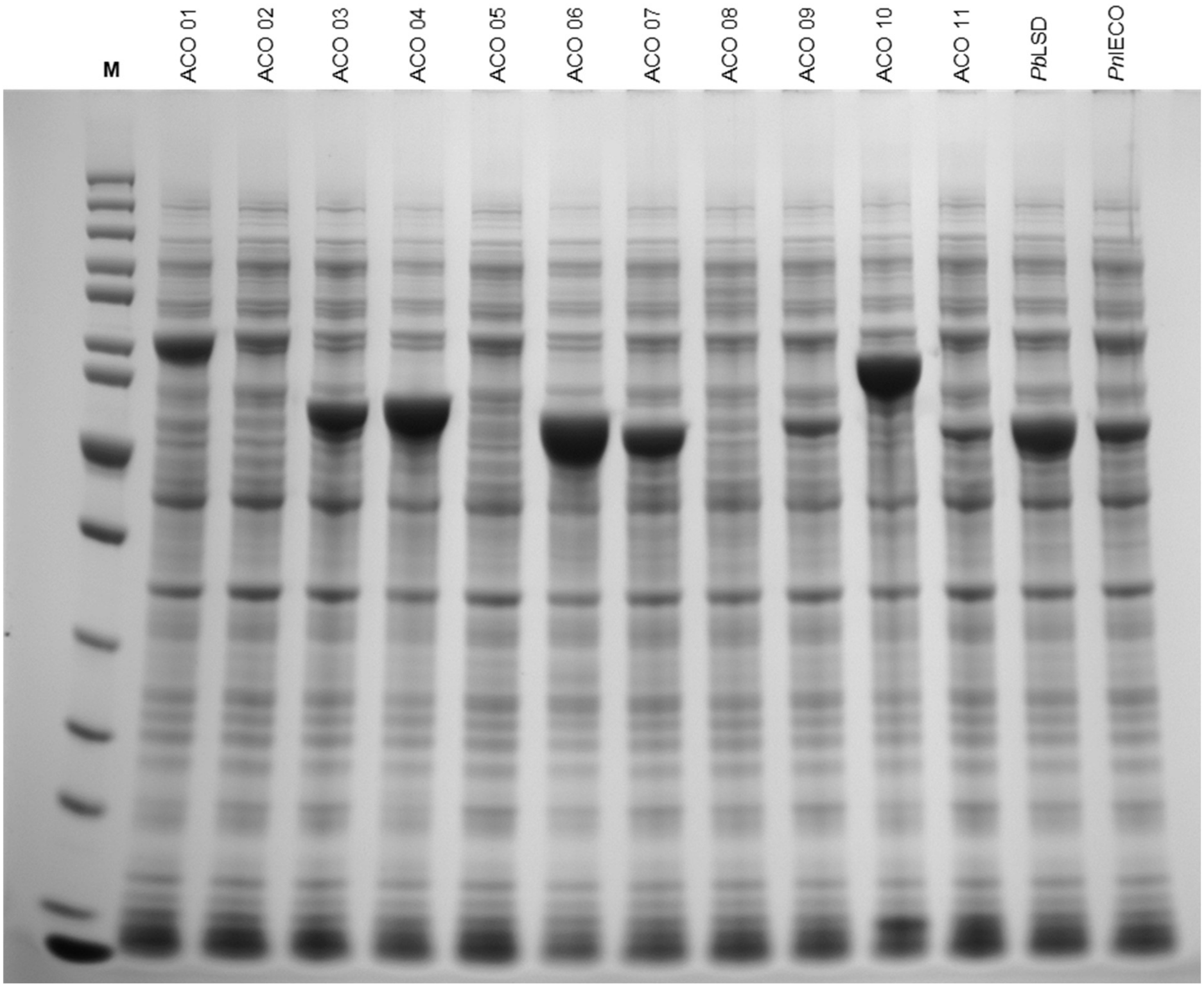

Figure S6. SDS-PAGE of 15 µg cell free extract of *PnI*ECO and its homologs. **A**CO-01+Tag: 69.8 kDa; ACO-02+Tag: 56.6 kDa; ACO-03+Tag: 58.3 kDa; ACO-04+Tag: 57.9 kDa; ACO-05+Tag: 56.7 kDa; ACO-06+Tag: 57.8 kDa; ACO-07+Tag: 56.9 kDa; ACO-08+Tag: 56.3 kDa; ACO-09+Tag: 58.0 kDa; ACO-10+Tag: 63.8 kDa; ACO-11+Tag: 56.7; *Pb*LSD+Tag: 56.3 kDa; *Pn*IECO+Tag: 55.7 kDa. Protein Marker (M): PageRuler™ Unstained Protein Ladder (Thermofisher Scientific).

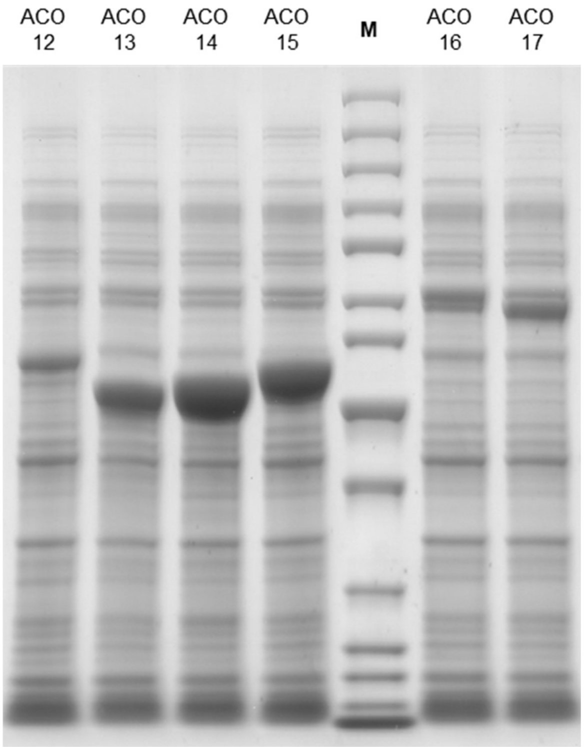

Figure S7. SDS-PAGE of cell free extract of *Pn*IECO homologs. ACO-12+Tag: 56.5 kDa; ACO-13+Tag: 56.9 kDa ACO-14+Tag: 57.7; ACO-15+Tag: 57.9 kDa; ACO-16+Tag: 68 kDa; ACO-17+Tag: 68.8 kDa. Protein Marker (M): PageRuler™ Unstained Protein Ladder (Thermofisher Scientific).

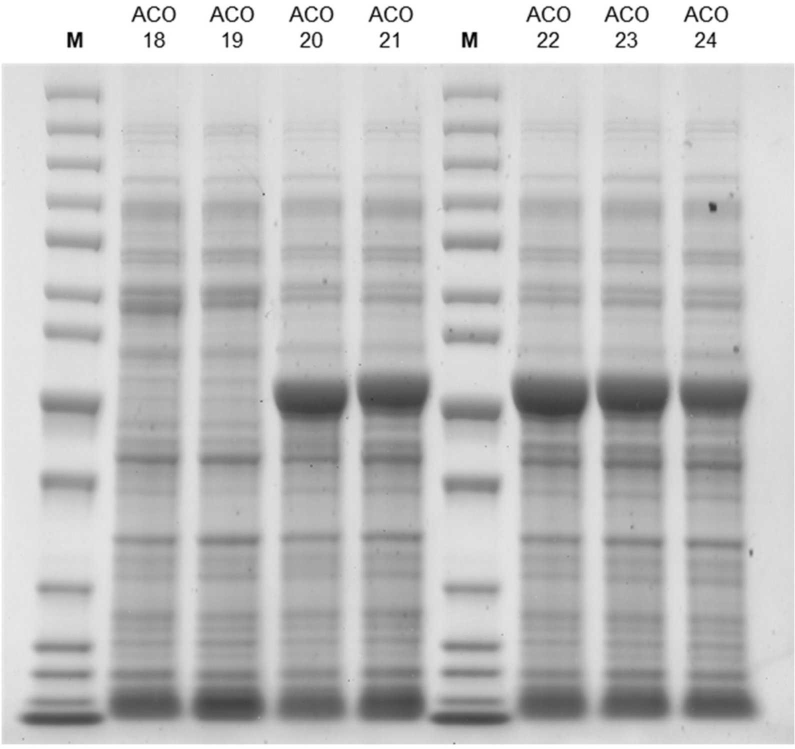

Figure S8. SDS-PAGE of cell free extract of *Pn*IECO homologs. ACO-18+Tag: 65.8 kDa; ACO-19+Tag: 62.6 kDa; ACO-20+Tag: 56.6 kDa; ACO-21+Tag: 56.5 kDa; ACO-22+Tag: 56.6 kDa; ACO-23+Tag: 56.6 kDa; ACO-24+Tag: 56.5 kDa. Protein Marker (M): PageRuler™ Unstained Protein Ladder (Thermofisher Scientific).

Table S4. Screening study of *Pn*IECO homologs.

Name	Reference ID[a]	Recombinant Expression	Cleaving Activity	
*Pn*IECO	ACP17973	Soluble	Active	2.7±0.4 mU/mg
*Pp*IECO	BAF62888	Not Soluble	--	--
*Ps*IECO	AXB59146	Not Soluble	--	--
*Pb*LSD	WP_025212951	Soluble	Not Detected	--
*Tt*Ado (ACO-01)	XP_003665585	Soluble	Active	0.2 mU/mg
ACO-02	WP_147259562.1	Not Soluble	--	--
ACO-03	RYY27909.1	Soluble	Active	10.5±0.5 mU/mg
ACO-04	OZC83942.1	Soluble	Not Detected	--
ACO-05	MQA07219.1	Not Soluble	--	--
ACO-06	WP_056769023.1	Soluble	Active	0.4 mU/mg
ACO-07	WP_142260794.1	Soluble	Active	4.6±0.3 mU/mg
ACO-08	WP_144636993.1	Not Soluble	--	--
ACO-09	WP_132834447.1	Soluble	Not Detected	--
ACO-10	PWB97660.1	Soluble	Not Detected	--
ACO-11	WP_145858760.1	Soluble	Not Detected	--
ACO-12	WP_205415366.1	Not Soluble	--	--
ACO-13	PZN39307.1	Soluble	Active	6±0.8 mU/mg
ACO-14	WP_013078881.1	Soluble	Active	0.3 mU/mg
ACO-15	WP_286867133.1	Soluble	Active	0.4 mU/mg
ACO-16	KUI72511.1	Soluble	Active	0.3 mU/mg
ACO-17	RKU43285.1	Soluble	Active	0.1≤ mU/mg
ACO-18	POS74216.1	Not soluble	--	--
ACO-19	TKA78910.1	Not soluble	--	--
ACO-20	WP_105347798.1	Soluble	Active	0.2 mU/mg
ACO-21	KNH27077.1	Soluble	Not Detected	--
ACO-22	n.a.[b]	Soluble	Active	0.1≤ mU/mg
ACO-23	WP_135846139.1	Soluble	Active	0.1≤ mU/mg
ACO-24	WP_079302303.1	Soluble	Active	0.1≤ mU/mg

[a] NCBI Database.
[b] Not present in the NCBI database.

	ACO 20	ACO 22	ACO 23	ACO 24	ACO 16	ACO 17	*Tt*ADO	ACO 07	ACO 13	ACO 15	ACO 06	ACO 14	*Pn*IECO	ACO 03
**ACO 20**	100	89.2	89.7	88.8	42.7	41.9	43.1	42.8	42.6	38.2	39.5	39.2	41.2	37.4
**ACO 22**	89.2	100	93.2	91.9	43.8	42.4	42.6	41.4	41.4	38.7	39.8	39.8	39.7	36.4
**ACO 23**	89.7	93.2	100	94.6	42.5	41.7	42.5	41.5	40.9	37.1	38.4	38.2	40.3	35.9
**ACO 24**	88.8	91.9	94.6	100	42.6	42	42.2	41	40.8	38.5	39.1	38.5	39.9	37.3
**ACO 16**	42.7	43.8	42.5	42.6	100	72.6	74.9	39.6	39.2	33.3	33.9	34.1	36	33.3
**ACO 17**	41.9	42.4	41.7	42	72.6	100	75.2	40.2	39.8	35.3	35.5	35.3	36.6	34.4
***Tt*ADO**	43.1	42.6	42.5	42.2	74.9	75.2	100	38.3	38.7	34.9	36.1	35.3	37.2	35.3
**ACO 07**	42.8	41.4	41.5	41	39.6	40.2	38.3	100	95	51.8	52.6	53.9	40.4	37.2
**ACO 13**	42.6	41.4	40.9	40.8	39.2	39.8	38.7	95	100	52.8	53.4	54.5	40.6	37.8
**ACO 15**	38.2	38.7	37.1	38.5	33.3	35.3	34.9	51.8	52.8	100	84.3	83.6	41.6	37.2
**ACO 06**	39.5	39.8	38.4	39.1	33.9	35.5	36.1	52.6	53.4	84.3	100	86.5	42	38.4
**ACO 14**	39.2	39.8	38.2	38.5	34.1	35.3	35.3	53.9	54.5	83.6	86.5	100	41.2	37.8
***Pn*IECO**	41.2	39.7	40.3	39.9	36	36.6	37.2	40.4	40.6	41.6	42	41.2	100	47.6
**ACO 03**	37.4	36.4	35.9	37.3	33.3	34.4	35.3	37.2	37.8	37.2	38.4	37.8	47.6	100

Figure S9. Pairwise identity matrix of cleavage oxygenase active towards coniferyl alcohol.

Table S5. Comparison of *Pn*IECO and ACO-03 in the one pot cascade.[a]

**Cleavage Oxygenase**	**pH**	**Distribution**				**HPLC-Yield of 3 [%]**
		**1 [%]**	**4 [%]**	**3 [%]**	**5 [%]**	
***Pn*IECO**	8.0	1≤	51±2	34±2	15±3	31±2
	9.0	1≤	22±2	59±1	18±1	52±3
**ACO-03**	8.0	1≤	14±3	80±2	9±1	70±3
	9.0	1≤	7±2	83±3	10±1	70±3

[a] Reaction conditions (0.5 mL in 1.5 mL glass vials): *Rj*EUGO (freeze-dried CFE 1 mg/mL), *Pn*IECO/ACO-03 (freeze-dried CFE 6 mg/mL) and bovine liver catalase (10 mg/mL) were mixed in the respective reaction buffer. The reaction was then initiated by the addition of eugenol (0.025 mL from 100 mM stock in ethanol, final conc. 5 mM). Incubation: 30°C and 120 rpm, overnight. To improve oxygenation, the vials were placed horizontally. The reported results were obtained from an average of four replicates.

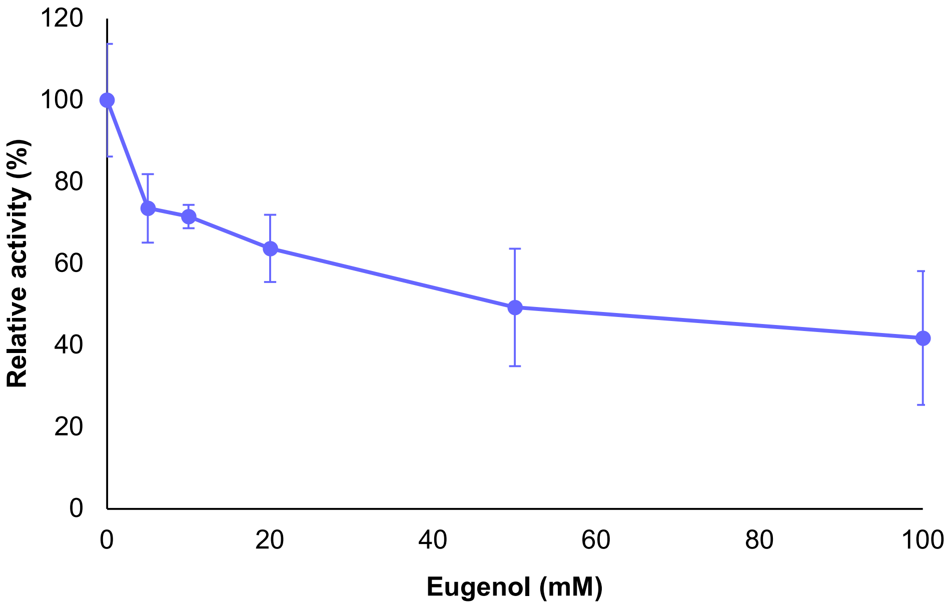

Figure S10. ACO-03 inhibition by eugenol **1**. ACO-03 (CFE 10 mg/mL) and bovine liver catalase were mixed with eugenol (0-100 mM) and coniferyl alcohol (0.025 mL from 100 mM stock in ethanol, final conc. 5 mM) in reaction buffer pH 8.0. Incubation: 4 hours at 30°C and 120. The error bars indicate the standard deviation of three replicates.

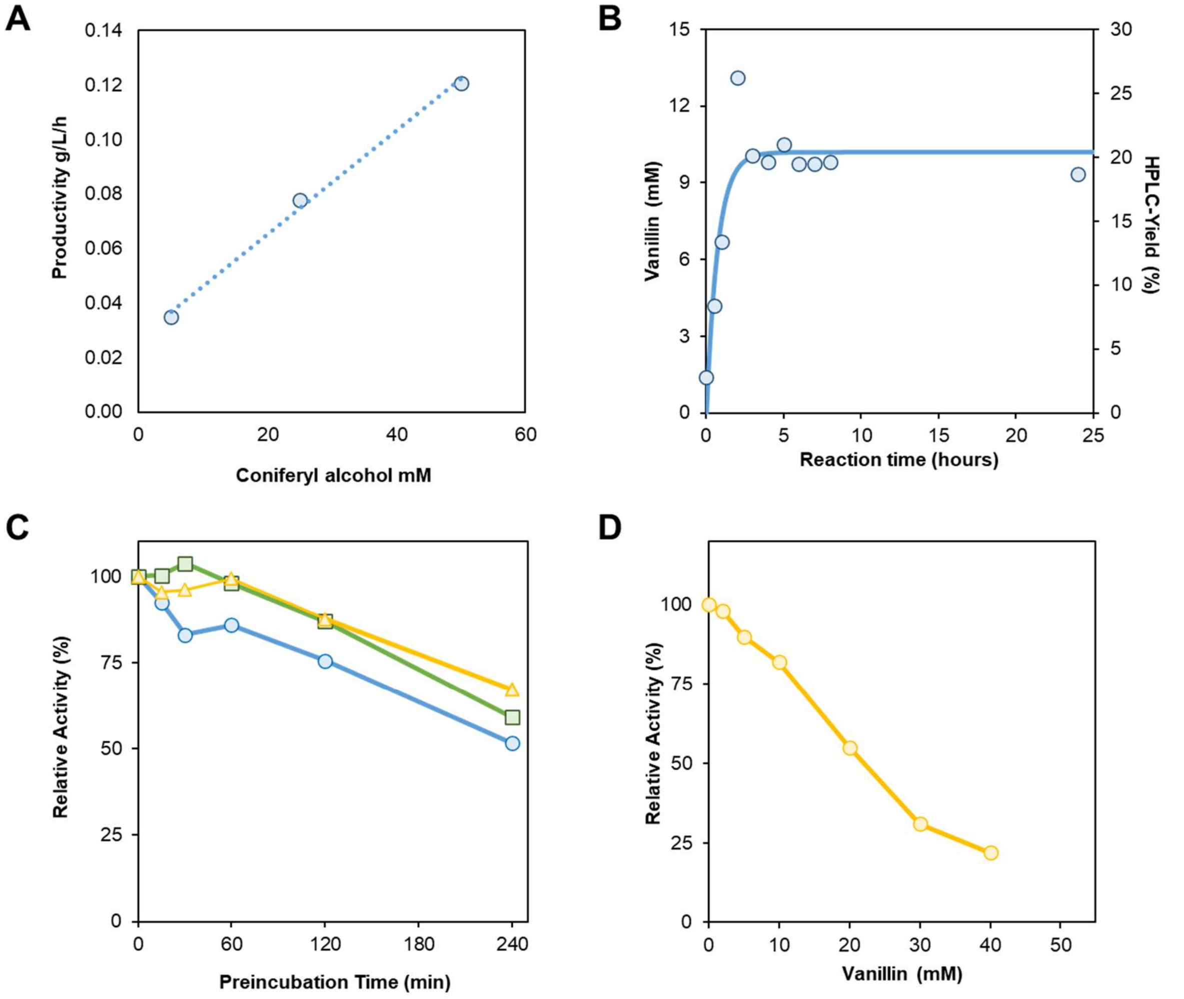

Figure S11. Characterization of ACO-03. **A: A**CO-03 productivity *vs* substrate loading. Reaction conditions: ACO-03 (freeze-dried CFE, 25 mg/mL), bovine liver catalase (10 mg/mL), sodium ascorbate (10 mM), FeSO4 (1 mM), DTT (1 mM), 5% v/v DMSO and coniferyl alcohol (5-20 mM) in Tris-HCl (50 mM, pH 8.0). **B:** Time study for conversion of coniferyl alcohol (50 mM) to vanillin using ACO-03 in glass vials. Reaction condition: ACO-03 (freeze-dried CFE 25 mg/mL), bovine liver catalase (10 mg/mL) were mixed in tris-HCl buffer (50 mM, pH 8.0) containing FeSO4 (1 mM), sodium ascorbate (10 mM) and 5% v/v DMSO (from the substrate stock). The reactions were started by adding coniferyl alcohol (final conc. 50 mM). The vials were incubated for different reaction time (0-24 hours) horizontally at 30°C and 120 rpm. **C:** Enzymatic stability of ACO-03 with various additives at 30°C. Reaction condition: ACO-03 (freeze-dried CFE, 10 mg/mL), bovine liver catalase (10 mg/mL) in tris-HCl buffer (50 mM, pH 8.0) containing $FeSO_4*7H_2O$ (1 mM), sodium ascorbate (1 mM). The samples were incubated horizontally at 30 °C and 120 rpm then the reaction was started by adding coniferyl alcohol (5 mM). Afterwards the reactions were incubated for 3 hours at 30°C and 120 rpm. Blue circles: standard reaction conditions. Green squares: with DTT (1 mM) addition. Yellow triangles: with fresh iron(II) (1 mM) addition. **D:** Vanillin inhibition effect on ACO-03. Reaction conditions: ACO-03 (freeze-dried CFE, 10 mg/mL), bovine liver catalase (10 mg/mL) were mixed in tris-HCl buffer (50 mM, pH 8.0) containing $FeSO_4*7H_2O$ (1 mM) and sodium ascorbate (1 mM) in presence of vanillin (0-40 mM). Reaction was started by adding coniferyl alcohol (final conc. 5 mM) and incubated overnight at 30 °C and 120 rpm.

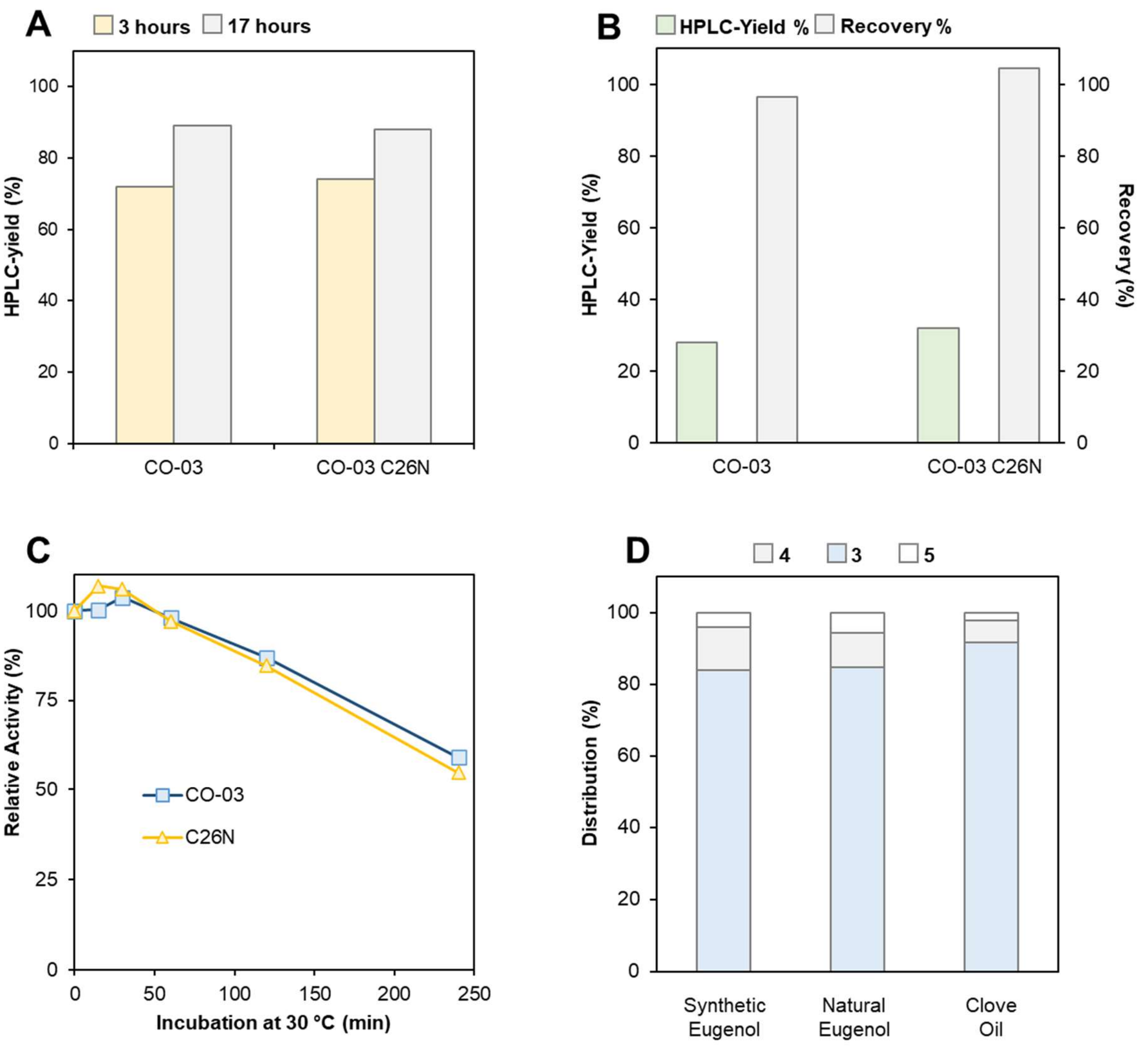

Figure S12. ACO-03 C26N characterization and comparison with ACO-03. A: Biotransformation with 5 mM coniferyl alcohol. B: Biotransformation with 50 mM coniferyl alcohol C: Enzymatic stability at 30°C and pH 8.0. D: Biocascade with different eugenol sources (~ 5 mM) performed with *Rj*EUGO and ACO-03 C26N. Reaction conditions of the biocascade: The reaction mixtures (0.5 mL) were set up in 4 mL glass vials as follows: *Rj*EUGO (freeze-dried CFE, 382 mU/mL), ACO-03 C26N (freeze dried CFE, 21 mU/mL), bovine liver catalase (≥ 100 KU/mL for 0.5 mL reactions, final conc. 10 mg/mL) were mixed in Tris-HCl buffer (50 mM, pH 8.0) containing $FeSO_4$ (1 mM), sodium ascorbate (1 mM), DTT (1 mM), 5% v/v DMSO and eugenol from different sources (5 mM). The vials were incubated for 17 hours horizontally at 30 °C and 120 rpm rotary shaker (25 mm). Every 2 h till 7 h from starting the reaction, $FeSO_4$ (1 mM) was added. The reported values are an average of two replicates.

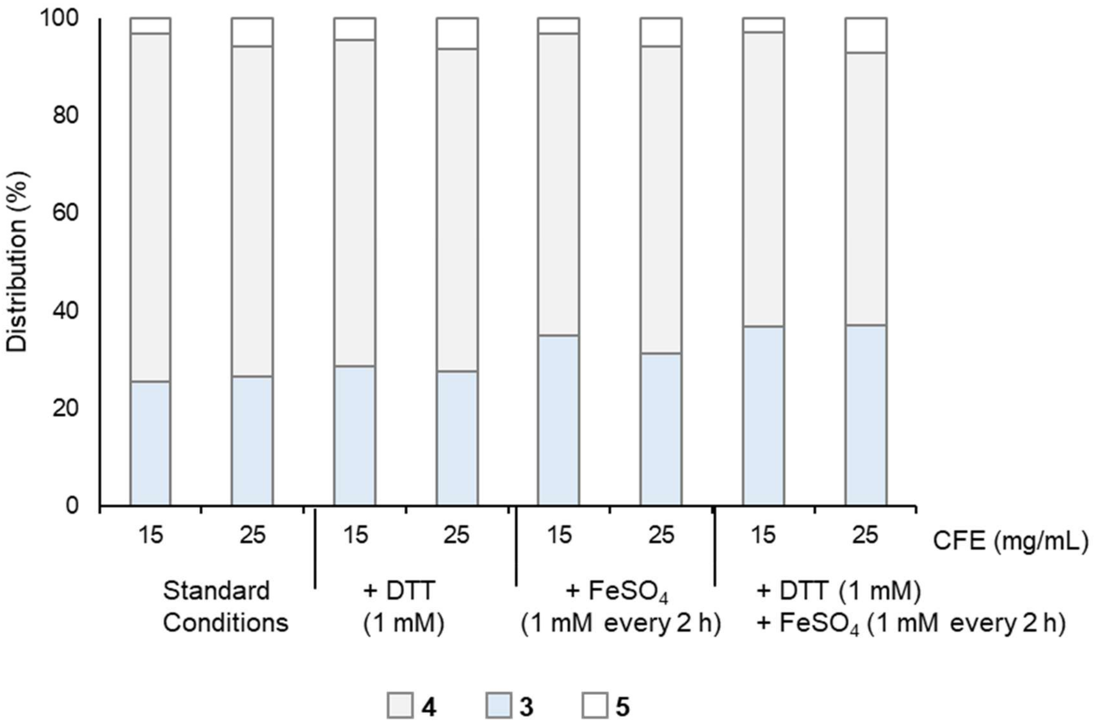

Figure S13. Effect of fresh iron addition on the biocatalytic cleavage of coniferyl alcohol. Reaction conditions: ACO-03 C26N (freeze-dried CFE) and bovine liver catalase (10 mg/mL) were mixed in reaction buffer pH 8, plus the respective additives. The reaction was initiated with the addition of coniferyl alcohol (0.025 mL from 100 mM stock in DMSO, final conc. 50 mM). Incubation for 17 hours at 30°C and 120 rpm. The results are an average of two replicates.

Table S6. 1-pot-2-steps cascade with 50 mM eugenol **1**.

**pH**	**Conv. of eugenol 1 [%]**	**Vanillin 3**	
		**[mM]**	**[%]**
8.0	49	15	30
9.0	37	12	24

[a] Reaction condition: The reaction mixtures (500 µL) were set up in 4 mL glass vials as follows: *Rj*EUGO (final conc. 10 mg freeze dried CFE/mL), ACO-03 C26N (final conc. 15 mg freeze dried CFE/mL), bovine liver catalase (≥100 kU/mL for 0.5 mL reactions, final conc. 10 mg/mL) were mixed in Tris-HCl buffer (50 mM, pH 8.0) or Glycine-NaOH buffer (50 mM, pH 9.0) containing FeSO4*7H2O and sodium ascorbate (final conc. 1 mM), DTT (final conc. 1 mM) and eugenol (final conc. 50 mM). The vials were incubated for 17 hours horizontally at 30 °C and 120 rpm rotary shaker. The cascade was performed in one-pot two-step fashion, meaning first *Rj*EUGO, eugenol and catalase were added to the buffer containing FeSO4*7H2O and sodium ascorbate (final conc. 1 mM). After 17 h, DTT, catalase, ACO-03 C26N were added and reaction was continued another 17 h. Every 2 hours additional $FeSO_4$ was added (from 7 hours from starting the reaction). Due to volume increment, the concentration was reduced from 50 mM to 32.5 mM at the end of the reaction. The experiment was conducted in duplicate.

CHROMATOGRAMS - References

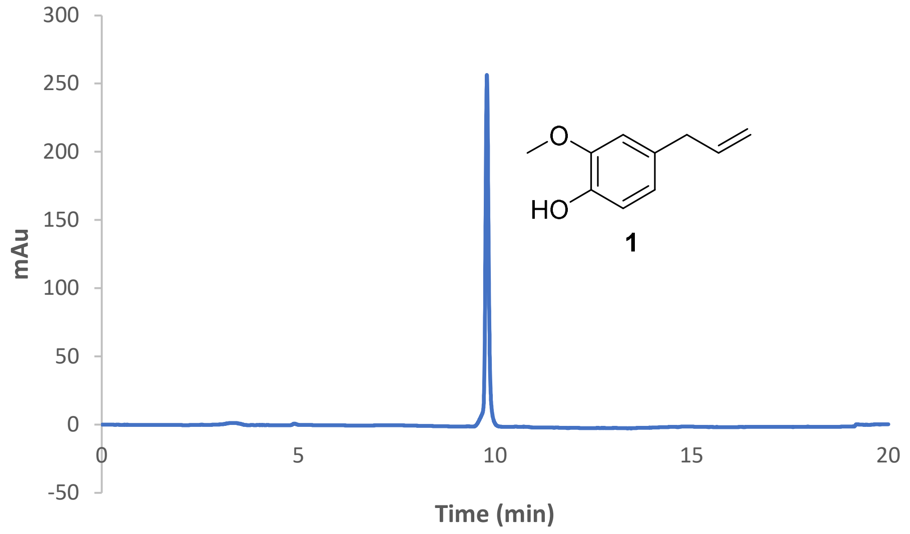

Figure S14. HPLC chromatogram of eugenol **1** at 280 nm. Retention time: 9.8 min.

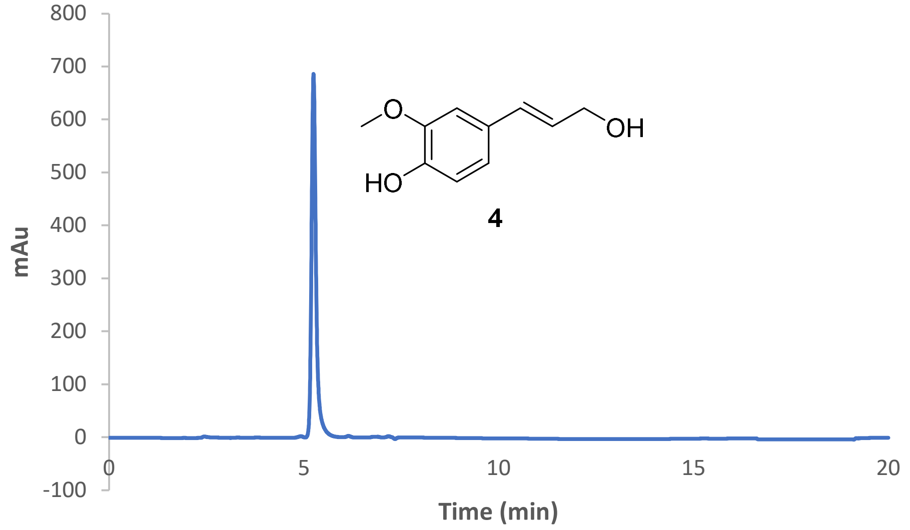

Figure S15. HPLC chromatogram of coniferyl alcohol **4** at 280 nm. Retention time: 5.3 min.

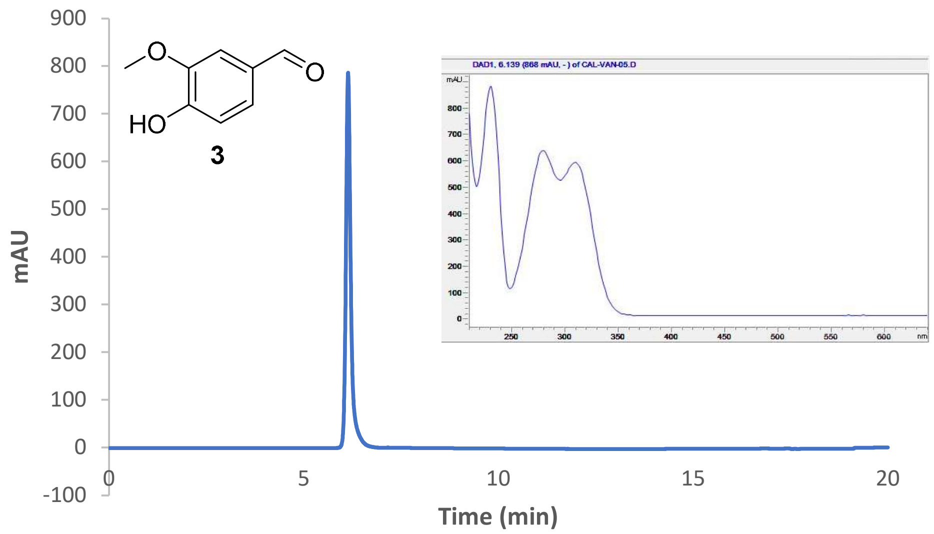

Figure S16. HPLC chromatogram of vanillin **3** at 280 nm. Retention time: 6.1 min. The insert shows the UV-Vis spectrum extracted from the chromatogram.

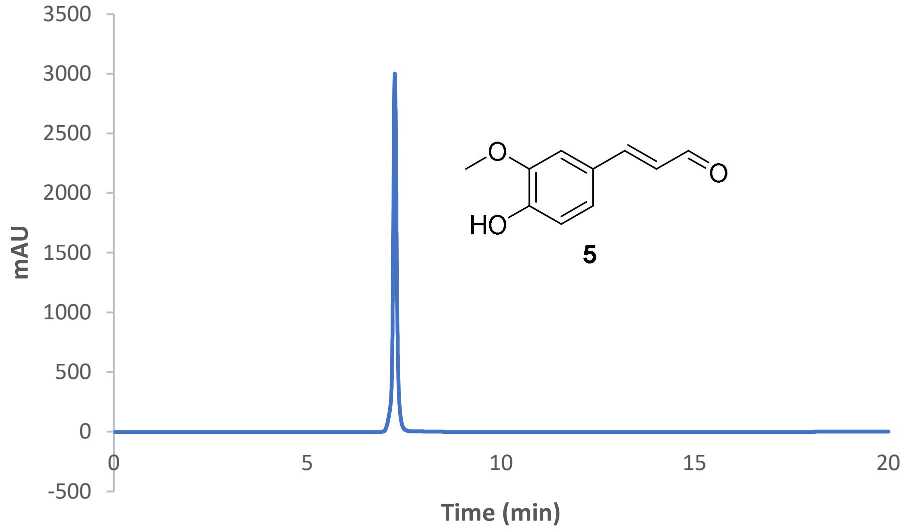

Figure S17. HPLC chromatogram of coniferyl aldehyde **5** at 340 nm. Retention time: 7.2 min.

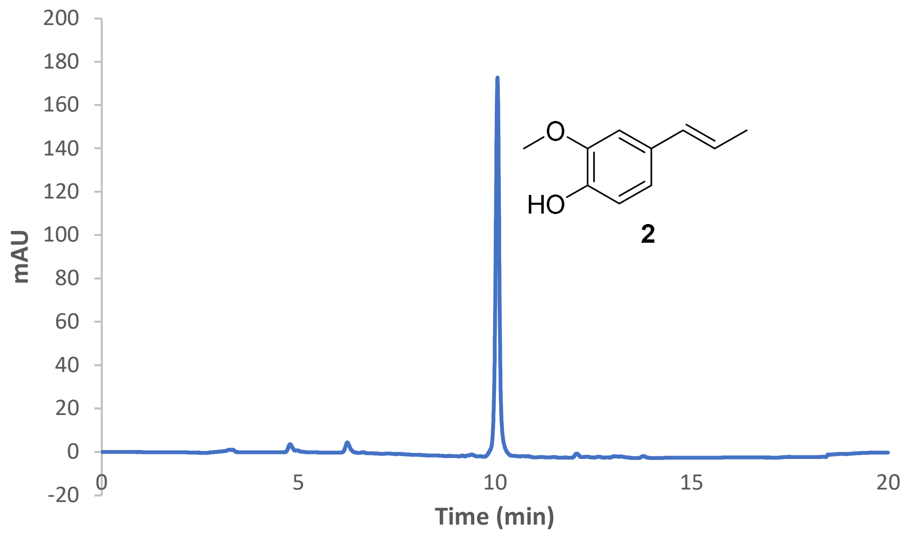

Figure S18. HPLC chromatogram of isoeugenol **2** at 280 nm. Retention time: 10.1 min.

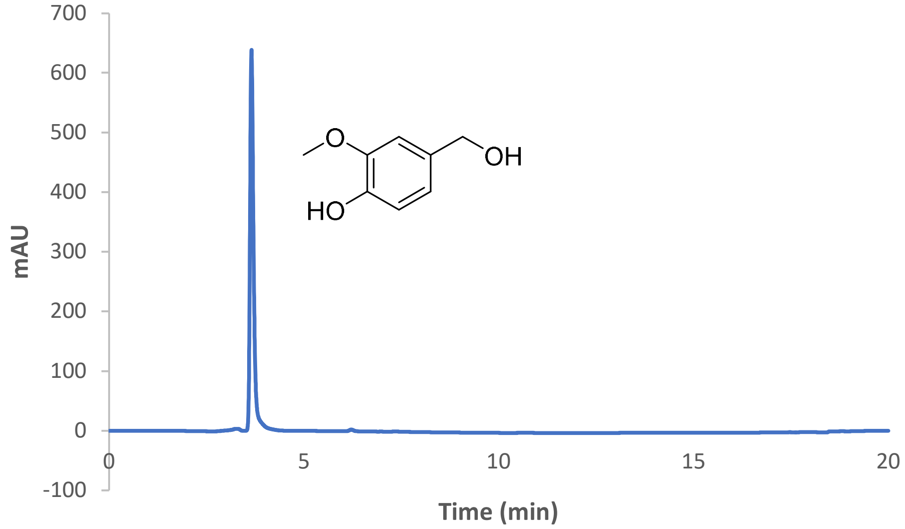

Figure S19. HPLC chromatogram of vanillyl alcohol at 280 nm. Retention time: 3.7 min.

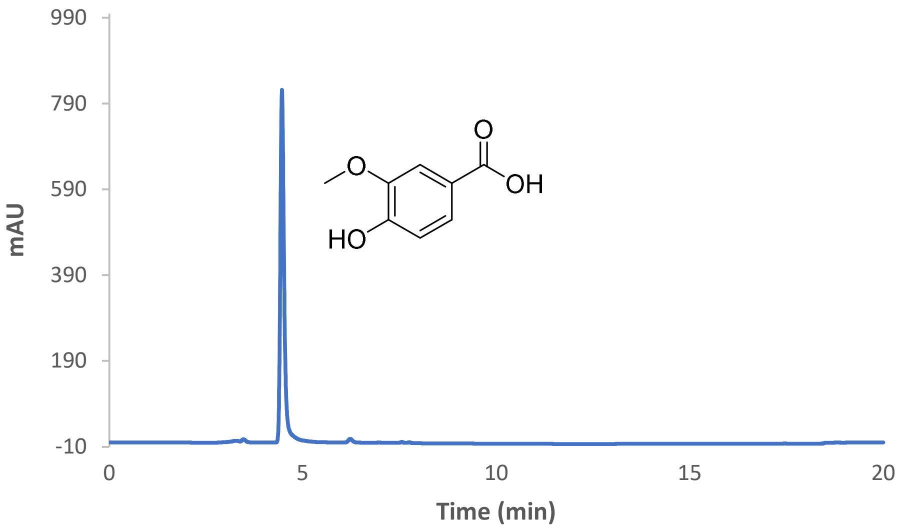

Figure S20. HPLC chromatogram of vanillyl acid at 280 nm. Retention time: 4.5 min.

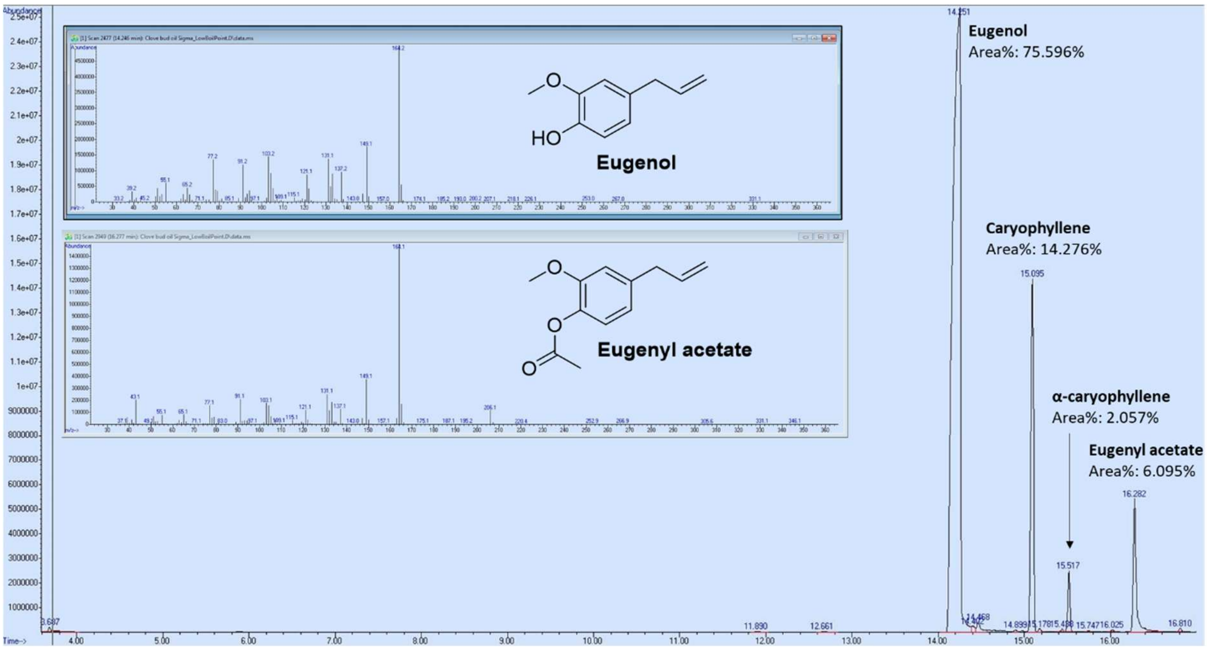

Figure S21. GC-MS analysis of clove oil.

CHROMATOGRAMS – Cascade and controls at 0.5 mL scale

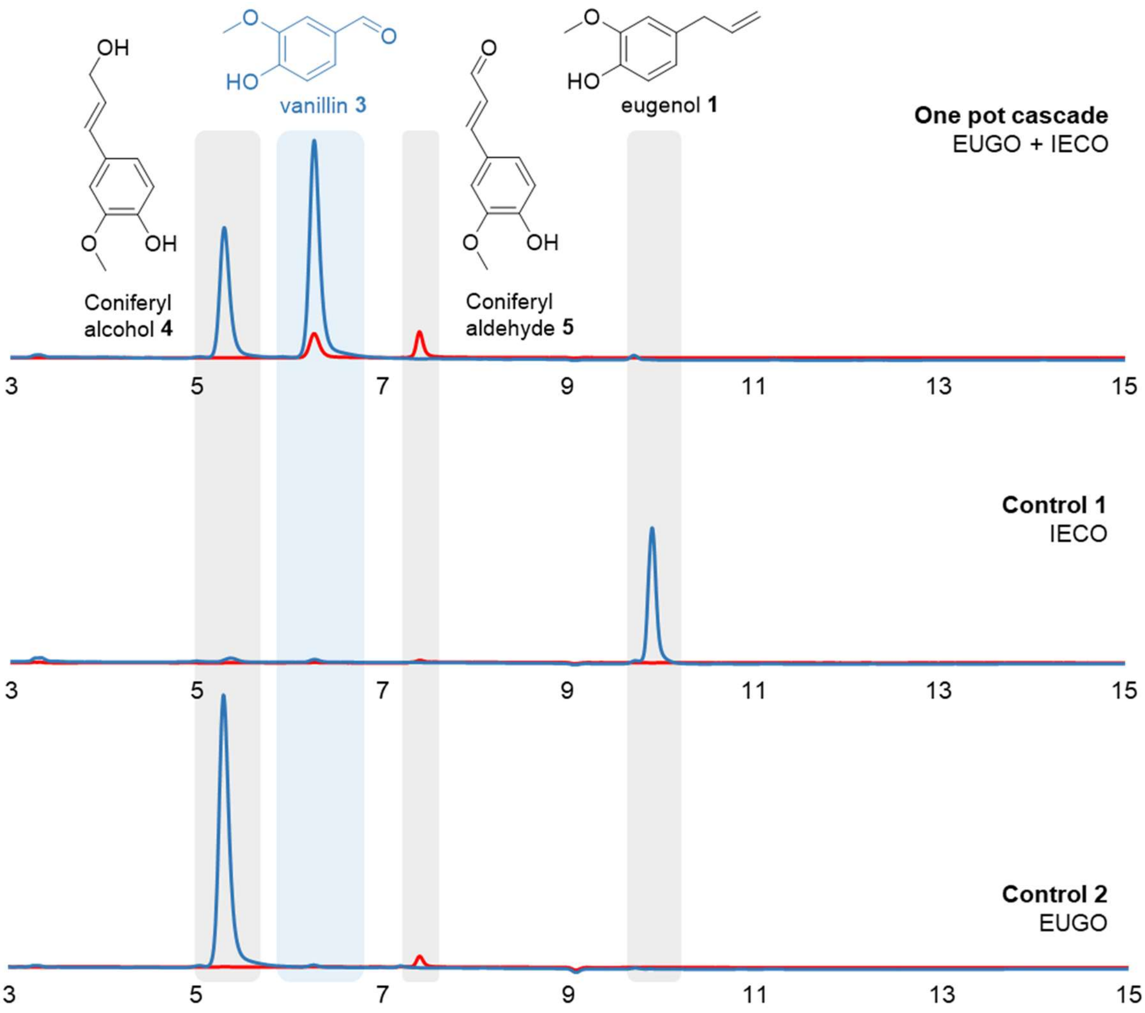

Figure S22. HPLC chromatograms of the enzymatic conversion of eugenol **1** to vanillin **3** after 24 hours. Blue line: 280 nm. Read line: 340 nm. Reaction conditions: Glycine-NaOH buffer (50 mM, pH 9.0), sodium ascorbate (1 mM), $FeSO_4$ (1 mM), ethanol 5 vol%, bovine liver catalase (Sigma C40, 10 mg/mL = ≥100 kU/mL), eugenol (5 mM), *Rj*EUGO (CFE 1 mg/mL), *Pn*IECO (CFE 4 mg/mL). Each reaction mixture (0.5 mL) was set up in 1.5 mL glass vials and incubated at 30 °C and 120 rpm. Control 1 was performed in Tris-HCl (50 mM, pH 8.0).

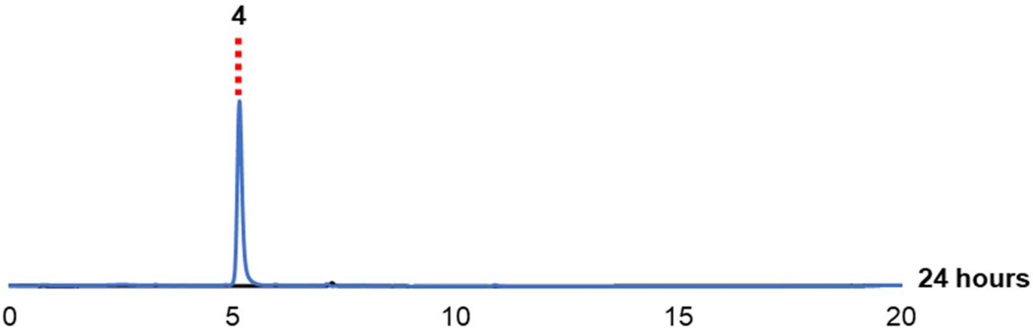

Figure S23. HPLC chromatogram after 24 hours incubation of coniferyl alcohol **4** in the reaction buffer. Blue Line: 280 nm. Black line: 340 nm. Coniferyl alcohol (5 mM) was mixed in Tris-HCl (50 mM, pH 8.0) together with Fe(II) salt (5 mM), sodium ascorbate (1 mM), 5 % v/v ethanol and bovine liver catalase (10 mg/mL). The mixture was incubated at 30 C° and 120 rpm (rotary shaker) for 4 hours.

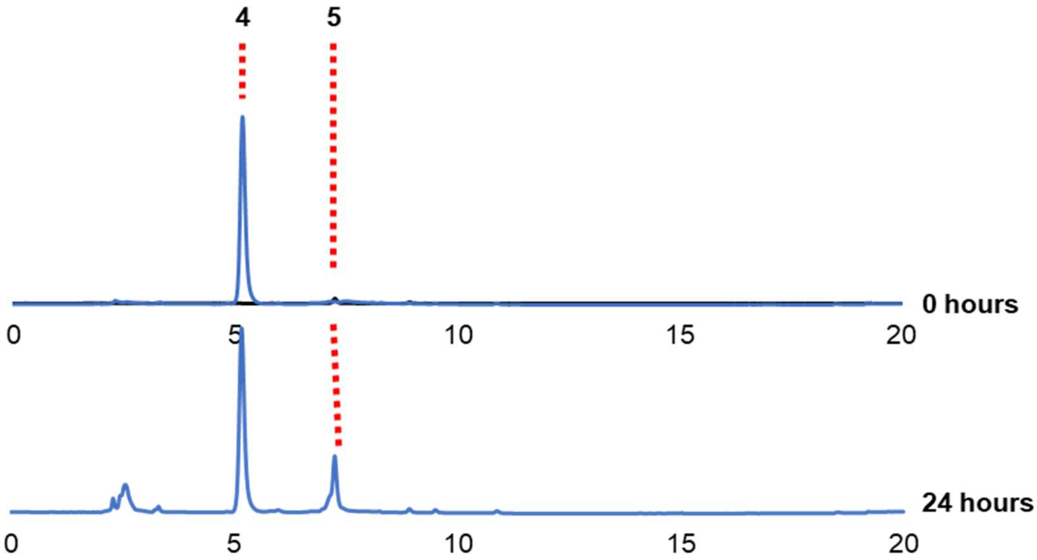

Figure S24. HPLC chromatogram before and after 24 hours incubation of coniferyl alcohol **4** with empty *E. coli* BL21 (DE3) cell free extract. Blue Line: 280 nm. Black line: 340 nm. Coniferyl alcohol (5 mM) was mixed in Glycine-NaOH (50 mM, pH 9.0) together with $FeSO_4$ (1 mM), sodium ascorbate (1 mM), 5 % v/v ethanol, *E. coli* BL21 (DE3) (freeze-dried CFE 6 mg/mL) and bovine liver catalase (10 mg/mL). The control reaction was incubated at 30C°C and 120 rpm (rotary shaker) for 24 hours.

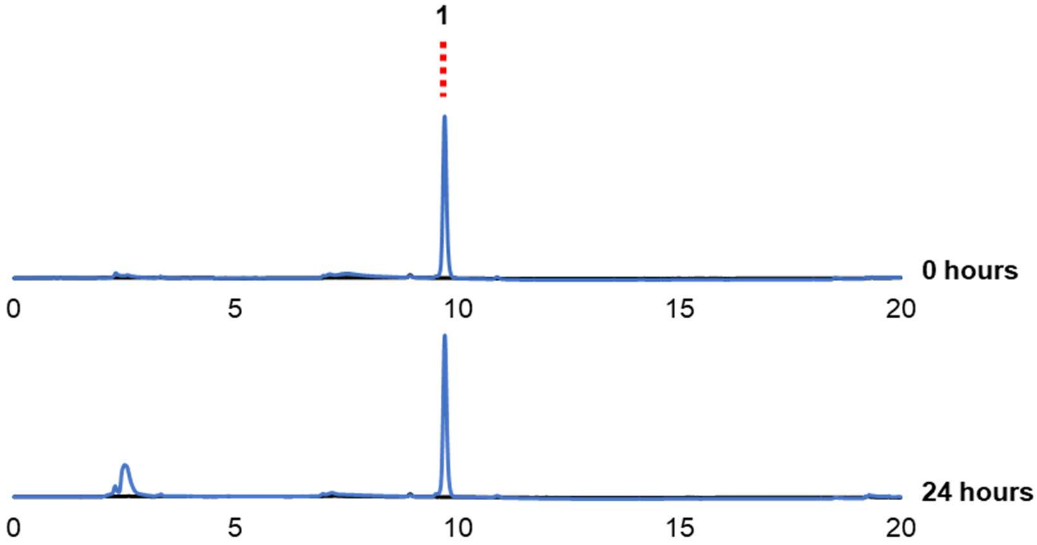

Figure S25. Control-03. HPLC chromatogram before and after 24 hours incubation of eugenol **1** with empty *E. coli* BL21 (DE3) cell free extract. Blue Line: 280 nm. Black line: 340 nm. Eugenol (5 mM) was mixed in Glycine-NaOH (50 mM, pH 9.0) together with $FeSO_4$ (1 mM), sodium ascorbate (1 mM), 5 % v/v ethanol, *E. coli* BL21 (DE3) (freeze-dried CFE 6 mg/mL) and bovine liver catalase (10 mg/mL). The control reaction was incubated at 30C°C and 120 rpm (rotary shaker) for 24 hours. Comparable results were obtained in Tris-HCl pH 8.0.

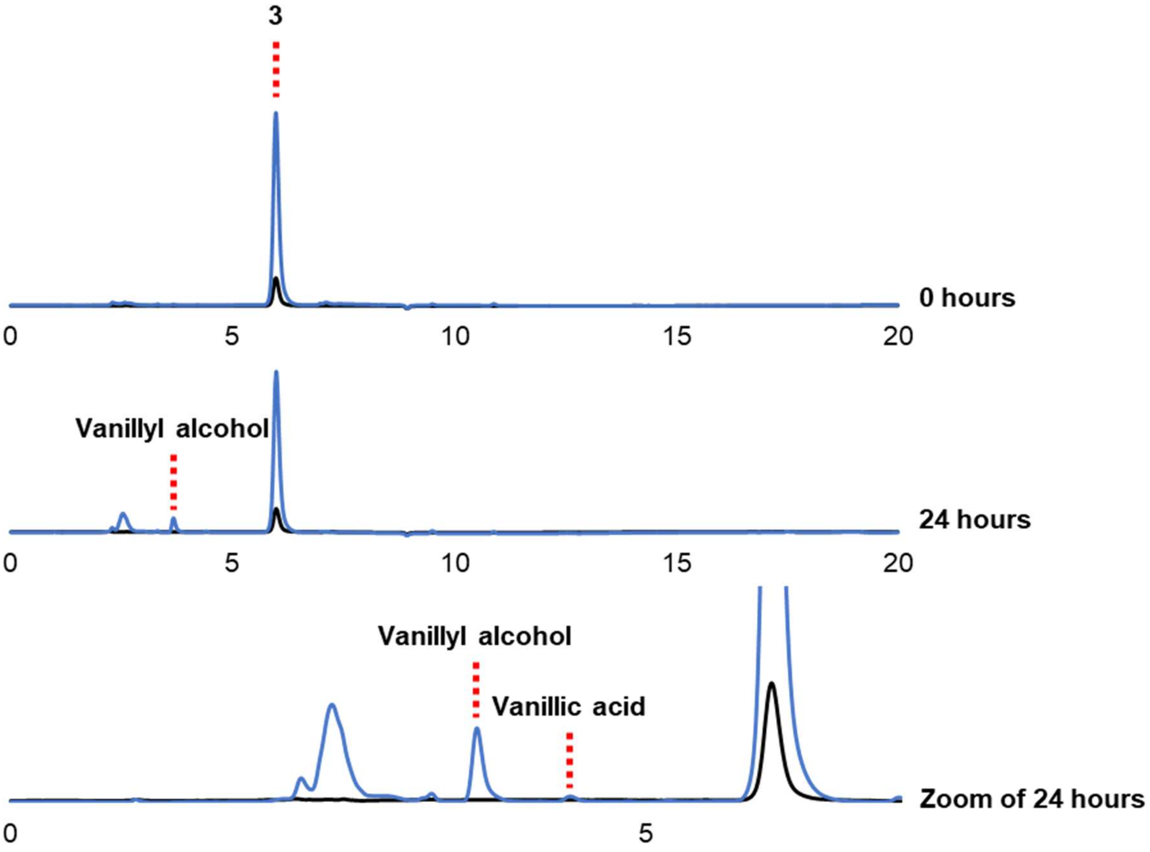

Figure S26. HPLC chromatogram before and after 24 hours incubation of vanillin **3** with empty *E. coli* BL21 (DE3) cell free extract. Blue Line: 280 nm. Black line: 340 nm. Vanillin (5 mM) was mixed in Glycine-NaOH (50 mM, pH 9.0) together with $FeSO_4$ (1 mM), sodium ascorbate (1 mM), 5 % v/v ethanol, *E. coli* BL21 (DE3) (freeze-dried CFE 6 mg/mL) and bovine liver catalase (10 mg/mL). The control reaction was incubated at 30C°C and 120 rpm (rotary shaker) for 24 hours. Comparable results were obtained in Tris-HCl pH 8.0.

CHROMATOGRAMS – Reactions at 3 mL scale and 50 mM substrate loading

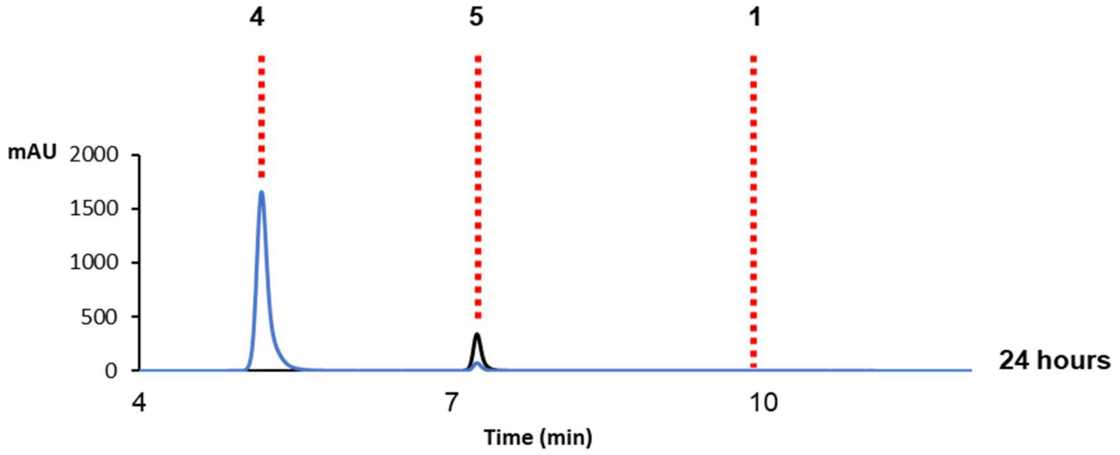

Figure S27. Extract of the HPLC chromatogram of the reaction step 1 at 50 mM substrate loading and 3 mL scale. Blue line: 280 nm. Black Line: 340 nm.

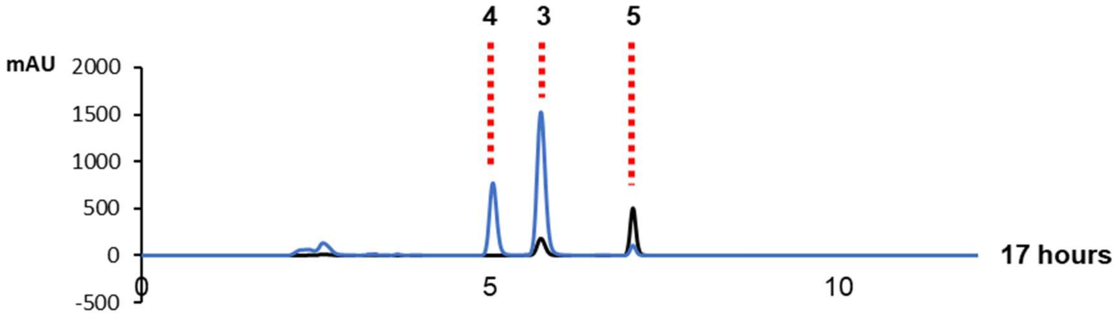

Figure S28. Extract of the HPLC chromatogram of the reaction step 2 at 50 mM substrate loading and 3 mL scale (continuous oxygen bubbling for 7 hours). Blue line: 280 nm. Black Line: 340 nm.

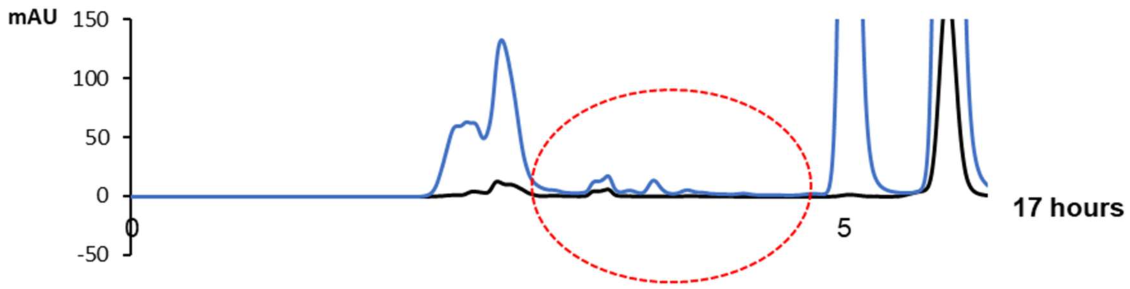

Figure S29. Zoom of Figure S28. Vanillyl alcohol was not detect at the end of the reaction. Blue line: 280 nm. Black Line: 340 nm.

CALIBRATION CURVES

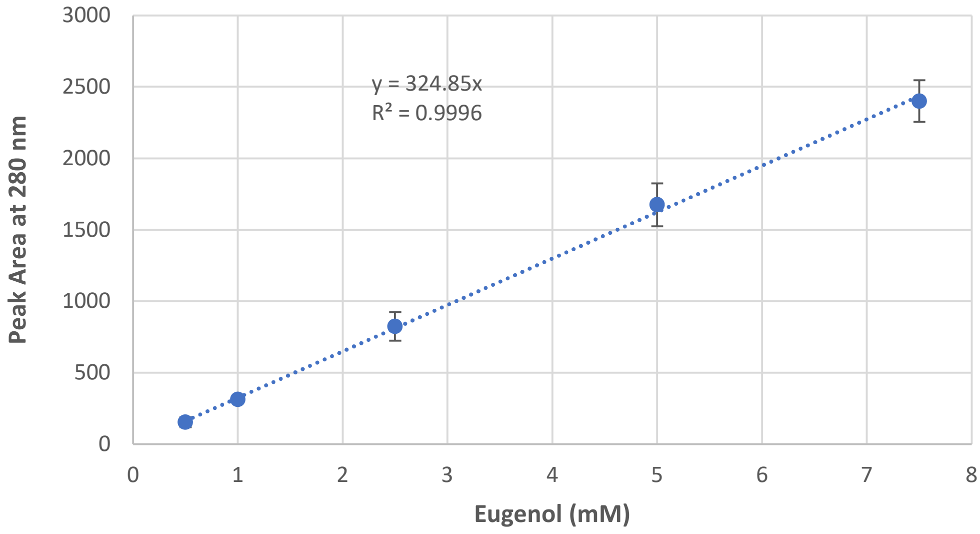

Figure S30. Calibration curve of eugenol **1** (Rt 9.8 min). Eugenol was detected at 280 nm, using 360 nm as reference wavelength. Each standard was treated as the reaction samples before HPLC analysis. Error bars indicate the standard deviation of at least three independent experiments.

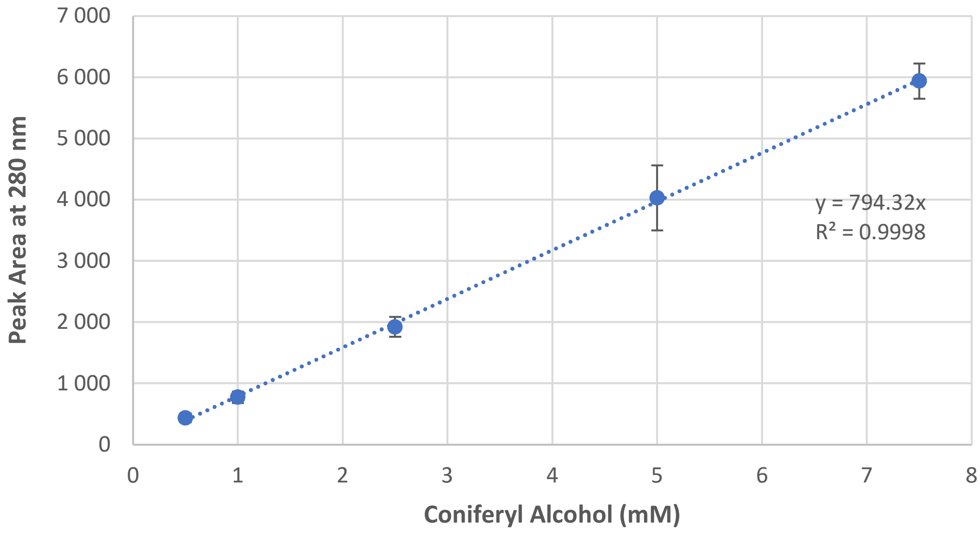

Figure S31. Calibration curve of coniferyl alcohol **4** (Rt 5.2 min). Coniferyl alcohol was detected at 280 nm, using 360 nm as reference wavelength. Each standard was treated as the reaction samples before HPLC analysis. Error bars indicate the standard deviation of at least three independent experiments.

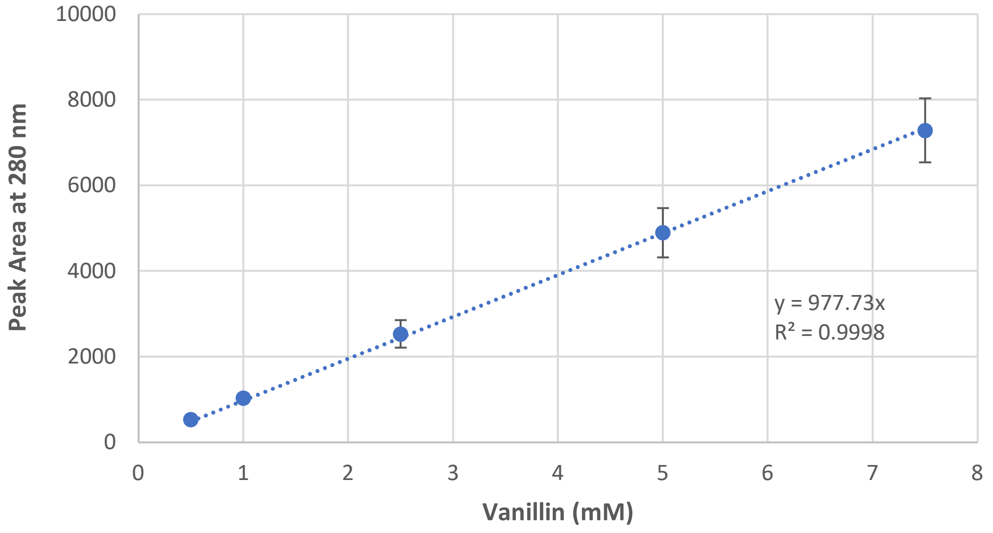

Figure S32. Calibration curve of vanillin **3** (Rt 6.1 min). Vanillin was detected at 280 nm, using 360 nm as reference wavelength. Each standard was treated as the reaction samples before HPLC analysis. Error bars indicate the standard deviation of at least three independent experiments.

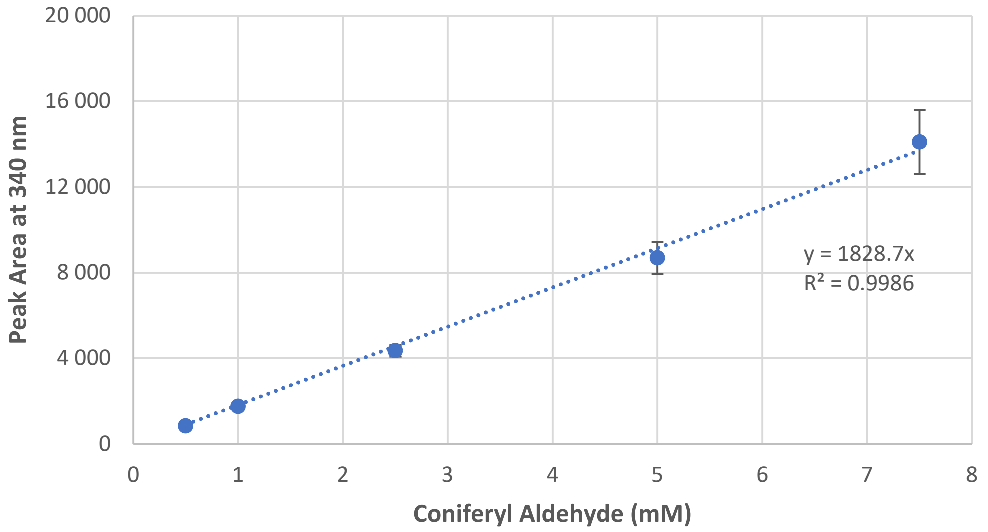

Figure S33. Calibration curve of coniferyl aldehyde **5** (Rt 7.3 min). Coniferyl aldehyde was detected at 340 nm, using 395 nm as reference wavelength. Each standard was treated as the reaction samples before HPLC analysis. Error bars indicate the standard deviation of at least three independent experiments.

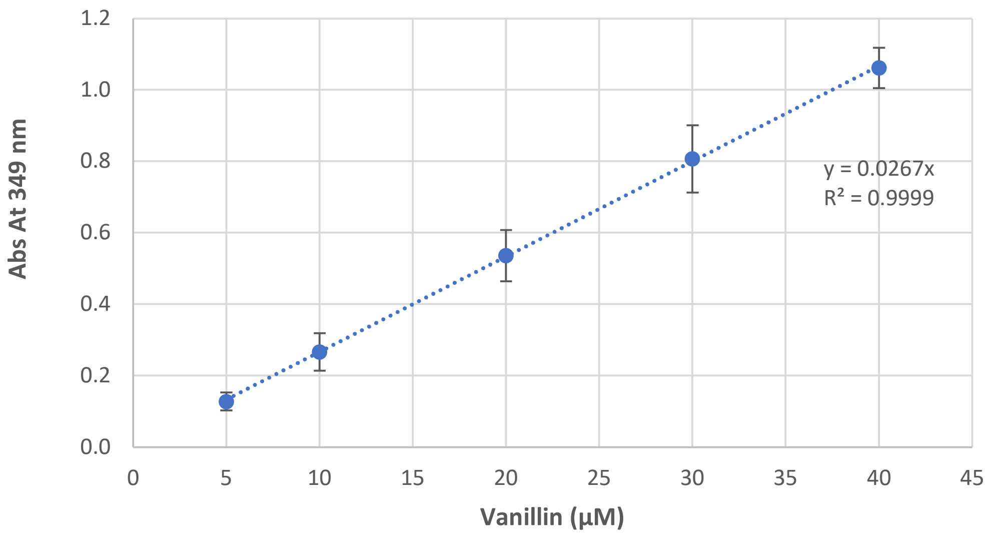

Figure S34. Determination of vanillin extinction coefficient at 349 nm (26.7 $mM^{-1}cm^{-1}$). Error bars indicate the standard deviation of three independent experiments.

NMRs

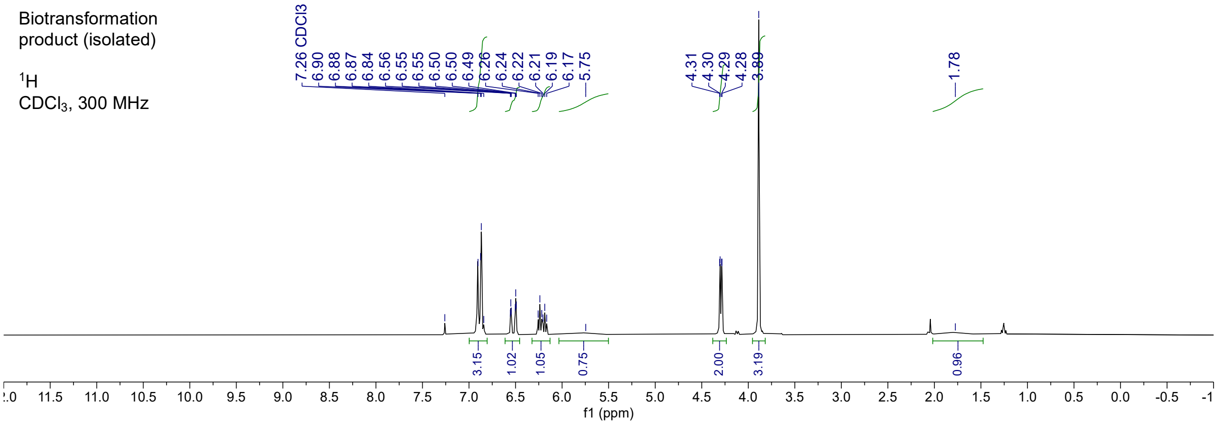

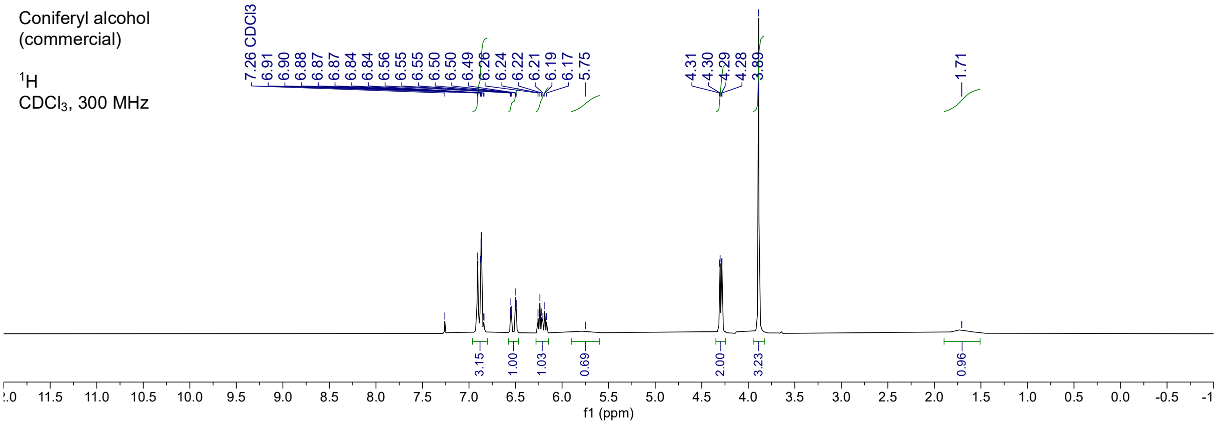

Figure S35. ^{1}H NMR of isolated coniferyl alcohol (top) with the commercial standard (bottom).

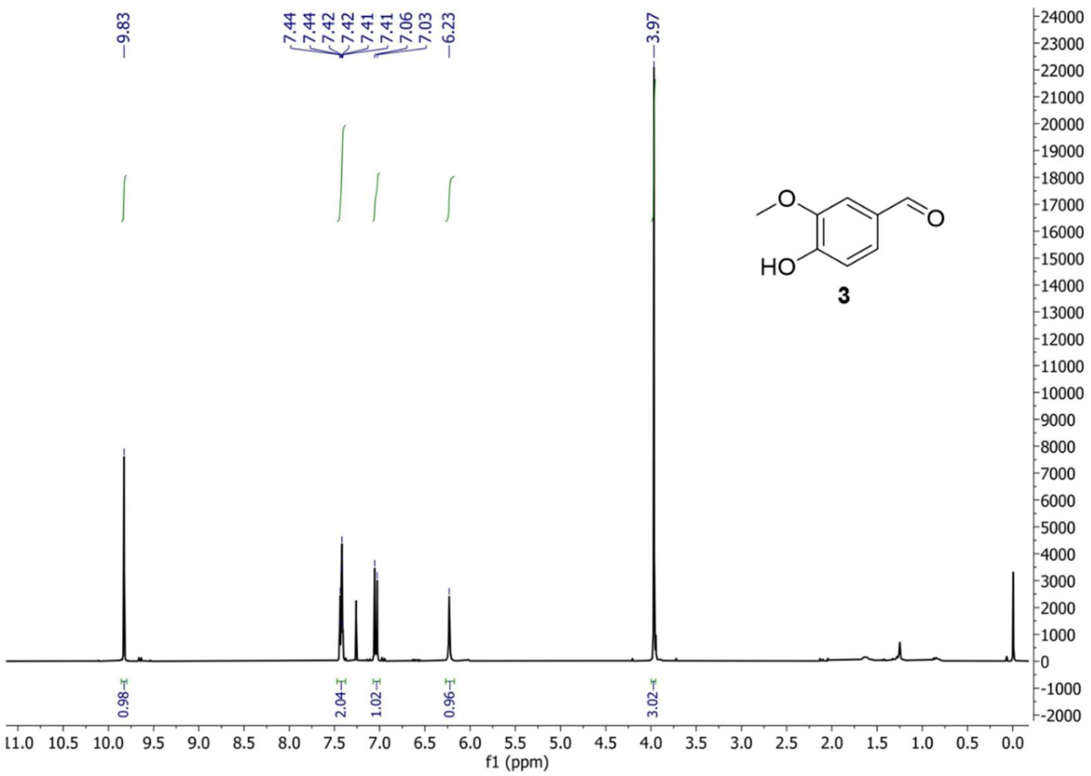

Figure S36. ^{1}H NMR of isolated vanillin **3**.

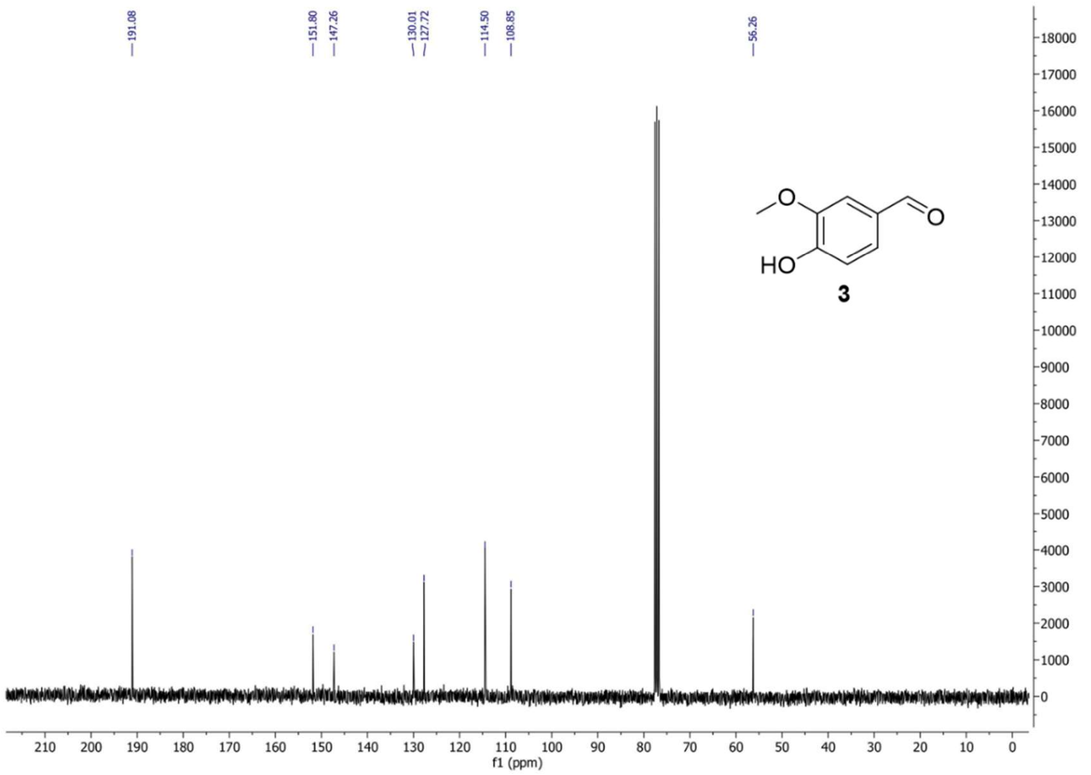

Figure S37. ^{13}C NMR of isolated vanillin **3**.

LIST OF PROTEIN SEQUENCES

(Tags are underlined)

1. Eugenol oxidase from *Rhodococcus Jostii*

MTRTLPPGVSDERFDAALQRFRDVVGDKWVLSTADELEAFRDPYPVGAAEANLPSAVVSPESTEQVQDIVRIANEYGIPLSPV
STGKNNGYGGAAPRLSGSVIVKTGERMNRILEVNEKYGYALLEPGVTYFDLYEYLQSHDSGLMLDCPDLGWGSVVGNTLDRGV
GYTPYGDHFMWQTGLEVVLPQGEVMRTGMGALPGSDAWQLFPYGFGPFPDGMFTQSNLGIVTKMGIALMQRPPASQSFLITFD
KEEDLEQIVDIMLPLRINMAPLQNVPVLRNIFMDAAAVSKRTEWFDGDGPMPAEAIERMKKDLDLGFWNFYGTLYGPPPLIEM
YYGMIKEAFGKIPGARFFTHEERDDRGGHVLQDRHKINNGIPSLDELQLLDWVPNGGHIGFSPVSAPDGREAMKQFEMVRNRA
NEYNKDYAAQFIIGLREMHHVCLFIYDTAIPEAREEILQMTKVLVREAAEAGYGEYRTHNALMDDVMATFNWGDGALLKFHEK
IKDALDPNGIIAPGKSGIWSQRFRGQNLHHHHHH

2. Isoeugenol cleavage oxygenase from *Pseudomonas sp.*

MASWSHPQFEKGADDDDKVPDPMFPTRIEANVFDLEIEGEIPRAINGSFYRNTPEPQVTTQPFHTFIDGDGMASAFHFEDGHV
DFISRWVCTPRLEADRSARKSLFGMYRNPFTDDPSVEGIDRTVANTSIITHHGKVLAAKEDGLPYELDPQTLETRGRYDYKGQ
VTSHTHTAHPKFDPQTGEMLLFGSAAKGEATLDMAYYIVDHYGKVTHETWFKQPYGAFMHDFAVTRNWSIFPIMPATNSLERL
KARQPIYMWEPELGSYIGVLPRRGQGKDIRWFRAPALWVFHVVNAWEEGNRILIDLMESEILPFPFPNSQNLPFDPSKAVPRL
TRWEIDLNSGNDEMKRTQLHEYFAEMPIMDFRFALQDHRYAYMGVEDPRRPLAHQQAEKIFAYNSLGVWENHRKEYDLWFTGN
MSAAQEPAFVPRSPDAPEGDGYLLSVVGRLDEDRSDLVILDTQCLAAGPVATVKLPFRLRAALHGCWQSKN

3. Isoeugenol cleavage oxygenase from *Pseudomonas putida*

MASWSHPQFEKGADDDDKVPDPMATFDRNDPQLAGTMFPTRIEANVFDLEIEGEIPRAINGSFFRNTPEPQVTTQPFHTFIDG
DGLASAFHFEDGQVDFVSRWVCTPRFEAERSARKSLFGMYRNPFTDDPSVEGIDRTVANTSIITHHGKVLAAKEDGLPYELDP
QTLETRGRYDYKGQVTSHTHTAHPKFDPQTGEMLLFGSAAKGERTLDMAYYIVDRYGKVTHETWFKQPYGAFMHDFAVTRNWS
IFPIMPATNSLERLKAKQPIYMWEPERGSYIGVLPRRGQGKDIRWFRAPALWVFHVVNAWEEGNRILIDLMESEILPFPFPNS
QNLPFDPSKAVPRLTRWEIDLNSGNDEMKRTQLHEYFAEMPIMDFRFALQDHRYAYMGVDDPRRPLAHQQAEKIFAYNSLGVW
DNHRKDYELWFTGKMSAAQEPAFVPRSPDAPEGDGYLLSVVGRLDEDRSDLVILDTQCLAAGPVATVKLPFRLRAALHGCWQS
KN

4. Isoeugenol cleavage oxygenase from *Pseudomonas nitroreducens*

MASWSHPQFEKGADDDDKVPDPMARLNRNDPQLVGTLLPTRIEADLFDLEVDGEIPKSINGTFYRNTPEPQVTPQKFHTFIDG
DGMASAFHFEDGHVDFISRWVKTARFTAERLARKSLFGMYRNPYTDDTSVKGLDRTVANTSIISHHGKVLAVKEDGLPYELDP
RTLETRGRFDYDGQVTSQTHTAHPKYDPETGDLLFFGSAAKGEATPDMAYYIVDKHGKVTHETWFEQPYGAFMHDFAITRNWS
IFPIMPATNSLSRLKAKQPIYMWEPELGSYIGVLPRRGQGSQIRWLKAPALWVFHVVNAWEVGTKIYIDLMESEILPFPFPNS
QNQPFAPEKAVPRLTRWEIDLDSSSDEIKRTRLHDFFAEMPIMDFRFALQCNRYGFMGVDDPRKPLAHQQAEKIFAYNSLGIW
DNHRGDYDLWYSGEASAAQEPAFVPRSPTAAEGDGYLLTVVGRLDENRSDLVILDTQDIQSGPVATIKLPFRLRAALHGCWVP
RP

5. Lignostillbene dioxygenase from *Pseudomonas brassicacerarum*

MASWSHPQFEKGADDDDKVPDPMSIPFPQTPEFSGALYKPSRIEAEVFDLEIEGVLPASIHGTFYQVAPDPQYPPMLGTDIFF
NGDGMVSGFHFANGKVSLRRRYVQTDRLLAQRREGRSLNGVYRNAFTNDSLAAKNNTTANTSVIPHNGVLLALKEDALPWAMD
LETLETLGEWTFDGQIKSATFTAHPKLDPATGNLLAFSYEAKGDGTPDLVYFELSPDGKLLHEIWFQAPYAAMVHDFAATERY
VVFPLIPLTVDVERMKNGGPHFQWQPDLPQLFAVVPRNGRAQDVRWFKGPMDGFQGHTLNAFDEDGKVYVDMPVTGGNIFYFF
PQADGHVPPPETLAACLMRWTFDLNSGRDEVEPQPLTDYPCEFPRCDDRYIGRQYAHGFLLAFDPERPYNPANGPIPFQFFNL
LVHLNLKTGLSDAWFPGDSGCFQEPIFIPRSADAEEADGYVVALLNLIAEERSELVVLDSRDMASGPIARIRIPFRMRMSLHG
CWAPG

6. Aromatic dioxygenase from *Thermothelomyces thermophilus* - ACO-01

MASWSHPQFEKGADDDDKVPDPMAHIHDLAPEVSNYSSGRLTPPTPVRFPRTPVFASMNKPCRFEGDVFDLEVSGAIPPDIDG
TFFRVQPDHRFPPLFEDDIHFNGDGSVTAIRISGGHADLRQRYVRTERYLLETRARRSLFGRYRNPWTDNESVRGVIRTASNT
NVVFWRGALLAMKEDGPPFAMDPVTLETLGRYDFEGQILSPTFTAHPKIDPDTGEMVCFAYEAGGDGSDCSVDVAVWTVDADG
KKVEECWYKAPFAGMIHDCGITKNWVVLPLTPIKMDLERMKRGGNKFAWDPSEDQWYGVVPRRGAKSDDIIWFRADNGFHGHV
AGCYELPSGEIVFDLTVADGNVFFFFPPDDNITPPADGVAKRNRLSSPTVRWIFDPKAKKSAIRTEAAGDADIWVADERVKPA
LTWPTNGEFSRIDDRYVTKPYRHFWQAVVDPTRPYDFEKCGPPAGGLFNCLGHYTWSDQNYHHGHNTGDPSGDGRSNGSAEEA
TAGKFGLQDVYFAGPTMTFQEPTFIPRQGAAEGEGYLIALLNHLDELRNDVVIFEARNLGKGPLAVIHLPLKLKLGLHGNWVD
SREIEAWRRRRAENGDVGPLRVAKEPLPWQKKFAAAAQNGSNGV

7. ACO-02

MASWSHPQFEKGADDDDKVPDPMTRFPQREGASAFEAPMRVEADIAGLEVVQGEVPEHLSGTYYRVVTDRQWPPAVLPDVPTF
NDDGMAMYFRFHGGRVDFRSRYVRTPRYEAEAKAGRALFGAYRNPLTDDPSVAGISRGLGNTNVFYHGGKLYASKEDSAPILL
DPMSLETIGEYTFEGTLTSRTCTAHPKVDPRTGEMVFFGYAAKGETTPDIAYYEADQDGRIIHETWIRAPYSSMVHDWAVTQN
FVVFPIIPMRSEYAWLEARQPHFQWDPDRDVYLGVLPRKGSADQVRWLRGSNRFASHILGAWDDGRHIHIETPVGRSNFFPFF
PDVTGAPYDPVRARGYLSRWTIDMGSDGGTFTEQRLTDVAGEFPRMDDRFETLPYRRGFLALNHVPGEQRPGAGFRWIGAFDT
TGAAPPQIYYPGDDCSVAEPLFVPGGDDAPEGHGYVFVVVGRHQEMRSDLVILDAQRLDAPPVATVALPIRIRRGLHGNWVSS
TELAARTA

8. ACO-03

MASWSHPQFEKGADDDDKVPDPMSLFNRNHPVFSGGNAPVRIETDAICLETDGALPDELKGSYFRLTADPQFPPRLRPVLVEA
DGHVGAFHFRDNGQVDYVGKWVRTERFLRERAARRALFGTYRNRFTDEESVLDSDRTTANTAFMFHHHKFFALKEDGLPYQLD
PETLDTIGRYDFGGTVSAKSLSAHPKVDSRTGELITHSGQAKGEGTPDIAYYVFDKNGTKTVERWFEAPYSSIVHDIAITQDW
VVLPIMPAVVEEERLRAGGATYWWKPELGNHIAVFRRDGTGDVRWFRTKATYAFHVVNSYQDGDRLIIDVMDAEEFPMWWPRP
EQVAALRSGEIKRDKFVAQLTRWTIDLAGDSDDIERELLHPWEAEMPRIDDRFAGQPYRYAVYGVDDPSFPLAHGLAELGVNH
NSVGWWDHQTRTLTSWYTGPNSSVGEPVFTPRSPDAPEGDGFILAVVQRLAEQRSELVVIDTRDVAAGPIASVHAPHRLKNAI
HNLWIDQEQLNEGGEKA

9. ACO-04

MASWSHPQFEKGADDDDKVPDPMIHVASPDDRGSTVHPKETHVIRFPVTDDPFDAPMRVEADITDLECTQGAVPEHLDGTYYR
VVVDRHYPSMVDNDLALFNSDGMAMSFTFSKGRVDFKTRYVRTPRFVAEEKAGKSLFGAYRNASTDDPSVAGIRRGLANTNVF
FHGGRLYASKEDSPPILLDPFSLETLGEYDFQGALTSETSTAHPKVDPRTGEMVFFGYGAKGEATPDIAYYEADASGQIIHEA
WITAPYSCMIHDFAVTQNYVIFPIVPLRSSTEWIAQGNNQYFWDPDEDVYLGVLPRKGSGKDLRWFRGSNRFASHIMGAFDDG
RHIYIDTPVAETSYFPWFPDISGASYDPDRSKTHLSRWTIDLADSTGGFTERRLMTAAGEFPRMDDRQETLAYDWGVLAINDL
PGSPKPGPGFRWIGAVDLPSGSTKFYDPGPNATVGEPIFVPGQADAKPGHGYVFVVVARRDLMRSDLVILDAQNLDKPPVATL
SLPIRIKSGLHGNWVGADELAGRTA

10. ACO-05

MASWSHPQFEKGADDDDKVPDPMVTRFPDQPGFTGFDAPMRVEADLMDVEVSQGAIPENLEGTYYRVVADFQWPPKVDGDFFF
NGDGMVMSFRFSGGHADFKSRYVRTPRFVAQAKERRALFGAYRNPYTDDPSVAGMSRGLANTNVFWHGGKLLSSKEDSPPMMI
DPDTLETIGEHTFDGALTSQTSTAHPKIDPRTGEMVFFGFAAKGETTPDIAYYEADSRGVVIHETWLRAPYSSMVHDFAVTQN
FVVFPIIPLTSDLERLKAGGPHYAWDPHKDVYLGVLPRKGNGADIRWYRGSNRFASHIMGAHDDGRHIFIDTPVSESNFFPFF
PDLSGAGYEAEKAKGYLSRWTIDTMGESNGFTETKIARYPGEFPRMDDRRETLPYNWGVLALSDVPEQESPGRGFRWISSIDL
NTGHSQIYYPGHDCSVAEPLFVPAHDNAEEREGYVFVVIGRHEEMRSDLVILDAAHLDAPPVATLKMPLRIRSGLHGNWVSAT
ELARERTA

11. ACO-06

MASWSHPQFEKGADDDDKVPDPMNIRFPNNDFTSFEAPMRAEIDLKGLEVIQGEVPRQLSGGFFRLIGDRKWPAFVENDIFML
GEDGMASCFYFKDGQVDFKMRYVRTPRFVAEEKAGRALFGHYRNPFSDDPSVAGLSRGTANVTVLVHAGKMLALKEDSPAIEL
DPFTLETIGEYDWGGVVTAKAITAHPKIDPKTGELVFFGYCAAGPATRDIAYYEADAEGRIIHETWFEAPYTCMVHDIMVTEN
YVVFPITPLRHELEWIERNEPAFKWDPSEDVYLGVLPRKGKGEQVRWFKGPNQCHGHTVGAFDDGRFIYADNTFSKRSFFHFF
PNTDGSEFNPADAMPFIKRLVIDMESNDDSFTEQQMFGYPCELPRIDTRFETMPYNFVTMNLLDVPEQERIGMGFQWVATMDI
NGQKPAKIYYAGHDCSIGEPQFVPRSDDAAEGDGYILTVVGKHREMRSELLILDAGDITAPPIATVALPFRLRSTGHGYWYGT
RQLRGETNIIGAW

12. ACO-07

MASWSHPQFEKGADDDDKVPDPMVWFPPPKDPFDAPMRVEADIRGVQVTQGEVPEHLDGTYYRCVSDRQWPSYVENDLELFNS
DGMIISFRFHKGRVDFKSRYVRTPRFVAEEKAGRALFGAYRNPFDDDPSVAGISRGLANTNVFFHGGKLYASKEDSPPILIDP
DTLETIGEYDFEGALTSRTATAHPKFDPVTGEMVFFGYEAKGPGTPDIAYYEADPSGRIIHEAWLEAPYTSMVHDWAVTQNFV
IFPIIPLRSDIEWIKKGQSYFQWDPYEDVYLGVMPRKGTAKDLRWYRGSNRFASHIMGAYDDGRYVYIDTPVGQTNYFPWFPD
ITGAPYDPERSKGYLSRWTIDTQGESDGFTETRLSDLPGEFPRMDDRQETLPYHWGIMLLTDVPGRNAPGGGFRWLMGIDLKT
GRKQIYYPGDHCTLGEAIFVPAAPDAPPGVGYVLTVVARRKEMRSDLVLLDAQRLDGEPVCTLSLPMRIRMGVHGNWVSAAEL
AGRTD

13. ACO-08

MASWSHPQFEKGADDDDKVPDPMTTRFPQDPMFAGFSAPVRVEADIYDLEVAQGEVPGQLDGTYYRVVCDRQWPPMVAGDIPF
NADGMVMSFRFERGHVDFKSRYVRTPRFEAERKARRSLFGAYRNPFTDDPSVAGMNRTLANTNVFWHGGKLLASKEDSPPIQI
DPDTLDTVGVHTFDGALTSQTSTAHPKFDPRTGEMVFFGFAAKGECTPDIAFYEADRDGNLVHETWFQAPYSSMVHDFAVTQN
FVVFPIIPLVSDLGRIRAGLSHYAWDPGRDVHLGVLPRKGRVEDLRWYRGGTRFASHILGAYDDGRHIHIDTPVSESNYFPFF
PDLSGAPADPGKTKGYLSRWTIDTMGERGSFTQERLTTHAGEFPRMDDRFETLPYSWGVMGMYEVPGEPRPGRGFRWVGAVDL

EHRRTTTHYVGDCSSVGEPLFVPAHEKAGHAEGYVLAVVGRHDEMRSDLLILDAGRIDAPPVATVKLPIRVPYGLHGNWVTRG
ELQRG

14. ACO-09

MASWSHPQFEKGADDDDKVPDPMQQIRFPQNEFQSFEAPMRAEVDIRGLEVVQGEVPEHLDGGFYRLLGDRQWPSFIENDIFL
LNEDGMASCLRISNGRVDFRTRYVKTPRFVAEEQAGRALFGAYRNPLTDDPSVQGVRRGTANVTMLSHAGKFFALKEDSPPVE
MDPFTLETIGEYTFGGDFTSEAFTAHPKIDPRTGELVFYGYCAKGPATKDIAYYEADQTGKIIHETWFETPYTCMVHDIMVTE
NYVVFPITPLRHEYEWLEKREPAFKYDTSEQVHLGVLPRKGTADQIRWFVGPNQCHGHTVGAYDDGRYIYADNTLSVRSFFPF
FPNADGSPFDPEDTKPYMKRWVIDMGSEGDTFTERQLLQYPCELPRIDTRFETQPYSFCALNLLDVPGQDRVGGGWQWIATMD
VTGKKPTRIRYAGDNCSISEPQFVPAHDGAAEGEGYVLVVVGRHDEMRSEFLILDAQNIDGPPIATVALPMRVRSTGHGFWYG
KRQLMGETHLLGAN

15. ACO-10

MASWSHPQFEKGADDDDKVPDPMRTQAVATTELDRQVRTEGETRHDSLDGEHMTHQNNPEEDARAEELAWVRTQPKVPIVTPS
PYPMIRFAEPGHPFDRPVRVEADIEGLECVQGEIPAHLNGTYYKVVSDRQFPSFVENDLGYFNDDGMALSFRFHDGKVDYKSR
FIRTPRFEAEKAAGRSLFGKYRNQFDDDAMVEGMLRGNANTNLFFHGGKLYAAKEDSPPIILDPITLETLGVYDFEGSLTSKS
STAHPKIDPITGEMVFFGYAAKGDTTRDIAYYEADSTGQIIHETWFKAPYSAMVHDFAVTQNYVVFPIIPLRSEREWIEKGES
SFQWDASEDVYLGVMPRKGDGAKIRWFRGRSRFASHTLGAYDDGRYIYIDSPVSESKYFPWFPNRDGSPHDAVKGRTTLTRWT
IDLHDGSDGFTESSISDLGVEFPRMDDRFETLQHTWGAMAVSSVPGIEMQGHGFRWVSTVDYATGAQKLYYPGEGSTVGEPVF
VPGAPGAPEGHGYVLVLVSRRDAMHSDLVVLDAQNVDKEPVCTLRMPLRVPKGLHGNWVSDEELGQRTD

16. ACO-11

MASWSHPQFEKGADDDDKVPDPMTVRFPDEPGFQGRFAPVRLEGEIRGVEVTQGEIPASLSGTFYRGGADPAWPPFVERDFYF
NADGMVAMFRFANGYCDFRSRYVRTPRFVAERAARRSLFGAYRNPFTDDPSVAGLSRGLANTNVYWHGGRLLASKEDSAPVQI
DPDTLDTVGEFTWEGDLSSQTATAHPKIDPRDGSLVFFGYMAKGEATRDIAYYEADAAGRIVHEAWFEAPYSSMVHDWAVTEN
FVIFPIIPLTASLERLAAGGPLYVWDGSEDVYLGVVPRRGNSVRWYRGSNRFASHIMNAYDDGRHIHIDTPVGEKSAFPWFPD
IAGAPFDPEKASAQLTRWTIDTAAPEDLGDGATAFEQRRLTGCSGEFPRTDDRWATRGYRFGVINLTDVPGERPDDGLPGFRW
LGQIDPATGAMKTRFAGRNSTVQEAIFVPPGRDTANGEGYVMQLVDRHETGTTDLLILDAQRIDEPPVATLRIPIRMPGGLHG
NWVTDEQLAQRAG

17. ACO-12

MASWSHPQFEKGADDDDKVPDPMDRFPIPEDPFDAPMRLEADITDLECVQGEVPAHLDGTYYRVVVDRHYPSFVDNDLALFNS
DGMAMSFTFHKGRVDFKSRYVRTPRFVAEEKAGKSLFGAYHNALTDDPSVAGVRRGLANTNIFFHGGRLYASKEDSPPILLDP
VTLETLGEYDFQGALTSETSTAHPKIDPRTGEMVFFGYAAKGEATPDIAYYEADPNGQIIHETWIRAPYSCMIHDFAVTQNYV
VFPIVPLRSSIEWLEKGNPQFFWDPNEDVHLGVLPRKGTDKDLRWFRGSNRFASHIMGAYDDGRYLHLDSPVAETNYFPWFPD
LSGAPYNPDRSKTRLTRWTIDLSGESETFTERRLMDAAGEFPRMDDRQETLDYNWGVMAINDHPEQHKTGGGFRWVGAVDLSS
GRTKYYDPGENSTVGEPIFVPGNDTAGPGAGYVFVVVARRDHMHSELVILDAQHIDRPPVATLKLPMRLKSGLHGNWVGADEI
AGRTA

18. ACO-13

MASWSHPQFEKGADDDDKVPDPMVRFPPPKDPFDAPMRVEADIRGVQVTQGEVPEHLDGIYYRCVSDRQWPSYVENDLELFNS
DGMIISFRFHKGRVDFKSRYVRTPRFVAEEKAGRALFGAYRNPFDDDPSVAGISRGLANTNVFYHGGKLYASKEDSPPILLDP
ITLETIGEYDFEGALTSRTSTAHPKFDPITGEMVFFGYEAKGPGTPDIAYYEADASGRIIHEAWLEAPYTSMVHDWAVTQNFV
VFPIIPLRSDIEWIKQGRSYFQWDPYEDVYLGVMPRKGTAKDLRWYRGSNRFASHIMGAYDDGRYIYIDTPVGQTNYFPWFPD
ITGAPYDPERSKGYLSRWTIDTQGESDGFTETRLCDLPGEFPRMDDRQETLPYHWGIMLLTDGPGRVAPGGGFRWLMGIDLKT
GRKQIYYPGDNCTLGEAIFVPAAPDAPPGVGYVFTVVARRAEMRSDLVILDAQHIDGEPVCTLSLPMRIRMGVHGNWVSAEEL
ARRTA

19. ACO-14

MASWSHPQFEKGADDDDKVPDPMGIRFPNNDFKNFEAPMRAEIDLAGLEVVQGEVPKELSGGFFRLIGDRKWPAFVENDIFML
GEDGMASCFYFKDGRVDFKMRYVRTPRFVAEEKAGRALFGEYRNPFTDDPSVAGLSRGTANVTMLAHAGKLFAMKEDSPVIEM
DPFTLETIGEYDWGGAVTAKAITAHPKIDPRTGELIFFGYCAAGPATRDVAYYEADAEGRIIHEAWFKAPYTCMVHDIMVTEH
YVVFPITPLRHELEWIERREPAFKWDPNEEVYLGVLPRKGNGDQIRWFKGPNQCHGHTVGAFDDGRYIYADNTFSKRSFFHFF
PNVDGSAFSPADTMPYMKRIVIDMESEGDHFTEQKLFEYPCELPRIDTRFETMDYNFVTMNLLDVPGQERVGHGFQWVATMDV
NGNKPSKIYYAGDDCSIGEPQFVPKSNDAPEGEGYILTVVGKHQEMRSELLILDASDINAPPVATVALPFRLRSTGHGYWYST
SQLLGETNVLGAS

20. ACO-15

MASWSHPQFEKGADDDDKVPDPMNIRFSNNDFKSFEAPMRAEIELTGLEVVQGKVPKNLEGGFFRLIGDRKWPAFVDNDVFML
NEDGMASCFYFKDGKVDFRMRYVRTPRFVAEEKAGRALFGHYRNPFSDDPSVAGLSRGTANVTMLMHGGKLLALKEDSPAIEL
NPFTLETIGEYDWNGKVTAKAITAHPKIDAKTGELVFFGYCAAGPATRDIAYYEADSEGNVTHETWFHAPYTCMVHDIMVTQN
YVVFPITPLRHELEWIKRSEPAFKWDPHESVYLGVLPRKGRGDQIRWFKGPNQCHGHTVGAYDDGRYIYADNTFSKRSFFHFF
PNVDGSQFKPTDAMPYMKRVVIDMERSDNSFVEQPLFEYPCELPRIDTRFETLPYSFATMNLLDVPGQERVGRGFQWIATMDV
NGAKPSKILYAGDNCSIGEPQFVPAHDNAAEGDGYIMTVVGRHEQMRSELLILDASDINAPPVATVALPFRLRSTGHGYWYGG
RQLRGETNFPGAW

21. ACO-16

MASWSHPQFEKGADDDDKVPDPMAHIFDIAPAVKGYASGRLAKPGDATTFPDTDVFRGINKPSRFEGDIFDLEVTGTIPKDID
GTFYRVQPDHRFPPLFEDDIHFNGDGSVTAIRISNGHADFKQRYVQTDRYKAETAARRSLFGRYRNPWTDNESVKGVIRTASN
TNVTFWRGILLASKEDGPPFAMDPVTLETLGRYDFEGQIKAPTFTAHPKFDPDTGEMLCFAYEAGGDGNDGSCDVVVWTVDAD
GKKTEECWYKAPFAGMIHDMAVSKNFVVLPLTPLKVNMDRMKKGGEKFAWDPKEDQYYGLVPRRNAKPEDIRWFRADNGMAPF
LKTLSTHCADTPASSAFQGHIAGCYENEEGHVVVDLTVADGNVFFWWPPDDGTAIPPGKKPNQLSSTTTRWILDPKAETNTRI
TPAYVWPTNGEFSRIDDRFVTKKYKHFWQAKVDPTRPYDFAKCGPPAGGLFNCLGHYTWSPDNPLAKGEEDVFFFGPTATVQE
PSFIPKDEGGEGDGYLIALVNHLDVLRNDIAVFDAQHLAGGPLAVLHLPLKLKLGLHGNFVDHRDIEAWQRRRDAKGDVGPVK
VATEPLPWQKSFWDRRQDGQNGGLNGTH

22. ACO-17

MASWSHPQFEKGADDDDKVPDPMAHIFDLAPEVSLPDEPIYKDGKLHRPNHVQFPQTPVFASMNKPSRFEGTILSLEHTGIIP
PEINGTFFRVQPDHRFPPMFEDDIHFNGDGSVTAIRIFDGKVDFRQRYVHTERYKAETKARRSLFGRYRNPWTDNESVKGVIR
TASNTNVFFWRGMLLATKEDGPPYAMDPVTLETYGRYDFEGQILSPTFTAHPKFDPRTGEMVCFAYETGGDGADCSREVMVWT
LDKDGKKVSERWFEAPFAGMIHDCGLSENWLVLPLTPIKMDLERMKRGGNKFAWDPKEDQVYGLVPRRGDGEVKWFRGENAFH
GHVAGCYENAQGHVVIDLTVADGNVFFWFPPDGEEQGQFAKRNKLSSPTHRWILDPSLPNNARITPALVWPTNGEFSRIDDRW
TTRKYKHFWLAKVDPSRPYDFAKCGPPAGGLFNCLGHYTWDLDNELATGQEDVYFAGPTCTFQEPTFIPKGDKEGEGWLIALV
NHLDVLRNDVVILDAQNLAKGPVCTIHLPLKLKLGLHGNWVDWRDIEDWTKRRQEDGEVGPVQVATEMLPWQKAFWEKEKEKN
GNGVEGPNINGTNGANGTNGVNGSSH

23. ACO-18

MASWSHPQFEKGADDDDKVPDPMAHIFSLAPAVKGYKDGRLTRPGEATTFPPTPVFSGINKPSRFEGDVFDLEVTGTIPKEIN
ATFYRIQPDQRFPPLFEDDVHFNGDGSVTAIRISDGHADFKQRYVQTDRYKAETAARRSLFGRYRNPFTDNEAVKGVIRTASN
TNITFWRGMLLASKEDGPPFAMDPVTLETLGRYDFEGQITSPTFTAHPKFDPDTGEMICFAYEAGGNGNDGSLDIIMWTIDAD
GKKTEEAFYKAPFAGMIHDIGVSKNYVVLPLTPIKVNVDRMKRGGEKFAWDPYEDQWYGLVPRRNGKSEDIIWFRADNAFQGH
IAGCYENEDGHVVVDLTVADGNVFFWWPPDGSYDFPPFTTSELTPIQPDKITARDKLNSQTTRWILDPKAKTETRIKPAYVWD
INGEFSRIDDRWVTKKYKHFWQARIDPSKPYDFQKCGPPAGGLFNSLGHYTWNPDDALCHGEEDTYFFGPTSTVQEPSFIPRG
EDAAEGEGYLIALVNRLDELRNDVAVFDAQNVAQGPLAVIHLPLKLKLGLHGNFVDHRDIEAWQRRRDAGGDIGPVKIATEPL
PWQ

24. ACO-19

MASWSHPQFEKGADDDDKVPDPMAHIHDLAPQINQYYLGGRKQDNQLKFPDTDVFKGFNAPSRIEGEIINLEVDGTIPADIDG
TFYRIQPDHRYPPMFEDDIQFNGDGAVTAIRIQKGHADFKQRFVKTDRYEAETAERRALFGRYRNLYTDDELVKGVIRTASNT
NVVFWRGMLLAMKEDGPPFAMDPVTLETIGRYDFEGQVQSPTFTAHPKIDPVTGEMVCFGYEAGENGNDASNDIVVYTINADG
VKTDECWYKSPFCGMIHDCGISANYLVMPLTPLKCSLDRLKKGGNHWAWDPNEDQWYGVVPRRGGRPNDIKWFRSANAFHGHT
AGCYETEDGRIIYDLTVADGNVFFFFPPDDMPPGSVLKRNKLKSETCRWILDPKAPSGTYVTPAQSWNTSGEFSRIDDRLVTK
RYDHFWQCKIDPTREYDFASCGPPAGGLFNCLGHYTWSGQTEDLIALVNQLDVLRNDIMIFDALHLSAGPVATIRLPFKLKLG
LHGNFVDHADIAEWEALRRRAGGIGPVKPAERPLPWQLAMNGTASNGQSGRNCVNGLSH

25. ACO-20

MASWSHPQFEKGADDDDKVPDPMTHPFPQTPEFSGALYAPSRVEAEVFDLEVEGTLPDSIRGAFYQVAPDPQYPPMLGNDIFF
NGDGMVSAFNFADGKVSMRRRYVKTDRLMAQRREGRSLNGVYRNVFTNDPLAAKNNTTANTSVVAHNGVLLALKEDALPWAMD
LETLETLGEWSFDGQIESATFTAHPKLDPVTGNLLAFSYEAKGDGTPDLAYFELSPDGKLLHQIWFQAPYAAMVHDFAVTEHY
VVFPLIPLTVDVERMKNGGQHFQWQPDLPQLFAVVPRNGHAEDVRWFKGPKDGFQGHTLNAFDEDDKVYVDMPVTGGNIFYFF
PQADGYVPPPETLAASLMRWTFDLTSTQDDIQPQPLTDYPCEFPRCDDRYIGRKYEHGFVLAFDPERPYNPANGPIPFQFFNL
LARVNLKTGATDAWFPGDSGCFQEPIFIPRAPDAQEADGYVVALLNLIAEGRSELVVLDSRDMASGPIARIKVPFRLRMSLHG
CWVPSKK

26. ACO-21

MASWSHPQFEKGADDDDKVPDPMTIPFPQTPEFSGALYTPSRVEAEVFDLEIEGALPASIRGTFYQVAPDPQYPPMLGTDIFF
NGDGMVSGFYFANGKVSLRRRYVKTDRLLAQRREGRSLNGIYRNVYTNDPLAAKNNTTANTTVVPHNGVLLALKEDAMPWAMD
LETLETLGEWNFDGQIKSATFTAHPKLDPATGNLLAFSYEAKGEGTPDLAYFELSPDGKLLHEIWFQAPYAAMVHDFAVTEHY
VVFPLIPLTVDIERMKNGGPHFQWQPDLPQLFAVVPRNGCAQDVRWFKGPKEGFQGHTLNAFDEDGKVYVDMPVTGGNIFYFF
PQADGYVPPPETLAASLMRWTFDLNGSQDEIQPQPLTDYPCEFPRCDDRYIGRKYEHGFLLAFDPQRPYNPENGPIPFQFFNL
LAHVNLKTGTTDAWFPGDSGCFQEPIFIPRSPNAEEADGYVVSLLNLIAEGRSELVVLDSRDMESGPIARIKVPFRMRMSLHG
CWAPS

27. ACO-22

MASWSHPQFEKGADDDDKVPDPMTIPFPQTSEFSGALYTPSRVEAELFDLEVEGTLPAEIRGAFYQVAPDPQYPPMLGTDIFF
NGDGMVTGFYFANGKVSMRRRYVKTDRLMAQRREGRSLNGVYRNLYTNDPLAAKNNTTANTTVIPHNGVLLALKEDALPWAMD
LETLETLGEWTFDGQIKAATFTAHPKRDPATGNLLAFSYEAKGDGTPDIAYFEISPDGKLLHEIWFQAPYAAMVHDFAVTERY
VVFPLIPLTVDVERMKNGGPHFQWQPDLPQLFAVVPRNGNAQDVRWFKGPKDSFQGHTLNAFDEDGKVYVDMPVTGGNVFYFF
PQADGYVPPPETLAASLMRWTFDLSSPQDEVEPQPLTEYPCEFPRCDDRYIGRKYQHGFLLAFDPERPYNPANGPIPFQFFNL
LVHLNLKTGSTDAWFPGDSGCFQEPIFIPRSADAEEADGYVVALLNLIAEGRSELVVLDSRDMAGGPIARIRIPFRMRMSLHG
CWAPSD

28. ACO-23

MASWSHPQFEKGADDDDKVPDPMTIPFPQTPEFSGALYTPSRVEAEVFDLEIEGVLPASIGGTFYQVAPDPQYPPMLGNDIFF
NGDGVVSRFNFANGKVSMRRRYVKTDRLLAQRREGRSLNGVYRNVYTNDRLAAKNNTTANTTVIPHNGVLLALKEDALPWAMD
LDTLETLGEWTFDGQIKSATFTAHPKLDPVTGNLLACSYEAKGDGTPDLAYFEISPDGKLLHEIWFQAPYAAMVHDFAVTERY
VVFPLIPLTVDVERMKNGGPHFQWQPDLPQLFAVVPRNGNAQDVRWFKGPKDGFQGHTLNAFDEDGKVYVDMPVTGGNIFYFF
PQADGYVPPPETLAASLMRWTFDLNSALDEVEPQPLTDYPCEFPRCDDRYIGRKYQHGFLLAFDPERPYNPANGPIPFQFFNL
LVHLNLKTGITDAWFPGDSGCFQEPIFIPRSADAEEADGYVVALLNLIAEGRSELVVLDSRDMASGPIARIRIPFRMRMSLHG
CWTADPN

29. ACO-24

MASWSHPQFEKGADDDDKVPDPMSIPFPQTPEFSGVLYTPSRVEAEVFDLEIEGILPASICGTFYQVAPDPQYPPMLGNDIFF
NGDGVVSRFNFANGKVSMRRRYVKTDRLLAQRREGRSLNGVYRNVYTNDPLAAKNNTTANTTVIPHNGVLLALKEDALPWALD
LETLETLGEWTFDGQIKAATFTAHPKLDPVTGNLLACSYEAKGDGTPDLAYFEISPDGKLLHEIWFQAPYAAMVHDFAVTERY
VVFPLIPLTVDVERMKNGGPHFQWQPDLPQLFAIVPRNGCGQDVRWFKGPKDGFQGHTLNAFDEDGKVYVDMPVTGGNIFYFF
PQADGYVPPPETLAASLMRWTFDLNGAQEDVQPQPLTEYPCEFPRCDDRYIGRKYQHGFLLAFDPERPYNPANGPIPFQFFNL
LAHLDLQTGRTDAWFPGDSGCFQEPIFIPRSADAEEADGYVVALLNLIAEGRSELVVLDTRDMASGPIARIRIPFRMRMSLHG
CWAPND

LIST OF DNA SEQUENCES

Restriction sited are underlined, start and stop codons are marked in red and tags are marked in blue.

1. Eugenol oxidase from *Rhodococcus Jostii*

CCATGACCCGTACCCTGCCGCCGGGCGTGAGTGATGAACGCTTTGATGCCGCCCTGCAGCGTTTTCGTGATGTTGTGGGTGAC
AAATGGGTGCTGAGTACCGCCGATGAACTGGAAGCATTTCGTGATCCGTATCCGGTGGGCGCAGCAGAAGCAAATCTGCCGAG
TGCAGTTGTTAGTCCGGAAAGTACCGAACAGGTTCAGGATATTGTTCGTATTGCCAATGAATATGGTATTCCGCTGAGCCCGG
TTAGTACCGGTAAAAATAATGGCTATGGCGGTGCAGCACCGCGCCTGAGTGGTAGTGTTATTGTTAAAACCGGCGAACGTATG
AATCGTATTCTGGAAGTTAATGAAAAGTACGGTTATGCCCTGCTGGAACCGGGTGTTACCTATTTTGATCTGTATGAATATCT
GCAGAGTCATGATAGTGGTCTGATGCTGGATTGTCCGGATCTGGGCTGGGGTAGCGTTGTGGGTAATACCCTGGATCGCGGTG
TGGGCTATACCCCGTATGGCGATCATTTTATGTGGCAGACCGGTCTGGAAGTGGTTCTGCCGCAGGGTGAAGTGATGCGTACC
GGCATGGGCGCACTGCCGGGCAGTGATGCATGGCAGCTGTTTCCGTATGGTTTTGGTCCGTTTCCGGATGGCATGTTTACCCA
GAGTAATCTGGGTATTGTTACCAAAATGGGTATTGCACTGATGCAGCGCCCGCCGGCCAGTCAGAGCTTTCTGATTACCTTTG
ATAAAGAAGAAGACCTGGAACAGATTGTTGATATTATGCTGCCGCTGCGCATTAATATGGCACCGCTGCAGAATGTTCCGGTG
CTGCGTAATATTTTTATGGATGCAGCAGCCGTTAGCAAACGTACCGAATGGTTTGATGGTGACGGCCCGATGCCGGCAGAAGC
CATTGAACGCATGAAAAAAGATCTGGATCTGGGTTTTTGGAATTTTTATGGCACCCTGTATGGCCCGCCGCCGCTGATTGAAA
TGTATTATGGCATGATTAAGGAGGCCTTTGGTAAAATTCCGGGTGCACGTTTCTTTACCCATGAAGAACGTGATGATCGCGGT
GGTCATGTTCTGCAGGATCGCCATAAAATTAATAATGGTATTCCGAGTCTGGATGAACTGCAGCTGCTGGATTGGGTTCCGAA
TGGTGGCCATATTGGTTTTAGTCCGGTGAGTGCCCCGGATGGTCGCGAAGCCATGAAACAGTTTGAAATGGTTCGCAATCGTG
CAAATGAATATAATAAGGATTACGCAGCCCAGTTTATTATTGGTCTGCGTGAAATGCATCATGTTTGTCTGTTTATCTATGAT
ACCGCCATTCCGGAAGCACGCGAAGAAATTCTGCAGATGACCAAAGTTCTGGTGCGCGAAGCCGCAGAAGCCGGTTATGGTGA
ATATCGCACCCATAATGCCCTGATGGATGATGTGATGGCAACCTTTAATTGGGGCGATGGTGCACTGCTGAAATTTCATGAAA
AAATTAAGGACGCCCTGGATCCGAATGGTATTATTGCCCCGGGCAAAAGCGGCATTTGGAGTCAGCGTTTTCGCGGCCAGAAT
CTGCATCATCATCATCACCATTAATAAAAGCTT

2. Isoeugenol cleavage oxygenase from *Pseudomonas sp.*

GGATCCCATGTTCCCGACCCGCATTGAAGCAAATGTGTTTGATCTGGAAATTGAAGGTGAAATTCCGCGTGCCATTAATGGCA
GTTTTTATCGTAATACCCCGGAACCGCAGGTGACCACCCAGCCGTTTCATACCTTTATTGATGGCGATGGTATGGCCAGTGCC
TTTCATTTTGAAGATGGCCATGTTGATTTTATTAGCCGTTGGGTGTGTACCCCGCGCCTGGAAGCAGATCGCAGCGCCCGTAA
AAGCCTGTTTGGCATGTATCGCAATCCGTTTACCGATGATCCGAGTGTTGAAGGTATTGATCGCACCGTGGCCAATACCAGTA
TTATTACCCATCATGGTAAAGTTCTGGCAGCAAAAGAAGATGGTCTGCCGTATGAACTGGACCCTCAGACCCTGGAAACCCGT
GGTCGCTATGATTATAAAGGTCAGGTGACCAGCCATACCCATACCGCCCATCCGAAATTTGATCCGCAGACCGGTGAAATGCT
GCTGTTTGGCAGTGCAGCAAAAGGTGAAGCAACCCTGGATATGGCCTATTATATTGTTGATCATTACGGTAAAGTGACCCATG
AAACCTGGTTTAAACAGCCGTATGGTGCCTTTATGCATGATTTTGCAGTTACCCGTAATTGGAGTATTTTTCCGATTATGCCG
GCAACCAATAGCCTGGAACGTCTGAAAGCACGTCAGCCGATCTATATGTGGGAACCGGAACTGGGTAGCTATATTGGCGTTCT
GCCGCGCCGTGGCCAGGGCAAAGATATTCGCTGGTTTCGCGCCCCGGCACTGTGGGTGTTTCATGTGGTTAATGCCTGGGAAG
AAGGCAATCGTATTCTGATTGATCTGATGGAAAGCGAAATTCTGCCGTTTCCGTTTCCGAATAGTCAGAATCTGCCGTTTGAT
CCGAGCAAAGCAGTGCCGCGTCTGACCCGCTGGGAAATTGATCTGAATAGTGGTAATGATGAAATGAAACGCACCCAGCTGCA
TGAATATTTTGCCGAAATGCCGATTATGGATTTTCGTTTTGCACTGCAGGATCATCGTTATGCATATATGGGCGTGGAAGATC
CGCGTCGCCCGCTGGCACATCAGCAGGCTGAAAAAATTTTTGCATATAATAGCCTGGGTGTTTGGGAAAATCATCGTAAAGAA
TATGATCTGTGGTTTACCGGTAATATGAGCGCCGCACAGGAACCGGCATTTGTGCCGCGCAGCCCGGATGCACCGGAAGGTGA
CGGTTATCTGCTGAGCGTTGTTGGTCGCCTGGATGAAGATCGCAGTGATCTGGTTATTCTGGATACCCAGTGCCTGGCCGCAG
GTCCGGTGGCAACCGTTAAACTGCCGTTTCGTCTGCGTGCCGCCCTGCATGGTTGTTGGCAGAGTAAAAATTAATAAAAGCTT

30. Isoeugenol cleavage oxygenase from *Pseudomonas putida*

GGATCCCATGGCAACCTTTGATCGCAATGATCCGCAGCTGGCCGGCACCATGTTTCCGACCCGTATTGAAGCAAATGTTTTTG
ATCTGGAAATCGAAGGCGAAATTCCGCGTGCCATTAATGGCAGCTTTTTCCGTAATACCCCGGAACCGCAGGTTACCACCCAG
CCGTTTCATACCTTTATTGATGGTGACGGTCTGGCCAGCGCCTTTCATTTTGAAGATGGTCAGGTTGATTTTGTTAGCCGCTG
GGTGTGCACCCCGCGCTTTGAAGCAGAACGCAGCGCACGCAAAAGCCTGTTTGGCATGTATCGTAATCCGTTTACCGATGATC
CGAGCGTGGAAGGTATTGATCGCACCGTTGCCAATACCAGCATTATTACCCATCATGGCAAAGTGCTGGCAGCAAAAGAAGAT
GGTCTGCCGTATGAACTGGACCCTCAGACCCTGGAAACCCGCGGCCGTTATGATTATAAAGGCCAGGTGACCAGCCATACCCA
TACCGCACATCCGAAATTTGATCCGCAGACCGGTGAAATGCTGCTGTTTGGTAGTGCCGCCAAAGGTGAACGCACCCTGGATA
TGGCATATTATATTGTGGATCGTTATGGTAAAGTGACCCATGAAACCTGGTTTAAACAGCCGTATGGTGCATTCATGCATGAT
TTTGCAGTGACCCGCAATTGGAGCATTTTTCCGATTATGCCGGCCACCAATAGTCTGGAACGCCTGAAAGCAAAACAGCCGAT
CTATATGTGGGAACCGGAACGTGGCAGTTATATTGGTGTGCTGCCGCGTCGTGGTCAGGGTAAAGATATTCGTTGGTTTCGCG
CACCGGCACTGTGGGTGTTTCATGTTGTGAATGCCTGGGAAGAAGGTAATCGCATTCTGATTGATCTGATGGAAAGTGAAATT
CTGCCGTTTCCGTTTCCGAATAGCCAGAATCTGCCGTTTGATCCGAGTAAAGCAGTGCCGCGCCTGACCCGCTGGGAAATTGA
TCTGAATAGCGGCAATGATGAAATGAAACGCACCCAGCTGCATGAATATTTTGCAGAAATGCCGATTATGGATTTTCGCTTTG

CCCTGCAGGATCATCGCTATGCCTATATGGGTGTGGATGATCCGCGTCGTCCGCTGGCACATCAGCAGGCAGAAAAAATTTTT
GCATATAATAGCCTGGGCGTGTGGGATAATCATCGTAAAGATTATGAACTGTGGTTTACCGGCAAAATGAGCGCAGCCCAGGA
ACCGGCATTTGTTCCGCGTAGCCCGGATGCCCCGGAAGGCGATGGCTATCTGCTGAGCGTTGTGGGCCGTCTGGATGAAGATC
GTAGCGATCTGGTTATTCTGGATACCCAGTGTCTGGCCGCAGGTCCGGTGGCAACCGTTAAACTGCCGTTTCGCCTGCGCGCC
GCACTGCATGGTTGTTGGCAGAGTAAAAATTAATAAAAGCTT

3. Isoeugenol cleavage oxygenase from *Pseudomonas nitroreducens*

GGATCCCATGGCACGCCTGAATCGCAATGATCCGCAGCTGGTTGGTACCCTGCTGCCGACCCGTATTGAAGCAGATCTGTTTG
ATCTGGAAGTGGATGGTGAAATTCCGAAAAGCATTAATGGTACCTTTTATCGTAACACCCCGGAACCGCAGGTGACCCCGCAG
AAATTTCATACCTTTATTGATGGTGACGGTATGGCCAGTGCATTTCATTTTGAAGATGGCCATGTTGATTTTATTAGTCGTTG
GGTTAAAACCGCCCGCTTTACCGCCGAACGCCTGGCCCGTAAAAGTCTGTTTGGCATGTATCGTAATCCGTATACCGATGATA
CCAGCGTTAAAGGTCTGGATCGTACCGTGGCAAATACCAGCATTATTAGCCATCATGGCAAAGTGCTGGCAGTGAAAGAAGAT
GGCCTGCCGTATGAACTGGACCCTCGTACCCTGGAAACCCGCGGTCGCTTTGATTATGATGGTCAGGTGACCAGTCAGACCCA
TACCGCCCATCCGAAATATGATCCGGAAACCGGCGATCTGCTGTTTTTCGGCAGCGCCGCCAAAGGTGAAGCAACCCCGGATA
TGGCCTATTATATTGTGGATAAACATGGTAAAGTGACCCATGAAACCTGGTTTGAACAGCCGTATGGTGCATTCATGCATGAT
TTTGCAATTACCCGCAATTGGAGTATTTTTCCGATTATGCCGGCAACCAATAGCCTGAGTCGTCTGAAAGCCAAACAGCCGAT
CTATATGTGGGAACCGGAACTGGGTAGCTATATTGGCGTTCTGCCGCGTCGCGGCCAGGGCAGCCAAATTCGCTGGCTGAAAG
CACCGGCACTGTGGGTGTTTCATGTGGTGAATGCATGGGAAGTTGGCACCAAAATCTATATTGATCTGATGGAAAGCGAAATT
CTGCCGTTTCCGTTTCCGAATAGTCAGAATCAGCCGTTTGCACCGGAAAAAGCCGTTCCGCGCCTGACCCGTTGGGAAATTGA
TCTGGATAGCAGCAGCGATGAAATTAAGCGCACCCGCCTGCATGATTTCTTTGCAGAAATGCCGATTATGGATTTTCGTTTTG
CCCTGCAGTGCAATCGCTATGGCTTTATGGGTGTTGATGATCCGCGTAAACCGCTGGCCCATCAGCAGGCCGAAAAAATTTTT
GCATATAATAGTCTGGGCATCTGGGATAATCATCGTGGCGATTATGATCTGTGGTATAGCGGCGAAGCCAGTGCAGCCCAGGA
ACCGGCCTTTGTGCCGCGTAGCCCGACCGCTGCAGAAGGCGATGGTTATCTGCTGACCGTTGTGGGTCGCCTGGATGAAAATC
GCAGCGATCTGGTTATTCTGGATACCCAGGATATTCAGAGCGGCCCGGTGGCAACCATTAAGCTGCCGTTTCGCCTGCGCGCC
GCACTGCATGGTTGTTGGGTGCCGCGTCCGTAATAAAAGCTT

4. Lignostillbene dioxygenase from *Pseudomonas brassicacerarum*

GGATCCCATGAGCATTCCGTTTCCGCAGACCCCGGAATTTTCTGGCGCACTGTATAAACCGAGCCGTATTGAAGCAGAAGTTT
TTGATCTGGAAATTGAAGGCGTGCTGCCGGCCAGCATTCATGGTACCTTTTATCAGGTGGCACCGGACCCTCAGTATCCGCCG
ATGCTGGGTACCGATATTTTCTTTAATGGCGATGGCATGGTTAGTGGCTTTCATTTTGCAAATGGTAAAGTTAGTCTGCGTCG
TCGTTATGTTCAGACCGATCGTCTGCTGGCCCAGCGCCGCGAAGGCAGAAGTCTGAATGGTGTTTATCGTAATGCATTCACTA
ATGATAGTCTGGCAGCAAAAAATAATACCACCGCAAATACCAGTGTTATTCCGCATAATGGCGTTCTGCTGGCCCTGAAAGAA
GATGCACTGCCGTGGGCAATGGATCTGGAAACCCTGGAAACCTTAGGCGAATGGACCTTTGATGGCCAGATTAAGAGTGCAAC
CTTTACCGCACATCCGAAACTGGACCCTGCAACCGGCAATCTGCTGGCCTTTAGTTATGAAGCAAAAGGCGATGGTACCCCGG
ATCTGGTGTATTTTGAACTGAGCCCGGATGGTAAACTGCTGCATGAAATTTGGTTTCAGGCACCGTATGCAGCAATGGTTCAT
GATTTTGCCGCCACCGAACGTTATGTGGTTTTTCCGCTGATTCCGCTGACCGTGGATGTGGAACGTATGAAAAATGGTGGCCC
GCATTTTCAGTGGCAGCCGGATCTGCCGCAGCTGTTTGCAGTGGTGCCGCGCAATGGCCGCGCACAGGATGTGCGCTGGTTTA
AAGGTCCGATGGATGGCTTTCAGGGTCATACCCTGAATGCATTTGATGAAGATGGTAAAGTTTACGTTGATATGCCGGTGACC
GGCGGCAATATTTTCTATTTCTTTCCGCAGGCAGATGGCCATGTGCCGCCGCCTGAAACCCTGGCAGCATGCCTGATGCGCTG
GACCTTTGACCTGAATAGTGGCCGTGATGAAGTGGAACCGCAGCCGCTGACCGATTATCCGTGTGAATTTCCGCGTTGTGATG
ATCGTTATATTGGTCGCCAGTATGCACATGGCTTTCTGCTGGCCTTCGATCCGGAACGTCCGTATAATCCGGCCAATGGCCCG
ATTCCGTTTCAGTTTTTCAATCTGCTGGTGCATCTGAATCTGAAAACCGGCCTGAGTGATGCATGGTTTCCGGGTGACAGTGG
TTGCTTTCAGGAACCGATTTTTATTCCGCGTAGTGCCGATGCCGAAGAAGCAGATGGCTATGTTGTGGCCCTGCTGAATCTGA
TTGCAGAAGAACGCAGTGAACTGGTGGTTCTGGATAGCCGTGATATGGCCAGTGGTCCGATTGCCCGTATTCGCATTCCGTTT
CGTATGCGTATGAGTCTGCATGGCTGCTGGGCCCCGGGCTAATAAAAGCTT

5. Aromatic dioxygenase from *Thermothelomyces thermophilus* - ACO-01

GGATCCCATGGCACATATTCATGATCTGGCACCGGAAGTGAGCAATTATAGCAGTGGTCGTCTGACCCCGCCGACCCCGGTTC
GCTTCCCTCGTACCCCGGTGTTCGCAAGCATGAATAAACCGTGCCGCTTCGAAGGCGATGTGTTCGATCTGGAAGTTAGCGGT
GCCATTCCGCCGGATATTGATGGCACCTTCTTCCGCGTGCAGCCGGATCATCGCTTCCCGCCGCTGTTCGAAGATGATATTCA
CTTCAATGGTGATGGTAGTGTTACCGCAATTCGTATTAGCGGCGGTCATGCAGATCTGCGCCAGCGCTATGTGCGCACCGAAC
GCTATCTGCTGGAAACCAGAGCCCGTCGCAGTCTGTTCGGCCGCTATCGTAATCCGTGGACCGATAATGAAAGCGTTCGCGGT
GTGATTCGTACCGCAAGTAATACCAATGTTGTGTTCTGGCGCGGTGCACTGCTGGCCATGAAAGAAGATGGCCCGCCGTTCGC
CATGGACCCTGTGACCCTGGAAACCTTAGGTCGTTATGACTTCGAAGGTCAGATTCTGAGTCCGACCTTCACCGCCCATCCGA
AAATTGATCCGGATACCGGCGAAATGGTGTGCTTCGCATACGAAGCCGGTGGTGATGGCAGTGATTGTAGCGTTGATGTGGCA
GTGTGGACCGTTGATGCAGATGGTAAAAAAGTGGAAGAATGCTGGTATAAAGCCCCGTTCGCAGGCATGATTCATGATTGTGG
CATTACCAAAAATTGGGTGGTTCTGCCGCTGACCCCGATTAAAATGGATCTGGAACGTATGAAACGTGGTGGCAATAAATTCG
CATGGGACCCTAGTGAAGATCAGTGGTATGGTGTGGTTCCGCGTCGCGGTGCCAAAAGTGATGATATTATCTGGTTCCGCGCC
GATAATGGCTTCCATGGCCATGTTGCAGGCTGCTATGAACTGCCGAGTGGCGAAATTGTGTTCGATCTTACCGTGGCCGATGG
TAATGTGTTCTTCTTCTTCCCGCCGGATGATAATATTACCCCGCCGGCCGATGGTGTGGCAAAACGTAATCGCCTGAGTAGCC
CGACCGTTCGTTGGATCTTCGATCCGAAAGCAAAAAAAAGCGCAATTCGCACCGAAGCAGCCGGCGATGCCGATATCTGGGTT

GCCGATGAACGCGTGAAACCGGCACTGACCTGGCCGACCAATGGCGAATTCAGCCGTATTGATGATCGCTATGTGACCAAACC
GTATCGCCACTTCTGGCAGGCAGTGGTGGACCCTACCCGCCCGTATGACTTCGAGAAATGTGGTCCGCCGGCAGGCGGTCTGT
TCAATTGCCTGGGCCATTATACCTGGAGTGATCAGAATTATCATCATGGTCATAATACCGGTGATCCGAGCGGTGATGGTCGC
AGCAATGGCAGTGCAGAAGAAGCAACCGCAGGTAAATTCGGTCTGCAGGATGTGTACTTCGCAGGTCCGACCATGACCTTCCA
GGAACCGACCTTCATTCCGCGCCAGGGTGCAGCCGAAGGTGAAGGTTATCTGATTGCACTGCTGAATCATCTGGATGAACTGC
GTAATGATGTGGTGATCTTCGAAGCACGTAATCTGGGTAAAGGTCCGCTGGCAGTTATTCATCTGCCGCTGAAACTGAAACTG
GGCCTGCATGGCAATTGGGTGGATAGTCGTGAAATTGAAGCATGGCGTCGTCGCCGTGCCGAAAATGGCGATGTTGGTCCGCT
GCGTGTGGCCAAAGAACCGCTGCCGTGGCAGAAAAAATTCGCAGCAGCAGCCCAGAATGGTAGTAATGGTGTTTAATAAAAGC
TT

6. ACO-02

GGATCCCATGACCCGCTTCCCGCAGCGTGAAGGCGCAAGCGCATTCGAAGCACCGATGCGCGTGGAAGCCGATATTGCAGGTC
TGGAAGTTGTGCAGGGCGAAGTTCCGGAACATCTGAGTGGCACCTATTATCGTGTTGTGACCGATCGTCAGTGGCCGCCGGCA
GTGCTGCCGGATGTGCCTACCTTCAATGATGATGGTATGGCAATGTACTTCCGCTTCCATGGTGGCCGCGTTGACTTCCGTAG
TCGCTATGTGCGTACCCCGCGTTATGAAGCAGAAGCCAAAGCAGGCCGCGCACTGTTCGGCGCATATCGTAATCCGCTGACCG
ATGATCCGAGCGTGGCAGGTATTAGCCGCGGTCTGGGTAATACCAATGTGTTCTATCATGGCGGCAAACTGTATGCAAGTAAA
GAAGATAGCGCCCCGATTCTGCTGGACCCTATGAGTCTGGAAACCATTGGTGAATATACCTTCGAAGGCACCCTGACCAGTCG
TACCTGTACCGCCCATCCGAAAGTGGACCCTCGCACCGGCGAAATGGTGTTCTTCGGTTATGCCGCCAAAGGTGAAACCACCC
CGGATATTGCCTATTATGAAGCCGATCAGGATGGCCGTATTATTCATGAAACCTGGATTCGTGCCCCGTATAGTAGTATGGTT
CATGATTGGGCCGTTACCCAGAACTTCGTTGTGTTCCCGATTATTCCGATGCGTAGCGAATATGCCTGGCTGGAAGCCCGCCA
GCCGCACTTCCAGTGGGACCCTGATCGTGATGTGTATCTGGGTGTGCTGCCGCGCAAAGGTAGTGCCGATCAGGTTCGTTGGC
TGCGTGGTAGTAATCGCTTCGCCAGCCATATTCTGGGTGCCTGGGATGATGGTCGTCATATTCATATTGAAACACCTGTTGGT
CGCAGCAACTTCTTCCCGTTCTTCCCGGATGTTACCGGTGCACCGTATGATCCGGTGCGCGCACGTGGTTATCTGAGCCGCTG
GACCATTGATATGGGTAGTGATGGCGGCACCTTCACCGAACAGCGTCTGACCGATGTGGCAGGTGAATTCCCGCGTATGGATG
ATCGCTTCGAAACCTTACCGTATCGTCGCGGCTTCCTGGCACTGAATCATGTTCCGGGCGAACAGCGCCCGGGCGCTGGCTTC
AGATGGATTGGCGCATTCGATACCACCGGTGCAGCACCGCCGCAGATCTATTATCCGGGTGATGATTGCAGCGTTGCCGAACC
GCTGTTCGTGCCGGGTGGTGATGATGCACCGGAAGGTCATGGCTATGTGTTCGTTGTTGTGGGTCGCCATCAGGAAATGCGCA
GCGATCTGGTTATTCTGGATGCACAGCGTCTGGATGCACCGCCGGTTGCAACCGTTGCACTGCCGATTCGCATTCGTCGCGGT
CTGCATGGCAATTGGGTGAGCAGCACCGAACTGGCAGCCCGTACCGCCTAATAAAAGCTT

7. ACO-03

GGATCCCATGAGCCTGTTCAATCGCAATCATCCGGTGTTCAGCGGTGGCAATGCACCGGTTCGCATTGAAACCGATGCAATCT
GTCTGGAAACCGATGGCGCACTGCCGGATGAACTGAAAGGCAGCTACTTCCGTCTGACCGCAGATCCGCAGTTCCCGCCGCGC
CTGCGTCCTGTTCTGGTGGAAGCAGATGGTCATGTTGGTGCATTCCACTTCCGTGATAATGGTCAGGTGGATTATGTTGGCAA
ATGGGTGCGTACCGAACGCTTCCTGCGCGAACGTGCCGCCCGTCGTGCCTTATTCGGTACCTATCGTAATCGCTTCACCGATG
AAGAAAGCGTGCTGGATAGCGATCGTACCACCGCAAATACCGCCTTCATGTTCCATCATCATAAATTCTTCGCCCTGAAAGAA
GATGGTCTGCCGTATCAGCTGGACCCTGAAACCTTAGATACCATTGGCCGCTATGACTTCGGTGGCACCGTTAGTGCAAAAAG
CCTGAGTGCACATCCGAAAGTGGATAGCCGTACCGGTGAACTGATTACCCATAGTGGTCAGGCCAAAGGCGAAGGTACCCCGG
ATATTGCCTATTATGTGTTCGATAAAAACGGCACCAAAACCGTTGAACGTTGGTTCGAAGCCCCGTATAGCAGTATTGTGCAT
GATATTGCCATTACCCAGGATTGGGTGGTGCTGCCGATTATGCCGGCCGTTGTGGAAGAAGAACGCCTGCGCGCAGGTGGCGC
AACCTATTGGTGGAAACCGGAACTGGGCAATCATATTGCAGTGTTCCGCCCGTGATGGTACCGGTGATGTTCGCTGGTTCCGTA
CCAAAGCCACCTATGCCTTCCATGTTGTTAATAGCTATCAGGATGGCGATCGTCTGATTATTGATGTTATGGATGCAGAAGAA
TTCCCGATGTGGTGGCCGCGCCCGGAACAGGTTGCCGCACTGCGTAGTGGCGAAATTAAACGTGATAAATTCGTGGCACAGCT
GACCCGCTGGACCATTGATCTGGCCGGTGATAGTGATGATATTGAACGCGAACTGCTGCATCCGTGGGAAGCAGAAATGCCGC
GCATTGATGATCGCTTCGCAGGTCAGCCGTATCGTTATGCCGTGTATGGCGTTGATGATCCGAGCTTCCCGCTGGCCCATGGC
CTGGCTGAACTGGGCGTGAATCATAATAGTGTTGGCTGGTGGGATCATCAGACCCGTACCCTGACCAGCTGGTATACCGGCCC
GAATAGTAGTGTGGGTGAACCGGTGTTCACCCCGCGTAGTCCGGATGCACCGGAAGGCGATGGCTTCATTCTGGCCGTGGTGC
AGCGCCTGGCCGAACAGCGTAGCGAACTGGTTGTTATTGATACCCGTGATGTTGCCGCCGGCCCGATTGCCAGCGTTCATGCC
CCGCATCGTCTGAAAAATGCCATTCATAATCTGTGGATTGATCAGGAACAGCTGAATGAAGGCGGTGAAAAAGCATAATAAAA
GCTT

8. ACO-04

GGATCCCATGATCCATGTTGCCAGTCCGGATGATCGCGGTAGCACCGTGCATCCGAAAGAAACACATGTGATTCGCTTCCCGG
TGACCGATGATCCGTTCGATGCCCCGATGCGTGTGGAAGCCGATATTACCGATCTGGAATGCACCCAGGGTGCAGTTCCGGAA
CATCTGGATGGTACCTATTATCGTGTGGTTGTTGATCGTCATTATCCGAGTATGGTTGATAATGATCTGGCACTGTTCAATAG
CGATGGCATGGCCATGAGCTTCACCTTCAGCAAAGGTCGTGTTGACTTCAAAACCAGATATGTTCGTACCCCGCGCTTCGTGG
CCGAAGAAAAAGCAGGCAAAAGTCTGTTCGGCGCATATCGCAATGCCAGTACCGATGATCCTAGTGTGGCAGGTATTCGTCGT
GGTCTGGCAAATACCAATGTGTTCTTCCATGGCGGCCGTCTGTATGCAAGCAAAGAAGATAGTCCGCCGATTCTGCTGGACCC
GTTCAGTCTGGAAACCTTAGGTGAATATGACTTCCAGGGTGCCCTGACCAGCGAAACCAGCACCGCCCATCCGAAAGTGGACC
CTCGTACCGGCGAAATGGTGTTCTTCGGTTATGGTGCAAAAGGCGAAGCCACCCCGGATATTGCCTATTATGAAGCCGATGCC
AGCGGTCAGATTATTCATGAAGCCTGGATTACCGCACCGTATAGTTGCATGATTCATGACTTCGCCGTTACCCAGAATTATGT

TATCTTCCCGATTGTGCCGCTGCGTAGCAGTACCGAATGGATTGCCCAGGGTAATAATCAGTACTTCTGGGACCCTGATGAAG
ATGTGTATCTGGGTGTGCTGCCGCGTAAAGGCAGTGGCAAAGATCTGCGCTGGTTCCGCGGTAGCAATCGCTTCGCAAGCCAT
ATTATGGGCGCCTTCGATGATGGTCGCCATATCTATATTGATACCCCGGTGGCAGAAACCAGTTACTTCCCGTGGTTCCCGGA
TATTAGCGGCGCAAGTTATGATCCGGATCGCAGTAAAACACATCTGAGTCGCTGGACCATTGATCTGGCCGATAGTACCGGTG
GCTTCACCGAACGCCGTCTGATGACCGCCGCCGGCGAATTCCCGCGTATGGATGATCGCCAGGAAACCTTAGCCTATGATTGG
GGCGTGCTGGCCATTAATGATCTGCCGGGCAGCCCGAAACCGGGCCCTGGCTTCCGTTGGATTGGCGCAGTTGATCTGCCGAG
TGGTAGCACCAAATTCTATGATCCGGGCCCGAATGCCACCGTGGGCGAACCTATCTTCGTTCCGGGTCAGGCAGATGCCAAAC
CGGGTCATGGTTATGTGTTCGTGGTTGTGGCACGCCGTGATCTGATGCGCAGCGATCTGGTGATTCTGGATGCACAGAATCTG
GATAAACCGCCGGTTGCCACCCTGAGCCTGCCGATTCGTATTAAAAGCGGTCTGCATGGTAATTGGGTGGGTGCCGATGAACT
GGCAGGCCGTACCGCCTAATAAAAGCTT

9. ACO-05

GGATCCCATGGTGACCCGCTTCCCGGATCAGCCGGGCTTCACCGGCTTCGATGCACCGATGCGTGTTGAAGCAGATCTGATGG
ATGTTGAAGTGAGTCAGGGTGCAATTCCGGAAAATCTGGAAGGTACCTATTATCGTGTTGTTGCCGACTTCCAGTGGCCGCCG
AAAGTTGATGGCGACTTCTTCTTCAATGGCGATGGTATGGTGATGAGCTTCCGCTTCAGCGGCGGTCATGCCGACTTCAAAAG
CCGCTATGTGCGCACCCCGCGCTTCGTGGCACAGGCTAAAGAACGTCGTGCCCTGTTCGGTGCATATCGTAATCCGTATACCG
ATGATCCGAGCGTTGCAGGCATGAGTCGCGGCCTGGCCAATACCAATGTGTTCTGGCATGGCGGCAAACTGCTGAGCAGTAAA
GAAGATAGCCCGCCGATGATGATTGATCCGGATACCCTGGAAACCATTGGTGAACATACCTTCGATGGCGCCCTGACCAGTCA
GACCAGTACCGCCCATCCGAAAATTGATCCGCGTACCGGTGAAATGGTGTTCTTCGGCTTCGCAGCCAAAGGTGAAACCACCC
CGGATATTGCATATTATGAAGCAGATAGCCGTGGTGTGGTGATTCATGAAACCTGGCTGCGTGCACCGTATAGCAGCATGGTT
CATGACTTCGCCGTTACCCAGAACTTCGTGGTGTTCCCGATTATTCCGCTGACCAGTGATCTGGAACGCCTGAAAGCAGGCGG
TCCGCATTATGCATGGGACCCTCATAAAGATGTGTATCTGGGTGTTCTGCCGCGTAAAGGTAATGGTGCAGATATTCGCTGGT
ATCGCGGTAGCAATCGCTTCGCAAGCCATATTATGGGCGCACATGATGATGGCCGCCATATCTTCATTGATACCCCGGTTAGT
GAAAGTAACTTCTTCCCGTTCTTCCCGGATCTGAGCGGTGCAGGTTATGAAGCAGAAAAAGCCAAAGGCTATCTGAGCCGTTG
GACCATTGATACCATGGGTGAAAGTAATGGCTTCACCGAAACCAAAATTGCCCGTTATCCGGGTGAATTCCCGCGTATGGATG
ATCGTCGCGAAACCTTACCGTATAATTGGGGTGTGCTGGCCCTGAGCGATGTTCCGGAACAGGAAAGTCCGGGTCGCGGCTTC
CGCTGGATTAGTAGTATTGATCTGAATACCGGTCATAGTCAGATCTATTATCCGGGCCATGATTGTAGCGTTGCAGAACCGCT
GTTCGTTCCGGCACATGATAATGCAGAAGAACGCGAAGGCTATGTGTTCGTTGTTATTGGCCGTCATGAAGAAATGCGCAGTG
ATCTGGTGATTCTGGATGCAGCCCATCTGGATGCACCGCCGGTGGCAACCCTGAAAATGCCGCTGCGTATTCGTAGTGGTCTG
CATGGTAATTGGGTTAGTGCCACCGAACTGGCACGTGAACGTACCGCCTAATAAAAGCTT

10. ACO-06

GGATCCCATGAACATCCGCTTCCCGAATAATGACTTCACCAGCTTCGAAGCCCCGATGCGCGCCGAAATTGATCTGAAAGGCC
TGGAAGTTATTCAGGGCGAAGTTCCGCGTCAGCTGAGCGGCGGCTTCTTCCGCCTGATTGGTGATCGCAAATGGCCGGCATTC
GTTGAAAATGATATCTTCATGCTGGGTGAAGATGGCATGGCAAGTTGCTTCTACTTCAAAGATGGTCAGGTTGACTTCAAAAT
GCGTTATGTGCGCACCCCGCGCTTCGTTGCCGAAGAAAAAGCCGGCCGTGCACTGTTCGGTCATTATCGCAATCCGTTCAGCG
ATGATCCGAGTGTTGCAGGCCTGAGTCGCGGTACCGCCAATGTGACCGTGCTGGTTCATGCCGGCAAAATGCTGGCACTGAAA
GAAGATAGCCCGGCCATTGAACTGGACCCGTTCACCCTGGAAACCATTGGCGAATATGATTGGGGTGGTGTTGTGACCGCCAA
AGCAATTACCGCACATCCGAAAATTGATCCGAAAACCGGCGAACTGGTGTTCTTCGGTTATTGTGCCGCAGGTCCGGCAACCC
GCGATATTGCATATTATGAAGCAGATGCCGAAGGTCGCATTATTCATGAAACCTGGTTCGAAGCCCCTTATACCTGTATGGTT
CATGATATTATGGTTACCGAAAATTACGTTGTGTTCCCGATTACCCCGCTGCGCCATGAACTGGAATGGATTGAACGCAATGA
ACCGGCCTTCAAATGGGACCCTAGTGAAGATGTGTATCTGGGTGTTCTGCCGCGTAAAGGTAAAGGTGAACAGGTGCGCTGGT
TCAAAGGTCCGAATCAGTGCCATGGTCATACCGTGGGTGCATTCGATGATGGCCGCTTCATCTATGCAGATAATACCTTCAGT
AAACGTAGCTTCTTCCACTTCTTCCCGAATACCGATGGCAGTGAATTCAATCCGGCAGATGCCATGCCGTTCATTAAACGCCT
GGTTATTGATATGGAAAGTAATGATGATAGCTTCACCGAACAGCAGATGTTCGGTTATCCGTGCGAACTGCCGCGTATTGATA
CCCGCTTCGAAACCATGCCGTATAACTTCGTTACCATGAATCTGCTGGATGTTCCGGAACAGGAACGCATTGGTATGGGCTTC
CAGTGGGTGGCAACCATGGATATTAATGGCCAGAAACCGGCAAAAATCTATTATGCAGGTCATGATTGTAGCATTGGTGAACC
GCAGTTCGTGCCGCGTAGCGATGATGCCGCAGAAGGTGATGGCTATATTCTGACCGTGGTTGGTAAACATCGTGAAATGCGCA
GTGAACTGCTGATTCTGGATGCAGGCGATATTACCGCACCGCCGATTGCAACCGTGGCACTGCCGTTCCGCCTGCGCAGTACC
GGTCATGGCTATTGGTATGGTACCCGTCAGCTGCGTGGCGAAACCAATATTATTGGCGCCTGGTAATAAAAGCTT

11. ACO-07

GGATCCCATGGTGTGGTTCCCGCCGCCGAAAGATCCGTTCGATGCACCGATGCGCGTTGAAGCCGATATTCGTGGTGTTCAGG
TGACCCAGGGTGAAGTTCCGGAACATCTGGATGGTACCTATTATCGTTGTGTTAGCGATCGCCAGTGGCCGAGTTATGTTGAA
AATGATCTGGAACTGTTCAATAGTGATGGTATGATTATTAGCTTCCGCTTCCATAAAGGCCGTGTTGACTTCAAAAGCCGTTA
TGTGCGCACCCCGCGCTTCGTTGCCGAAGAAAAAGCCGGTCGCGCCCTGTTCGGCGCCTATCGTAATCCGTTCGATGATGATC
CGAGTGTGGCAGGTATTAGCCGTGGCCTGGCCAATACCAATGTGTTCTTCCATGGTGGTAAACTGTATGCCAGCAAAGAAGAT
AGTCCGCCGATTCTGATTGATCCGGATACCCTGGAAACCATTGGTGAATATGACTTCGAAGGTGCACTGACCAGTCGCACCGC
AACCGCCCATCCGAAATTCGATCCGGTGACCGGCGAAATGGTGTTCTTCGGCTATGAAGCAAAAGGTCCGGGCACCCCGGATA
TTGCATATTATGAAGCAGATCCGAGTGGCCGTATTATTCATGAAGCATGGCTGGAAGCCCCGTATACCAGTATGGTTCATGAT
TGGGCCGTGACCCAGAACTTCGTTATCTTCCCGATTATTCCGCTGCGCAGCGATATTGAATGGATTAAAAAAGGTCAGAGTTA

CTTCCAGTGGGACCCTTATGAAGATGTGTATCTGGGCGTTATGCCGCGTAAAGGTACCGCCAAAGATCTGCGTTGGTATCGTG
GCAGTAATCGCTTCGCAAGTCATATTATGGGTGCATACGATGATGGCCGTTATGTGTATATTGATACCCCGGTTGGCCAGACC
AATTACTTCCCGTGGTTCCCGGATATTACCGGTGCACCGTATGATCCGGAACGCAGTAAAGGCTATCTGAGCCGCTGGACCAT
TGATACCCAGGGTGAGAGCGATGGCTTCACCGAAACCAGACTGAGCGATCTGCCGGGTGAATTCCCGCGCATGGATGATCGTC
AGGAAACCTTACCGTATCATTGGGGCATTATGCTGCTGACCGATGTGCCGGGTCGTAATGCACCGGGTGGTGGCTTCCGTTGG
CTGATGGGTATTGATCTGAAAACCGGCCGCAAACAGATCTATTATCCGGGCGATCATTGCACCCTGGGTGAAGCCATCTTCGT
TCCGGCCGCACCGGATGCCCCGCCTGGTGTGGGTTATGTTCTGACCGTTGTGGCCCGTCGTAAAGAAATGCGCAGTGATCTGG
TTCTGCTGGATGCACAGCGCCTGGATGGTGAACCGGTGTGTACCCTGAGCCTGCCGATGCGCATTCGCATGGGTGTTCATGGT
AATTGGGTTAGCGCCGCCGAACTGGCAGGTCGTACCGATTAATAAAAGCTT

12. ACO-08

GGATCCCATGACCACCCGCTTCCCGCAGGACCCTATGTTCGCAGGCTTCAGCGCACCGGTTCGTGTTGAAGCAGATATCTATG
ATCTGGAAGTTGCACAGGGTGAAGTGCCGGGTCAGCTGGATGGTACCTATTATCGTGTTGTGTGTGATCGTCAGTGGCCGCCG
ATGGTTGCAGGCGATATTCCGTTCAATGCCGATGGTATGGTTATGAGCTTCCGCTTCGAACGTGGTCATGTGGACTTCAAAAG
CCGTTATGTTCGTACCCCGCGCTTCGAAGCCGAACGTAAAGCCCGCCGTAGTCTGTTCGGCGCCTATCGCAATCCGTTCACCG
ATGATCCGAGCGTTGCCGGTATGAATCGCACCCTGGCCAATACCAATGTGTTCTGGCATGGTGGTAAACTGCTGGCCAGCAAA
GAAGATAGCCCGCCGATTCAGATTGATCCGGATACCCTGGATACCGTTGGTGTTCATACCTTCGATGGCGCCCTGACCAGCCA
GACCAGTACCGCCCATCCGAAATTCGATCCGCGTACCGGTGAAATGGTGTTCTTCGGCTTCGCAGCAAAAGGTGAATGTACCC
CGGATATTGCCTTCTATGAAGCAGATCGCGATGGCAATCTGGTTCATGAAACCTGGTTCCAGGCACCGTATAGCAGCATGGTT
CATGACTTCGCCGTTACCCAGAACTTCGTTGTGTTCCCGATTATTCCGCTGGTGAGTGATCTGGGTCGTATTCGCGCAGGTCT
GAGCCATTATGCCTGGGACCCTGGCCGTGATGTTCATCTGGGTGTGCTGCCGCGTAAAGGCCGTGTTGAAGATCTGCGTTGGT
ATCGTGGTGGCACCCGCTTCGCCAGTCATATTCTGGGTGCCTATGATGATGGCCGCCATATTCATATTGATACCCCGGTTAGT
GAAAGTAATTACTTCCCGTTCTTCCCGGATCTGAGTGGCGCACCGGCCGATCCGGGCAAAACCAAAGGCTATCTGAGCCGCTG
GACCATTGATACCATGGGCGAACGCGGCAGCTTCACCCAGGAACGCCTGACCACCCATGCAGGCGAATTCCCGCGCATGGATG
ATCGCTTCGAAACCTTACCGTATAGTTGGGGTGTGATGGGTATGTATGAAGTGCCGGGCGAACCGCGTCCGGGCAGAGGCTTC
CGTTGGGTGGGTGCCGTGGATCTGGAACATCGTCGTACCACCACCCATTATGTGGGCGATTGTAGTAGTGTGGGCGAACCGCT
GTTCGTTCCGGCCCATGAAAAAGCCGGCCATGCAGAAGGCTATGTGCTGGCAGTGGTGGGCCGCCATGATGAAATGCGTAGTG
ATCTGCTGATTCTGGATGCAGGTCGCATTGATGCCCCGCCGGTTGCAACCGTTAAACTGCCGATTCGCGTTCCGTATGGCCTG
CATGGTAATTGGGTTACCCGTGGCGAACTGCAGCGTGGCTAATAAAAGCTT

13. ACO-09

GGATCCCATGCAGCAGATTCGCTTCCCGCAGAATGAATTCCAGAGCTTCGAAGCACCGATGCGTGCCGAAGTGGATATTCGTG
GTCTGGAAGTTGTGCAGGGCGAAGTTCCGGAACATCTGGATGGCGGCTTCTATCGTCTGCTGGGTGATCGCCAGTGGCCGAGC
TTCATTGAAAATGATATCTTCCTGCTGAATGAAGATGGTATGGCAAGTTGTCTGCGTATTAGCAATGGCCGTGTTGACTTCCG
CACCCGTTATGTTAAAACACCTCGCTTCGTTGCCGAAGAACAGGCCGGCCGCGCACTGTTCGGTGCATATCGCAATCCGCTGA
CCGATGATCCGAGTGTTCAGGGTGTTCGTCGTGGCACCGCAAATGTTACCATGCTGAGTCATGCCGGCAAATTCTTCGCACTG
AAAGAAGATAGCCCGCCGGTTGAAATGGACCCGTTCACCCTGGAAACCATTGGCGAATATACCTTCGGCGGCGACTTCACCAG
CGAAGCATTCACCGCACATCCGAAAATTGATCCGCGCACCGGTGAACTGGTGTTCTATGGCTATTGTGCCAAAGGTCCGGCCA
CCAAAGATATTGCCTATTATGAAGCAGATCAGACCGGTAAAATTATTCATGAAACCTGGTTCGAAACACCTTATACCTGTATG
GTGCATGATATTATGGTTACCGAAAATTATGTGGTGTTCCCGATTACCCCGCTGCGTCATGAATATGAATGGCTGGAAAAACG
CGAACCGGCCTTCAAATATGATACCAGCGAACAGGTGCATCTGGGCGTTCTGCCGCGTAAAGGTACCGCAGATCAGATTCGTT
GGTTCGTTGGTCCGAATCAGTGTCATGGTCATACCGTTGGTGCCTATGATGATGGCCGTTATATCTATGCCGATAATACCCTG
AGTGTGCGTAGCTTCTTCCCGTTCTTCCCGAATGCCGATGGTAGTCCGTTCGATCCGGAAGATACCAAACCGTATATGAAACG
CTGGGTTATTGATATGGGCAGTGAAGGTGATACCTTCACCGAACGTCAGCTGCTGCAGTATCCGTGTGAACTGCCGCGCATTG
ATACCCGCTTCGAAACACAACCGTATAGCTTCTGCGCACTGAATCTGCTGGATGTTCCGGGCCAGGATCGTGTTGGTGGTGGC
TGGCAGTGGATTGCCACCATGGATGTGACCGGCAAAAAACCGACCCGTATTCGTTATGCAGGTGATAATTGCAGTATTAGTGA
ACCGCAGTTCGTTCCGGCCCATGATGGTGCCGCCGAAGGTGAAGGTTATGTGCTGGTTGTGGTGGGCCGTCATGATGAAATGC
GCAGTGAATTCCTGATTCTGGATGCCCAGAATATTGATGGTCCGCCGATTGCAACCGTGGCCCTGCCGATGCGTGTGCGCAGC
ACCGGTCATGGCTTCTGGTATGGCAAACGTCAGCTGATGGGTGAAACACATCTGCTGGGCGCAAATTAATAAAAGCTT

14. ACO-10

GGATCCCATGCGCACCCAGGCCGTGGCAACCACCGAACTGGATCGCCAGGTGCGCACCGAAGGCGAAACCAGACATGATAGTC
TGGATGGTGAACACATGACCCATCAGAATAATCCGGAAGAAGATGCACGTGCAGAAGAACTGGCCTGGGTTCGTACCCAGCCG
AAAGTGCCGATTGTGACCCCGAGTCCGTATCCGATGATTCGCTTCGCAGAACCGGGTCATCCGTTCGATCGCCCGGTGCGTGT
GGAAGCCGATATTGAAGGCCTGGAATGTGTTCAGGGTGAAATTCCGGCACATCTGAATGGTACCTATTATAAAGTGGTGAGTG
ATCGTCAGTTCCCGAGCTTCGTGGAAAATGATCTGGGTTACTTCAATGATGATGGCATGGCACTGAGCTTCCGCTTCCATGAT
GGCAAAGTTGATTATAAAAGTCGCTTCATTCGCACCCCGCGCTTCGAAGCCGAAAAAGCAGCAGGTCGCAGCCTGTTCGGTAA
ATATCGTAATCAGTTCGATGATGATGCCATGGTTGAAGGTATGCTGCGCGGTAATGCCAATACCAATCTGTTCTTCCATGGTG
GTAAACTGTATGCCGCAAAAGAAGATAGTCCGCCGATTATTCTGGACCCTATTACCCTGGAAACCTTAGGCGTGTATGACTTC
GAAGGCAGCCTGACCAGCAAAAGTAGCACCGCACATCCGAAAATTGATCCGATTACCGGTGAAATGGTGTTCTTCGGTTATGC
CGCAAAGGGCGATACCACCCGTGATATTGCCTATTATGAAGCAGATAGCACCGGTCAGATTATTCATGAAACCTGGTTCAAAG

CCCCGTATAGCGCCATGGTTCATGACTTCGCCGTGACCCAGAATTATGTGGTGTTCCCGATTATTCCGCTGCGTAGTGAACGT
GAATGGATTGAAAAAGGCGAAAGCAGCTTCCAGTGGGATGCCAGTGAAGATGTGTATCTGGGTGTTATGCCGCGCAAAGGCGA
TGGCGCCAAAATTCGTTGGTTCCGTGGCCGTAGTCGCTTCGCAAGCCATACCCTGGGTGCCTATGATGATGGCCGCTATATCT
ATATTGATAGTCCGGTTAGTGAAAGCAAATACTTCCCGTGGTTCCCGAATCGCGATGGCAGTCCGCATGATGCCGTTAAAGGT
CGTACCACCCTGACCCGTTGGACCATTGATCTGCATGATGGCAGCGATGGCTTCACCGAAAGCAGCATTAGTGATCTGGGCGT
TGAATTCCCGCGTATGGATGATCGCTTCGAAACCTTACAGCATACCTGGGGCGCAATGGCCGTTAGTAGTGTTCCGGGTATTG
AAATGCAGGGCCATGGCTTCCGTTGGGTGAGCACCGTGGATTATGCAACCGGCGCACAGAAACTGTATTATCCGGGTGAAGGC
AGCACCGTGGGTGAACCGGTGTTCGTTCCGGGTGCACCGGGTGCCCCGGAAGGTCATGGTTATGTGCTGGTTCTGGTTAGTCG
TCGTGATGCCATGCATAGTGATCTGGTGGTGCTGGATGCACAGAATGTGGATAAAGAACCGGTGTGTACCCTGCGTATGCCGC
TGCGCGTGCCGAAAGGTCTGCATGGCAATTGGGTTAGTGATGAAGAACTGGGCCAGCGTACCGATTAATAAAAGCTT

15. ACO-11

GGATCCCATGACCGTTCGCTTCCCGGATGAACCGGGCTTCCAGGGTCGCTTCGCACCGGTGCGTCTGGAAGGCGAAATTCGTG
GCGTGGAAGTTACCCAGGGCGAAATTCCGGCAAGCCTGAGTGGTACCTTCTATCGTGGCGGTGCAGATCCGGCCTGGCCGCCG
TTCGTTGAACGTGACTTCTACTTCAATGCAGATGGTATGGTTGCAATGTTCCGCTTCGCAAATGGCTATTGCGACTTCCGTAG
TCGTTATGTTCGTACCCCGCGCTTCGTGGCAGAACGCGCAGCCCGTCGCAGCCTGTTCGGTGCTTATCGCAATCCGTTCACCG
ATGATCCGAGTGTTGCCGGTCTGAGCCGTGGTCTGGCCAATACCAATGTGTATTGGCATGGCGGTCGTCTGCTGGCAAGCAAA
GAAGATAGTGCACCGGTTCAGATTGATCCGGATACCCTGGATACCGTTGGTGAATTCACCTGGGAAGGTGATCTGAGCAGTCA
GACCGCAACCGCACATCCGAAAATTGATCCGCGCGATGGTAGCCTGGTGTTCTTCGGCTATATGGCAAAAGGCGAAGCCACCC
GTGATATTGCATATTATGAAGCAGATGCAGCCGGTCGCATTGTTCATGAAGCCTGGTTCGAAGCCCCGTATAGTAGCATGGTT
CATGATTGGGCCGTTACCGAAAACTTCGTTATCTTCCCGATTATTCCGCTGACCGCAAGTCTGGAACGTCTGGCCGCAGGTGG
CCCGCTGTATGTGTGGGATGGTAGCGAAGATGTGTATCTGGGTGTTGTTCCGCGCCGTGGCAATAGTGTGCGCTGGTATCGTG
GCAGTAATCGCTTCGCAAGCCATATTATGAATGCATACGATGATGGTCGTCATATTCATATTGATACCCCGGTTGGTGAAAAA
AGTGCATTCCCGTGGTTCCCGGATATTGCAGGCGCCCCGTTCGATCCGGAAAAAGCAAGCGCCCAGCTGACCCGCTGGACCAT
TGATACCGCAGCACCGGAAGATCTGGGTGATGGCGCCACCGCCTTCGAACAGCGTCGCCTGACCGGCTGCAGTGGCGAATTCC
CGCGCACCGATGATCGCTGGGCCACCCGTGGTTATCGCTTCGGTGTGATTAATCTGACCGATGTGCCGGGTGAACGTCCGGAT
GATGGTCTGCCGGGCTTCCGTTGGCTGGGTCAGATTGATCCTGCAACCGGTGCCATGAAAACCAGATTCGCAGGCCGCAATAG
CACCGTGCAGGAAGCCATCTTCGTGCCGCCGGGTCGTGATACCGCAAATGGCGAAGGTTATGTTATGCAGCTGGTGGATCGCC
ATGAAACCGGCACCACCGATCTGCTGATTCTGGATGCACAGCGCATTGATGAACCGCCGGTGGCCACCCTGCGCATTCCGATT
CGTATGCCGGGTGGTCTGCATGGTAATTGGGTGACCGATGAACAGCTGGCACAGCGTGCAGGCTAATAAAAGCTT

16. ACO-12

GGATCCCATGGATCGCTTCCCGATTCCGGAAGATCCGTTCGATGCACCGATGCGTCTGGAAGCCGATATTACCGATCTGGAAT
GCGTTCAGGGCGAAGTTCCGGCACATCTGGATGGCACCTATTATCGCGTTGTTGTGGATCGTCATTATCCGAGCTTCGTGGAT
AATGATCTGGCACTGTTCAATAGCGATGGCATGGCAATGAGCTTCACCTTCCATAAAGGCCGTGTGGACTTCAAAAGCCGTTA
TGTGCGTACCCCGCGCTTCGTTGCAGAAGAAAAAGCAGGTAAAAGCCTGTTCGGTGCATATCATAATGCACTGACCGATGATC
CGAGCGTGGCCGGTGTTCGCCGTGGTCTGGCCAATACCAATATCTTCTTCCATGGTGGCCGCCTGTATGCCAGTAAAGAAGAT
AGTCCGCCGATTCTGCTGGACCCTGTTACCCTGGAAACCTTAGGCGAATATGACTTCCAGGGCGCACTGACCAGCGAAACCAG
CACCGCACATCCGAAAATTGATCCGCGTACCGGTGAAATGGTGTTCTTCGGCTATGCCGCCAAAGGCGAAGCCACCCCGGATA
TTGCCTATTATGAAGCAGATCCGAATGGTCAGATTATTCATGAAACCTGGATTCGCGCACCGTATAGTTGTATGATTCATGAC
TTCGCCGTGACCCAGAATTATGTGGTGTTCCCGATTGTGCCGCTGCGCAGCAGTATTGAATGGCTGGAAAAAGGCAATCCGCA
GTTCTTCTGGGACCCTAATGAAGATGTGCATCTGGGCGTTCTGCCGCGTAAAGGTACCGATAAAGATCTGCGCTGGTTCCGTG
GTAGTAATCGCTTCGCAAGTCATATTATGGGTGCATACGATGATGGTCGCTATCTGCATCTGGATAGTCCGGTTGCAGAAACC
AATTACTTCCCGTGGTTCCCGGATCTGAGTGGCGCCCCGTATAATCCGGATCGTAGTAAAACCAGACTGACCCGCTGGACCAT
TGATCTGAGCGGTGAAAGCGAAACCTTCACCGAACGCCGCCTGATGGATGCCGCCGGCGAATTCCCGCGCATGGATGATCGTC
AGGAAACCTTAGATTATAATTGGGGTGTTATGGCAATTAATGATCATCCGGAACAGCATAAAACCGGCGGCGGCTTCCGTTGG
GTGGGTGCAGTTGATCTGAGCAGCGGCCGCACCAAATATTATGATCCGGGTGAAAATAGTACCGTGGGCGAACCGATCTTCGT
TCCGGGCAATGATACCGCAGGTCCGGGCGCAGGTTATGTGTTCGTTGTTGTTGCCCGTCGCGATCACATGCATAGTGAACTGG
TGATTCTGGATGCACAGCATATTGATCGCCCGCCGGTGGCAACCCTGAAACTGCCGATGCGTCTTAAAAGCGGCCTGCATGGT
AATTGGGTTGGCGCAGATGAAATTGCCGGTCGTACCGCCTAATAAAAGCTT

17. ACO-13

GGATCCCATGGTTCGCTTCCCGCCGCCGAAAGATCCGTTCGATGCACCGATGCGTGTGGAAGCAGATATTCGCGGTGTGCAGG
TTACCCAGGGCGAAGTTCCGGAACATCTGGATGGTATCTATTATCGTTGCGTTAGTGATCGTCAGTGGCCGAGCTATGTGGAA
AATGATCTGGAACTGTTCAATAGCGATGGTATGATTATTAGCTTCCGCTTCCATAAAGGCCGTGTGGACTTCAAAAGTCGTTA
TGTGCGTACCCCGCGCTTCGTGGCCGAAGAAAAAGCCGGTCGTGCACTGTTCGGTGCCTATCGTAATCCGTTCGATGATGATC
CGAGCGTGGCAGGCATTAGTCGCGGCCTGGCAAATACCAATGTGTTCTATCATGGTGGCAAACTGTATGCAAGTAAAGAAGAT
AGTCCGCCGATTCTGCTGGACCCTATTACCCTGGAAACCATTGGCGAATATGACTTCGAAGGTGCACTGACCAGTCGCACCAG
TACCGCCCATCCGAAATTCGATCCGATTACCGGTGAAATGGTGTTCTTCGGCTATGAAGCAAAAGGCCCGGGTACCCCGGATA
TTGCCTATTATGAAGCAGATGCCAGCGGTCGTATTATTCATGAAGCCTGGCTGGAAGCCCCGTATACCAGTATGGTTCATGAT
TGGGCAGTGACCCAGAACTTCGTTGTGTTCCCGATTATTCCGCTGCGTAGTGATATTGAATGGATTAAACAGGGTCGTAGTTA

CTTCCAGTGGGACCCTTATGAAGATGTGTATCTGGGCGTGATGCCGCGCAAAGGTACCGCAAAAGATCTGCGCTGGTATCGTG
GTAGTAATCGCTTCGCAAGCCATATTATGGGCGCATACGATGATGGTCGCTATATCTATATTGATACCCCGGTGGGCCAGACC
AATTACTTCCCGTGGTTCCCGGATATTACCGGCGCCCCGTATGATCCGGAACGTAGCAAAGGTTATCTGAGCCGTTGGACCAT
TGATACCCAGGGTGAAAGCGATGGCTTCACCGAAACCAGACTGTGTGATCTGCCGGGCGAATTCCCGCGTATGGATGATCGCC
AGGAAACCTTACCGTATCATTGGGGTATTATGCTGCTGACCGATGGCCCGGGTCGTGTGGCACCGGGTGGTGGCTTCCGCTGG
CTGATGGGCATTGATCTGAAAACCGGTCGTAAACAGATCTATTATCCGGGTGATAATTGCACCCTGGGTGAAGCAATCTTCGT
TCCGGCCGCCCCGGATGCCCCGCCTGGTGTGGGTTATGTGTTCACCGTTGTTGCACGCCGCGCCGAAATGCGTAGTGATCTGG
TGATTCTGGATGCACAGCATATTGATGGTGAACCGGTGTGTACCCTGAGCCTGCCGATGCGCATTCGTATGGGCGTGCATGGT
AATTGGGTTAGTGCCGAAGAACTGGCACGTCGCACCGCATAATAAAAGCTT

18. ACO-14

GGATCCCATGGGTATTCGCTTCCCGAATAATGACTTCAAAAACTTCGAAGCACCGATGCGCGCAGAAATTGATCTGGCCGGCC
TGGAAGTTGTTCAGGGCGAAGTTCCGAAAGAACTGAGCGGTGGCTTCTTCCGTCTGATTGGTGATCGCAAATGGCCGGCCTTC
GTTGAAAATGATATCTTCATGCTGGGTGAAGATGGTATGGCCAGCTGCTTCTACTTCAAAGATGGCCGCGTGGACTTCAAAAT
GCGCTATGTTCGCACCCCGCGCTTCGTTGCCGAAGAAAAAGCCGGTCGCGCCCTGTTCGGCGAATATCGCAATCCGTTCACCG
ATGATCCGAGTGTGGCTGGCCTGAGTCGTGGTACCGCCAATGTTACCATGCTGGCACATGCAGGCAAACTGTTCGCAATGAAA
GAAGATAGTCCGGTTATTGAAATGGACCCGTTCACCCTGGAAACCATTGGCGAATATGATTGGGGTGGTGCCGTGACCGCAAA
AGCCATTACCGCACATCCGAAAATTGATCCGCGTACCGGCGAACTGATCTTCTTCGGCTATTGCGCCGCCGGCCCGGCAACCA
GAGATGTTGCATATTATGAAGCCGATGCCGAAGGTCGTATTATTCATGAAGCCTGGTTCAAAGCCCCGTATACCTGCATGGTG
CATGATATTATGGTGACCGAACATTATGTTGTGTTCCCGATTACCCCGCTGCGTCATGAACTGGAATGGATTGAACGCCGTGA
ACCGGCATTCAAATGGGACCCTAATGAAGAAGTGTATCTGGGTGTGCTGCCGCGCAAAGGCAATGGTGATCAGATTCGTTGGT
TCAAAGGTCCGAATCAGTGTCATGGTCATACCGTGGGTGCCTTCGATGATGGCCGCTATATCTATGCAGATAATACCTTCAGT
AAGCGTAGCTTCTTCCACTTCTTCCCGAATGTGGATGGTAGTGCATTCAGTCCGGCCGATACCATGCCGTATATGAAACGTAT
TGTTATTGATATGGAGAGCGAAGGTGATCACTTCACCGAACAGAAACTGTTCGAATATCCGTGTGAACTGCCGCGTATTGATA
CCCGCTTCGAAACCATGGATTATAACTTCGTGACCATGAATCTGCTGGATGTTCCGGGTCAGGAACGCGTTGGCCATGGCTTC
CAGTGGGTTGCAACCATGGATGTGAATGGCAATAAACCGAGCAAAATCTATTATGCAGGTGATGATTGTAGCATTGGTGAACC
GCAGTTCGTGCCGAAAAGCAATGATGCCCCGGAAGGTGAAGGCTATATTCTGACCGTTGTTGGTAAACATCAGGAAATGCGTA
GTGAACTGCTGATTCTGGATGCCAGCGATATTAATGCACCGCCGGTTGCCACCGTTGCCCTGCCGTTCCGTCTGCGTAGCACC
GGTCATGGTTATTGGTATAGCACCAGTCAGCTGCTGGGTGAAACCAATGTTCTGGGTGCCAGTTAATAAAAGCTT

19. ACO-15

GGATCCCATGAACATCCGCTTCAGCAATAATGACTTCAAATCATTCGAAGCACCGATGCGCGCCGAAATTGAACTGACCGGTC
TGGAAGTGGTGCAGGGCAAAGTGCCGAAAAATCTGGAAGGTGGCTTCTTCCGTCTGATTGGTGATCGTAAATGGCCGGCATTC
GTGGATAATGATGTGTTCATGCTGAATGAAGATGGTATGGCCAGTTGCTTCTACTTCAAAGATGGCAAAGTGGACTTCCGTAT
GCGCTATGTTCGTACCCCGCGCTTCGTTGCCGAAGAAAAAGCAGGTCGCGCACTGTTCGGCCATTATCGTAATCCGTTCAGTG
ATGATCCGAGTGTGGCCGGCCTGAGTCGTGGTACCGCCAATGTGACCATGCTGATGCATGGTGGTAAACTGCTGGCCCTGAAA
GAAGATAGTCCGGCCATTGAACTGAATCCGTTCACCCTGGAAACCATTGGTGAATATGATTGGAATGGTAAAGTGACCGCAAA
AGCAATTACCGCACATCCGAAAATTGATGCCAAAACCGGTGAACTGGTGTTCTTCGGCTATTGTGCCGCCGGCCCGGCAACCC
GTGATATTGCATATTATGAAGCCGATAGTGAAGGTAATGTGACCCATGAAACCTGGTTCCATGCACCGTATACCTGCATGGTT
CATGATATTATGGTGACCCAGAATTATGTTGTGTTCCCGATTACCCCGCTGCGCCATGAACTGGAATGGATTAAACGCAGCGA
ACCGGCATTCAAATGGGACCCTCATGAAAGTGTGTATCTGGGTGTGCTGCCGCGTAAAGGCCGCGGTGATCAGATTCGTTGGT
TCAAAGGTCCGAATCAGTGCCATGGTCATACCGTTGGTGCATACGATGATGGTCGTTATATCTATGCAGATAATACCTTCAGT
AAGCGTAGCTTCTTCCACTTCTTCCCGAATGTGGATGGTAGCCAGTTCAAACCGACCGATGCCATGCCGTATATGAAACGTGT
GGTTATTGATATGGAACGCAGTGATAATAGCTTCGTTGAACAGCCGCTGTTCGAATATCCGTGTGAACTGCCGCGTATTGATA
CCCGCTTCGAAACCTTACCGTATAGCTTCGCCACCATGAATCTGCTGGATGTGCCGGGCCAGGAACGCGTGGGCCGTGGCTTC
CAGTGGATTGCAACCATGGATGTTAATGGTGCAAAACCGAGCAAAATTCTGTATGCCGGCGATAATTGTAGTATTGGCGAACC
GCAGTTCGTTCCGGCACATGATAATGCAGCAGAAGGTGATGGCTATATTATGACCGTGGTGGGCCGCCATGAACAGATGCGCA
GCGAACTGCTGATTCTGGATGCCAGTGATATTAATGCACCGCCGGTGGCCACCGTTGCCCTGCCGTTCCGTCTGCGTAGCACC
GGCCATGGCTATTGGTATGGTGGTCGCCAGCTGCGTGGTGAAACCAACTTCCCGGGTGCATGGTAATAAAAGCTT

20. ACO-16

GGATCCCATGGCACATATCTTCGATATTGCCCCGGCCGTTAAAGGCTATGCCAGTGGTCGCCTGGCCAAACCGGGCGATGCAA
CCACCTTCCCGGATACCGATGTGTTCCGTGGTATTAATAAACCGAGTCGCTTCGAAGGTGATATCTTCGATCTGGAAGTTACC
GGCACCATTCCGAAAGATATTGATGGTACCTTCTATCGTGTTCAGCCGGATCATCGCTTCCCGCCGCTGTTCGAAGATGATAT
TCACTTCAATGGCGATGGTAGTGTGACCGCAATTCGTATTAGCAATGGCCATGCAGACTTCAAACAGCGTTATGTTCAGACCG
ATCGCTATAAAGCAGAAACCGCCGCCCGTCGCAGTCTGTTCGGCCGTTATCGTAATCCGTGGACCGATAATGAAAGTGTGAAA
GGCGTGATTCGCACCGCAAGCAATACCAATGTTACCTTCTGGCGCGGCATTCTGCTGGCAAGCAAAGAAGATGGCCCGCCGTT
CGCCATGGACCCTGTTACCCTGGAAACCTTAGGTCGCTATGACTTCGAAGGTCAGATTAAAGCCCCGACCTTCACCGCCCATC
CGAAATTCGATCCGGATACCGGCGAAATGCTGTGCTTCGCCTATGAAGCAGGCGGTGATGGCAATGATGGCAGCTGTGATGTG
GTGGTGTGGACCGTGGATGCCGATGGTAAAAAAACCGAAGAATGCTGGTATAAAGCACCGTTCGCCGGTATGATTCATGATAT
GGCCGTGAGCAAAAACTTCGTGGTGCTGCCGCTGACCCCGCTGAAAGTGAATATGGATCGTATGAAAAAAGGTGGTGAAAAAT

TCGCCTGGGACCCTAAAGAAGATCAGTATTATGGCCTGGTGCCGCGCCGCAATGCCAAACCGGAAGATATTCGCTGGTTCCGC
GCCGATAATGGCATGGCCCCGTTCCTGAAAACCTTAAGCACCCATTGTGCCGATACCCCGGCAAGCAGCGCATTCCAGGGTCA
TATTGCAGGTTGTTATGAAAATGAAGAAGGCCATGTTGTTGTGGATCTGACCGTTGCAGATGGTAATGTGTTCTTCTGGTGGC
CGCCGGATGATGGTACCGCCATTCCGCCGGGCAAAAAACCGAATCAGCTGAGCAGCACCACCACCCGCTGGATTCTGGACCCT
AAAGCAGAAACAAATACCCGTATTACCCCGGCATACGTGTGGCCGACCAATGGCGAATTCAGCCGCATTGATGATCGCTTCGT
GACCAAAAAATATAAACACTTCTGGCAGGCCAAAGTTGATCCGACCCGTCCGTATGACTTCGCAAAATGTGGTCCGCCGGCAG
GCGGCCTGTTCAATTGCCTGGGCCATTATACCTGGAGTCCGGATAATCCGCTGGCCAAAGGTGAAGAAGATGTGTTCTTCTTC
GGCCCGACCGCCACCGTGCAGGAACCGAGCTTCATTCCGAAAGACGAAGGCGGTGAAGGCGATGGCTATCTGATTGCACTGGT
TAATCATCTGGATGTTCTGCGCAATGATATTGCAGTGTTCGATGCACAGCATCTGGCCGGCGGCCCGCTGGCAGTTCTGCATC
TGCCGCTGAAACTGAAACTGGGTCTGCATGGTAACTTCGTGGATCATCGCGATATTGAAGCATGGCAGCGCCGTCGCGATGCC
AAAGGTGATGTGGGCCCGGTTAAAGTTGCAACCGAACCGCTGCCGTGGCAGAAATCATTCTGGGATCGCCGCCAGGATGGCCA
GAATGGTGGCCTGAATGGTACCCATTAATAAAAGCTT

21. ACO-16

GGATCCCATGGCCCATATCTTCGATCTGGCCCCGGAAGTTAGTCTGCCGGATGAACCGATCTATAAAGATGGCAAACTGCATC
GTCCGAATCATGTGCAGTTCCCGCAGACCCCGGTGTTCGCAAGTATGAATAAACCGAGCCGCTTCGAAGGTACCATTCTGAGC
CTGGAACATACCGGTATTATTCCGCCGGAAATTAATGGTACCTTCTTCCGCGTTCAGCCGGATCATCGCTTCCCGCCGATGTT
CGAAGATGATATTCACTTCAATGGCGATGGCAGCGTTACCGCCATTCGTATCTTCGATGGCAAAGTTGACTTCCGCCAGCGTT
ATGTTCATACCGAACGTTATAAAGCCGAAACCAAAGCACGCCGTAGCCTGTTCGGTCGCTATCGTAATCCGTGGACCGATAAT
GAAAGCGTTAAAGGCGTTATTCGTACCGCCAGTAATACCAATGTGTTCTTCTGGCGCGGTATGCTGCTGGCCACCAAAGAAGA
TGGCCCGCCGTATGCCATGGACCCTGTGACCCTGGAAACCTATGGTCGTTATGACTTCGAAGGTCAGATTCTGAGTCCGACCT
TCACCGCCCATCCGAAATTCGATCCGCGTACCGGCGAAATGGTGTGCTTCGCATACGAAACCGGTGGCGATGGTGCCGATTGT
AGCCGCGAAGTTATGGTGTGGACCCTGGATAAAGATGGTAAAAAAGTTAGTGAACGTTGGTTCGAAGCACCGTTCGCCGGCAT
GATTCATGATTGCGGTCTGAGCGAAAATTGGCTGGTGCTGCCGCTGACCCCGATTAAAATGGATCTGGAACGCATGAAACGCG
GTGGCAATAAATTCGCCTGGGACCCTAAAGAAGATCAGGTGTATGGTCTGGTGCCGCGCCCGCGGCGATGGCGAGGTGAAATGG
TTCCGTGGTGAAAATGCATTCCATGGCCATGTGGCCGGTTGCTATGAAAATGCACAGGGTCATGTTGTGATTGATCTGACCGT
TGCCGATGGTAATGTGTTCTTCTGGTTCCCGCCGGATGGTGAAGAACAGGGTCAGTTCGCAAAACGTAATAAACTGAGTAGTC
CGACCCATCGTTGGATTCTGGACCCTAGTCTGCCGAATAATGCCCGTATTACCCCGGCACTGGTGTGGCCGACCAATGGTGAA
TTCAGTCGTATTGATGATCGTTGGACCACCCGTAAATATAAACACTTCTGGCTGGCCAAAGTGGACCCTAGCCGTCCGTATGA
CTTCGCAAAATGCGGCCCGCCGGCAGGTGGTCTGTTCAATTGTCTGGGCCATTATACCTGGGATCTGGATAATGAACTGGCCA
CCGGTCAGGAAGATGTGTACTTCGCAGGCCCGACCTGTACCTTCCAGGAACCGACCTTCATTCCGAAAGGTGATAAAGAAGGT
GAAGGCTGGCTGATTGCACTGGTTAATCATCTGGATGTGCTGCGTAATGATGTTGTGATTCTGGATGCACAGAATCTGGCAAA
AGGCCCGGTGTGTACCATTCATCTGCCGCTGAAACTGAAACTGGGCCTGCATGGCAATTGGGTGGATTGGCGTGATATTGAAG
ATTGGACCAAACGCCGCCAGGAAGATGGCGAAGTGGGTCCGGTTCAGGTTGCCACCGAAATGCTGCCGTGGCAGAAAGCCTTC
TGGGAAAAAGAAAAAGAAAAGAATGGCAATGGTGTGGAAGGTCCGAATATTAATGGTACAAATGGCGCCAATGGCACCAATGG
CGTTAATGGCAGCAGCCATTAATAAAAGCTT

22. ACO-17

GGATCCCATGGCCCATATCTTCGATCTGGCCCCGGAAGTTAGTCTGCCGGATGAACCGATCTATAAAGATGGCAAACTGCATC
GTCCGAATCATGTGCAGTTCCCGCAGACCCCGGTGTTCGCAAGTATGAATAAACCGAGCCGCTTCGAAGGTACCATTCTGAGC
CTGGAACATACCGGTATTATTCCGCCGGAAATTAATGGTACCTTCTTCCGCGTTCAGCCGGATCATCGCTTCCCGCCGATGTT
CGAAGATGATATTCACTTCAATGGCGATGGCAGCGTTACCGCCATTCGTATCTTCGATGGCAAAGTTGACTTCCGCCAGCGTT
ATGTTCATACCGAACGTTATAAAGCCGAAACCAAAGCACGCCGTAGCCTGTTCGGTCGCTATCGTAATCCGTGGACCGATAAT
GAAAGCGTTAAAGGCGTTATTCGTACCGCCAGTAATACCAATGTGTTCTTCTGGCGCGGTATGCTGCTGGCCACCAAAGAAGA
TGGCCCGCCGTATGCCATGGACCCTGTGACCCTGGAAACCTATGGTCGTTATGACTTCGAAGGTCAGATTCTGAGTCCGACCT
TCACCGCCCATCCGAAATTCGATCCGCGTACCGGCGAAATGGTGTGCTTCGCATACGAAACCGGTGGCGATGGTGCCGATTGT
AGCCGCGAAGTTATGGTGTGGACCCTGGATAAAGATGGTAAAAAAGTTAGTGAACGTTGGTTCGAAGCACCGTTCGCCGGCAT
GATTCATGATTGCGGTCTGAGCGAAAATTGGCTGGTGCTGCCGCTGACCCCGATTAAAATGGATCTGGAACGCATGAAACGCG
GTGGCAATAAATTCGCCTGGGACCCTAAAGAAGATCAGGTGTATGGTCTGGTGCCGCGCCCGCGGCGATGGCGAGGTGAAATGG
TTCCGTGGTGAAAATGCATTCCATGGCCATGTGGCCGGTTGCTATGAAAATGCACAGGGTCATGTTGTGATTGATCTGACCGT
TGCCGATGGTAATGTGTTCTTCTGGTTCCCGCCGGATGGTGAAGAACAGGGTCAGTTCGCAAAACGTAATAAACTGAGTAGTC
CGACCCATCGTTGGATTCTGGACCCTAGTCTGCCGAATAATGCCCGTATTACCCCGGCACTGGTGTGGCCGACCAATGGTGAA
TTCAGTCGTATTGATGATCGTTGGACCACCCGTAAATATAAACACTTCTGGCTGGCCAAAGTGGACCCTAGCCGTCCGTATGA
CTTCGCAAAATGCGGCCCGCCGGCAGGTGGTCTGTTCAATTGTCTGGGCCATTATACCTGGGATCTGGATAATGAACTGGCCA
CCGGTCAGGAAGATGTGTACTTCGCAGGCCCGACCTGTACCTTCCAGGAACCGACCTTCATTCCGAAAGGTGATAAAGAAGGT
GAAGGCTGGCTGATTGCACTGGTTAATCATCTGGATGTGCTGCGTAATGATGTTGTGATTCTGGATGCACAGAATCTGGCAAA
AGGCCCGGTGTGTACCATTCATCTGCCGCTGAAACTGAAACTGGGCCTGCATGGCAATTGGGTGGATTGGCGTGATATTGAAG
ATTGGACCAAACGCCGCCAGGAAGATGGCGAAGTGGGTCCGGTTCAGGTTGCCACCGAAATGCTGCCGTGGCAGAAAGCCTTC
TGGGAAAAAGAAAAAGAAAAGAATGGCAATGGTGTGGAAGGTCCGAATATTAATGGTACAAATGGCGCCAATGGCACCAATGG
CGTTAATGGCAGCAGCCATTAATAAAAGCTT

23. ACO-18

GGATCCCATGGCCCATATCTTCAGTCTGGCCCCGGCCGTTAAAGGCTATAAAGATGGCCGTCTGACCCGTCCGGGTGAAGCCA
CCACCTTCCCGCCGACCCCGGTGTTCAGTGGTATTAATAAACCGAGCCGCTTCGAAGGTGATGTGTTCGATCTGGAAGTTACC
GGCACCATTCCGAAAGAAATTAATGCCACCTTCTATCGCATTCAGCCGGATCAGCGCTTCCCGCCGCTGTTCGAAGATGATGT
TCACTTCAATGGCGATGGCAGCGTTACCGCCATTCGCATTAGCGATGGTCATGCCGACTTCAAACAGCGCTATGTTCAGACCG
ATCGTTATAAAGCAGAAACCGCCGCCCGCCGTAGCCTGTTCGGCAGATATCGCAATCCGTTCACCGATAATGAAGCAGTGAAA
GGCGTGATTCGTACCGCAAGTAATACCAATATTACCTTCTGGCGCGGCATGCTGCTGGCCAGCAAAGAAGATGGTCCGCCGTT
CGCCATGGACCCTGTGACCCTGGAAACCTTAGGCCGTTATGACTTCGAAGGTCAGATTACCAGTCCGACCTTCACCGCCCATC
CGAAATTCGATCCGGATACCGGTGAAATGATCTGCTTCGCCTATGAAGCAGGTGGTAATGGCAATGATGGCAGTCTGGATATT
ATTATGTGGACCATTGATGCAGATGGTAAAAAAACCGAAGAAGCATTCTATAAAGCCCCGTTCGCAGGTATGATTCATGATAT
TGGTGTTAGCAAAAACTACGTTGTTCTGCCGCTGACCCCGATTAAAGTTAATGTTGATCGTATGAAACGCGGTGGCGAAAAAT
TCGCCTGGGACCCTTATGAAGATCAGTGGTATGGTCTGGTGCCGCGTCGTAATGGTAAAAGTGAAGATATTATCTGGTTCCGC
GCAGATAATGCATTCCAGGGCCATATTGCAGGTTGTTATGAAAATGAAGATGGCCATGTTGTGGTGGATCTGACCGTTGCCGA
TGGCAATGTGTTCTTCTGGTGGCCGCCGGATGGTAGTTATGACTTCCCGCCGTTCACCACCAGCGAACTGACCCCGATCCAGC
CGGATAAAATTACCGCCCGCGATAAACTGAATAGTCAGACCACCCGCTGGATTCTGGACCCTAAAGCCAAAACCGAAACCAGA
ATTAAACCGGCCTATGTGTGGGATATTAATGGTGAATTCAGCCGTATTGATGATCGTTGGGTGACCAAAAAATATAAACACTT
CTGGCAGGCCCGTATTGATCCGAGCAAACCGTATGACTTCCAGAAATGCGGCCCGCCGGCCGGCGGTCTGTTCAATAGCCTGG
GCCATTATACCTGGAATCCGGATGATGCCCTGTGCCATGGCGAAGAAGATACCTACTTCTTCGGCCCGACCAGCACCGTTCAG
GAACCGAGCTTCATTCCGCGCGGCGAAGATGCCGCCGAAGGCGAAGGCTATCTGATTGCACTGGTGAATCGTCTGGATGAACT
GCGTAATGATGTTGCCGTGTTCGATGCACAGAATGTGGCACAGGGCCCGCTGGCAGTTATTCATCTGCCGCTGAAACTGAAAC
TGGGCCTGCATGGTAACTTCGTGGATCATCGCGATATTGAAGCATGGCAGCGCCGTCGCGATGCCGGTGGTGATATTGGCCCG
GTTAAAATTGCAACCGAACCGCTGCCGTGGCAGTAATAAAAGCTT

24. ACO-19

GGATCCCATGGCACATATTCATGATCTGGCACCGCAGATTAATCAGTATTATCTGGGTGGTCGCAAACAGGATAATCAGCTGA
AATTCCCGGATACCGATGTGTTCAAAGGCTTCAATGCACCGAGCCGTATTGAAGGTGAAATTATTAATCTGGAAGTGGATGGT
ACCATTCCGGCAGATATTGATGGCACCTTCTATCGCATTCAGCCGGATCATCGTTATCCGCCGATGTTCGAAGATGATATTCA
GTTCAATGGTGATGGCGCAGTTACCGCAATTCGCATTCAGAAAGGCCATGCCGACTTCAAACAGCGCTTCGTGAAAACCGATC
GCTATGAAGCAGAAACCGCAGAACGCCGTGCACTGTTCGGCCGTTATCGCAATCTGTATACCGATGATGAACTGGTGAAAGGC
GTGATTCGCACCGCAAGCAATACCAATGTGGTGTTCTGGCGTGGCATGCTGCTGGCAATGAAAGAAGATGGTCCGCCGTTCGC
AATGGACCCTGTGACCCTGGAAACCATTGGTCGTTATGACTTCGAAGGCCAGGTGCAGAGTCCGACCTTCACCGCCCATCCGA
AAATTGATCCGGTTACCGGTGAAATGGTGTGCTTCGGTTATGAAGCCGGCGAAAATGGCAATGATGCCAGTAATGATATTGTT
GTGTATACCATTAACGCAGATGGTGTTAAAACCGATGAATGCTGGTATAAAAGCCCGTTCTGCGGCATGATTCATGATTGTGG
TATTAGTGCAAATTACCTGGTGATGCCGCTGACCCCGCTGAAATGTAGCCTGGATCGCCTGAAAAAAGGCGGCAATCATTGGG
CCTGGGACCCTAATGAAGATCAGTGGTATGGCGTTGTTCCGCGTCGTGGCGGCCGTCCTAATGATATTAAATGGTTCCGTAGT
GCAAATGCATTCCATGGTCATACCGCCGGTTGCTATGAAACCGAAGATGGTCGTATTATCTATGATCTGACCGTTGCAGATGG
TAATGTGTTCTTCTTCTTCCCGCCGGATGATATGCCGCCGGGTAGCGTTCTGAAACGTAATAAACTGAAAAGCGAAACCTGTC
GTTGGATTCTGGACCCTAAAGCACCGAGCGGCACCTATGTGACCCCGGCACAGAGCTGGAATACCAGCGGTGAATTCAGTCGC
ATTGATGATCGTCTGGTTACCAAACGCTATGATCACTTCTGGCAGTGTAAAATTGATCCTACCCGCGAATATGACTTCGCAAG
TTGTGGTCCGCCGGCAGGTGGCCTGTTCAATTGCCTGGGCCATTATACCTGGAGCGGCCAGACCGAAGATCTGATTGCCCTGG
TGAATCAGCTGGATGTGCTGCGCAATGATATTATGATCTTCGATGCACTGCATCTGAGCGCAGGTCCGGTTGCCACCATTCGC
CTGCCGTTCAAACTGAAACTGGGCCTGCATGGTAACTTCGTGGATCATGCCGATATTGCCGAATGGGAAGCACTGCGCCGTCG
TGCCGGTGGTATTGGCCCGGTTAAACCGGCCGAACGTCCGCTGCCGTGGCAGTTAGCAATGAATGGTACCGCAAGTAATGGCC
AGAGCGGTCGCAATTGTGTTAATGGCCTGAGCCATTAATAAAAGCTT

25. ACO-20

GGATCCCATGACCCATCCGTTCCCGCAGACCCCGGAATTCAGTGGCGCACTGTATGCACCGAGCCGCGTTGAAGCAGAAGTGT
TCGATCTGGAAGTGGAAGGTACCCTGCCGGATAGCATTCGTGGTGCCTTCTATCAGGTTGCACCGGACCCTCAGTATCCGCCG
ATGCTGGGTAATGATATCTTCTTCAATGGTGATGGTATGGTTAGCGCCTTCAACTTCGCCGATGGCAAAGTTAGTATGCGTCG
TCGCTATGTTAAAACCGATCGCCTGATGGCACAGCGCCGTGAAGGCCGCAGTCTGAATGGTGTGTATCGCAATGTGTTCACCA
ATGATCCGCTGGCAGCAAAAAATAATACCACCGCCAATACCAGCGTGGTGGCACATAATGGTGTGCTGCTGGCACTGAAAGAA
GATGCACTGCCGTGGGCAATGGATCTGGAAACCTTAGAAACCTTAGGCGAATGGAGCTTCGATGGTCAGATTGAAAGTGCAAC
CTTCACCGCACATCCGAAACTGGACCCTGTGACCGGTAATCTGCTGGCATTCAGTTATGAAGCAAAAGGCGATGGCACCCCGG
ATCTGGCCTACTTCGAACTGAGTCCGGATGGCAAACTGCTGCATCAGATCTGGTTCCAGGCACCGTATGCAGCAATGGTGCAT
GACTTCGCAGTGACCGAACATTATGTTGTGTTCCCGCTGATTCCGCTGACCGTTGATGTTGAACGTATGAAAAATGGCGGTCA
GCACTTCCAGTGGCAGCCGGATCTGCCGCAGCTGTTCGCAGTTGTGCCGCGTAATGGCCATGCCGAAGATGTTCGCTGGTTCA
AAGGCCCGAAAGATGGCTTCCAGGGTCATACCCTGAATGCATTCGATGAAGATGATAAAGTGTATGTGGATATGCCGGTTACC
GGTGGTAATATCTTCTACTTCTTCCCGCAGGCAGATGGTTATGTTCCGCCGCCGGAAACCTTAGCAGCAAGTCTGATGCGCTG
GACCTTCGATCTGACCAGCACCCAGGATGATATTCAGCCGCAGCCGCTGACCGATTATCCGTGCGAATTCCCGCGTTGCGATG
ATCGCTATATTGGCCGTAAATATGAACATGGCTTCGTTCTGGCCTTCGATCCGGAACGCCCGTATAATCCGGCCAATGGCCCG
ATTCCGTTCCAGTTCTTCAATCTGCTGGCCCGTGTGAATCTGAAAACCGGCGCAACCGATGCATGGTTCCCGGGTGATAGCGG
TTGCTTCCAGGAACCGATCTTCATTCCGCGTGCACCGGATGCACAGGAAGCAGATGGCTATGTTGTTGCACTGCTGAATCTGA

TTGCCGAAGGTCGTAGTGAACTGGTGGTGCTGGATAGTCGCGATATGGCCAGTGGCCCGATTGCCCGTATTAAAGTTCCGTTC
CGTCTGCGTATGAGTCTGCATGGCTGCTGGGTTCCGAGTAAAAAATAATAAAAGCTT

26. ACO-21

GGATCCCATGACCATTCCGTTCCCGCAGACCCCGGAATTCAGCGGTGCCCTGTATACCCCGAGCCGTGTGGAAGCCGAAGTGT
TCGATCTGGAAATTGAAGGCGCCCTGCCGGCCAGTATTCGTGGTACCTTCTATCAGGTTGCACCGGACCCTCAGTATCCGCCG
ATGCTGGGCACCGATATCTTCTTCAATGGTGATGGTATGGTGAGCGGCTTCTACTTCGCAAATGGCAAAGTTAGCCTGCGTCG
TCGTTATGTGAAAACCGATCGTCTGCTGGCCCAGCGCCGTGAAGGCCGTAGCCTGAATGGCATCTATCGTAATGTGTATACCA
ATGATCCGCTGGCAGCAAAAAATAATACCACCGCAAATACCACCGTTGTGCCGCATAATGGTGTTCTGCTGGCCCTGAAAGAA
GATGCAATGCCGTGGGCAATGGATCTGGAAACCTTAGAAACCTTAGGCGAATGGAACTTCGATGGTCAGATTAAAAGCGCCAC
CTTCACCGCCCATCCGAAACTGGACCCTGCCACCGGTAATCTGCTGGCCTTCAGCTATGAAGCCAAAGGTGAAGGCACCCCGG
ATCTGGCATACTTCGAACTGAGTCCGGATGGTAAACTGCTGCATGAAATCTGGTTCCAGGCCCCGTATGCCGCCATGGTTCAT
GACTTCGCCGTGACCGAACATTATGTGGTGTTCCCGCTGATTCCGCTGACCGTTGATATTGAACGTATGAAAAATGGCGGTCC
GCACTTCCAGTGGCAGCCGGATCTGCCGCAGCTGTTCGCAGTGGTTCCGCGTAATGGCTGTGCCCAGGATGTTCGTTGGTTCA
AAGGTCCGAAAGAAGGCTTCCAGGGTCATACCCTGAATGCCTTCGATGAAGATGGCAAAGTGTATGTTGATATGCCGGTTACC
GGTGGTAATATCTTCTACTTCTTCCCGCAGGCAGATGGTTATGTTCCGCCGCCGGAAACCTTAGCAGCAAGCCTGATGCGTTG
GACCTTCGATCTGAATGGCAGTCAGGATGAAATTCAGCCGCAGCCGCTGACCGATTATCCGTGTGAATTCCCGCGCTGTGATG
ATCGCTATATTGGTCGTAAATATGAACATGGCTTCCTGCTGGCCTTCGATCCGCAGCGCCCGTATAATCCGGAAAATGGTCCG
ATTCCGTTCCAGTTCTTCAATCTGCTGGCACATGTTAATCTGAAAACCGGTACCACCGATGCATGGTTCCCGGGTGATAGTGG
TTGCTTCCAGGAACCGATCTTCATTCCGCGCAGCCCGAATGCCGAAGAAGCCGATGGTTATGTGGTGAGTCTGCTGAATCTGA
TTGCCGAAGGCCGTAGTGAACTGGTTGTGCTGGATAGCCGTGATATGGAAAGCGGCCCGATTGCCCGTATTAAAGTTCCGTTC
CGTATGCGTATGAGCCTGCATGGCTGTTGGGCACCGAGCTAATAAAAGCTT

27. ACO-22

GGATCCCATGACCATTCCGTTCCCGCAGACCAGCGAATTCAGTGGCGCACTGTATACCCCGAGTCGTGTGGAAGCCGAACTGT
TCGATCTGGAAGTGGAAGGCACCCTGCCGGCAGAAATTCGCGGTGCCTTCTATCAGGTGGCACCGGACCCTCAGTATCCGCCG
ATGCTGGGTACCGATATCTTCTTCAATGGTGATGGTATGGTGACCGGCTTCTACTTCGCAAATGGCAAAGTGAGCATGCGTCG
TCGCTATGTTAAAACCGATCGTCTGATGGCACAGCGTCGTGAAGGTCGCAGTCTGAATGGTGTGTATCGCAATCTGTATACCA
ATGATCCGCTGGCCGCAAAAAATAATACCACCGCCAATACCACCGTTATTCCGCATAATGGTGTTCTGCTGGCCCTGAAAGAA
GATGCCCTGCCGTGGGCAATGGATCTGGAAACCTTAGAAACCTTAGGTGAATGGACCTTCGATGGTCAGATTAAAGCAGCAAC
CTTCACCGCACATCCGAAACGCGATCCGGCAACCGGCAATCTGCTGGCATTCAGTTATGAAGCAAAAGGCGATGGCACCCCGG
ATATTGCCTACTTCGAAATTAGTCCGGATGGCAAACTGCTGCATGAAATCTGGTTCCAGGCACCGTATGCCGCCATGGTTCAT
GACTTCGCCGTGACCGAACGTTATGTGGTGTTCCCGCTGATTCCGCTGACCGTTGATGTGGAACGTATGAAAAATGGCGGCCC
GCACTTCCAGTGGCAGCCGGATCTGCCGCAGCTGTTCGCAGTGGTGCCGCGTAATGGCAATGCACAGGATGTGCGCTGGTTCA
AAGGTCCGAAAGATAGCTTCCAGGGTCATACCCTGAATGCATTCGATGAAGATGGCAAAGTGTATGTTGATATGCCGGTTACC
GGCGGTAATGTGTTCTACTTCTTCCCGCAGGCCGATGGCTATGTGCCGCCGCCTGAAACCTTAGCAGCAAGTCTGATGCGCTG
GACCTTCGACCTGAGCAGTCCGCAGGATGAAGTGGAACCGCAGCCGCTGACCGAATATCCGTGCGAATTCCCGCGTTGCGATG
ATCGCTATATTGGCCGCAAATATCAGCATGGCTTCCTGCTGGCCTTCGATCCGGAACGCCCGTATAATCCGGCCAATGGTCCG
ATTCCGTTCCAGTTCTTCAATCTGCTGGTGCATCTGAATCTGAAAACCGGCAGCACCGATGCATGGTTCCCGGGTGATAGCGG
TTGCTTCCAGGAACCGATCTTCATTCCGCGCAGTGCAGATGCAGAAGAAGCAGATGGTTATGTTGTTGCCCTGCTGAATCTGA
TTGCCGAAGGCCGTAGTGAACTGGTTGTGCTGGATAGCCGCGATATGGCCGGTGGCCCGATTGCCCGTATTCGCATTCCGTTC
CGTATGCGCATGAGTCTGCATGGTTGTTGGGCACCGAGCGATTAATAAAAGCTT

28. ACO-23

GGATCCCATGACCATTCCGTTCCCGCAGACCCCGGAATTCAGCGGTGCCCTGTATACCCCGAGCCGTGTGGAAGCAGAAGTGT
TCGATCTGGAAATTGAAGGCGTTCTGCCGGCAAGCATTGGCGGCACCTTCTATCAGGTTGCACCGGACCCTCAGTATCCGCCG
ATGCTGGGTAATGATATCTTCTTCAATGGTGATGGTGTGGTTAGTCGCTTCAACTTCGCAAATGGTAAAGTTAGTATGCGCCG
CCGCTATGTGAAAACCGATCGTCTGCTGGCACAGCGCCGTGAAGGTCGTAGCCTGAATGGCGTGTATCGCAATGTGTATACCA
ATGATCGCCTGGCAGCAAAAAATAATACCACCGCCAATACCACCGTGATTCCGCATAATGGTGTTCTGCTGGCCCTGAAAGAA
GATGCCCTGCCGTGGGCAATGGATCTGGATACCCTGGAAACCTTAGGCGAATGGACCTTCGATGGCCAGATTAAAAGCGCAAC
CTTCACCGCACATCCGAAACTGGACCCTGTGACCGGTAATCTGCTGGCATGCAGCTATGAAGCCAAAGGCGATGGTACCCCGG
ATCTGGCCTACTTCGAAATTAGCCCGGATGGCAAACTGCTGCATGAAATCTGGTTCCAGGCACCGTATGCCGCCATGGTTCAT
GACTTCGCCGTTACCGAACGTTATGTGGTGTTCCCGCTGATTCCGCTGACCGTGGATGTGGAACGTATGAAAAATGGCGGCCC
GCACTTCCAGTGGCAGCCGGATCTGCCGCAGCTGTTCGCCGTGGTGCCGCGTAATGGTAATGCCCAGGATGTTCGTTGGTTCA
AAGGTCCGAAAGATGGCTTCCAGGGTCATACCCTGAATGCCTTCGATGAAGATGGCAAAGTGTATGTGGATATGCCGGTTACC
GGCGGCAATATCTTCTACTTCTTCCCGCAGGCAGATGGTTATGTTCCGCCGCCGGAAACCTTAGCCGCAAGTCTGATGCGTTG
GACCTTCGACCTGAATAGCGCACTGGATGAAGTGGAACCGCAGCCGCTGACCGATTATCCGTGCGAATTCCCGCGCTGTGATG
ATCGTTATATTGGTCGTAAATATCAGCATGGCTTCCTGCTGGCATTCGATCCGGAACGTCCGTATAATCCGGCAAATGGCCCG
ATTCCGTTCCAGTTCTTCAATCTGCTGGTGCATCTGAATCTGAAAACCGGCATTACCGATGCCTGGTTCCCGGGCGATAGTGG
CTGCTTCCAGGAACCGATCTTCATTCCGCGTAGCGCAGATGCAGAAGAAGCAGATGGTTACGTGGTTGCCCTGCTGAATCTGA

TTGCAGAAGGTCGTTCAGAACTGGTGGTTCTGGATAGTCGCGATATGGCAAGTGGTCCGATTGCACGCATTCGCATTCCGTTC
CGCATGCGCATGAGTCTGCATGGCTGTTGGACCGCCGATCCGAATTAATAAAAGCTT

29. ACO-23

GGATCCCATGAGCATTCCGTTCCCGCAGACCCCGGAATTCAGTGGTGTGCTGTATACCCCGAGCCGTGTGGAAGCCGAAGTGT
TCGATCTGGAAATTGAAGGCATTCTGCCGGCAAGCATCTGTGGTACCTTCTATCAGGTGGCCCCGGACCCTCAGTATCCGCCG
ATGCTGGGCAATGATATCTTCTTCAATGGCGATGGTGTGGTGAGTCGCTTCAACTTCGCAAATGGTAAAGTGAGTATGCGCCG
CCGTTATGTGAAAACCGATCGCCTGCTGGCCCAGCGTCGTGAAGGTCGTAGTCTGAATGGTGTGTATCGTAATGTGTATACCA
ATGATCCGCTGGCCGCCAAAAATAATACCACCGCAAATACCACCGTGATTCCGCATAATGGTGTGCTGCTGGCACTGAAAGAA
GATGCACTGCCGTGGGCACTGGATCTGGAAACCTTAGAAACCTTAGGTGAATGGACCTTCGATGGTCAGATTAAAGCAGCCAC
CTTCACCGCCCATCCGAAACTGGACCCTGTGACCGGTAATCTGCTGGCCTGTAGTTATGAAGCCAAAGGTGATGGCACCCCGG
ATCTGGCATACTTCGAAATTAGCCCGGATGGCAAACTGCTGCATGAAATCTGGTTCCAGGCCCCGTATGCAGCCATGGTGCAT
GACTTCGCCGTTACCGAACGTTATGTGGTGTTCCCGCTGATTCCGCTGACCGTGGATGTGGAACGCATGAAAAATGGTGGTCC
GCACTTCCAGTGGCAGCCGGATCTGCCGCAGCTGTTCGCCATTGTTCCGCGCAATGGCTGCGGTCAGGATGTTCGCTGGTTCA
AAGGTCCGAAAGATGGCTTCCAGGGCCATACCCTGAATGCATTCGATGAAGATGGTAAAGTGTATGTGGATATGCCGGTGACC
GGTGGTAATATCTTCTACTTCTTCCCGCAGGCAGATGGCTATGTTCCGCCGCCGGAAACCTTAGCCGCAAGTCTGATGCGTTG
GACCTTCGACCTGAATGGCGCACAGGAAGATGTTCAGCCGCAGCCGCTGACCGAATATCCGTGCGAATTCCCGCGTTGCGATG
ATCGTTATATTGGCCGTAAATATCAGCATGGCTTCCTGCTGGCATTCGATCCGGAACGCCCGTATAATCCGGCAAATGGCCCG
ATTCCGTTCCAGTTCTTCAATCTGCTGGCACATCTGGATCTGCAGACCGGTCGTACCGATGCCTGGTTCCCGGGTGATAGTGG
CTGCTTCCAGGAACCGATCTTCATTCCGCGTAGCGCAGATGCAGAAGAAGCCGATGGTTATGTTGTGGCCCTGCTGAATCTGA
TTGCAGAAGGCCGTAGTGAACTGGTGGTGCTGGATACCCGTGATATGGCCAGTGGCCCGATTGCCCGTATTCGCATTCCGTTC
CGTATGCGTATGAGCCTGCATGGCTGCTGGGCACCGAATGATTAATAAAAGCTT

LIST OF PLASMIDS

Entry	Enzyme	pEG[a]	Name	Vector Backbone	TAG	Cloning Strategy
1	*Rj*EUGO	605	pBAD-EUGO	pBAD	HisX6 at the C-term	Restriction sites removed // Two stop codons
2	*Pn*IECO	604	pET-51b(+)_IECO	pET-51b(+)	Strep-Tag-II at the N-term	BamHI – HindIII // Additional C before ATG // Two stop codons
3	*Pb*LSD	636	pET-51b(+)_PbLSD	pET-51b(+)	Strep-Tag-II at the N-term	BamHI – HindIII // Additional C before ATG // Two stop codons
3	ACO-01 (*Tt*Ado)	637	pET-51b(+)_CO-01	pET-51b(+)	Strep-Tag-II at the N-term	BamHI – HindIII // Additional C before ATG // Two stop codons
5	ACO-02	n.a.	pET-51b(+)_CO-02	pET-51b(+)	Strep-Tag-II at the N-term	BamHI – HindIII // Additional C before ATG // Two stop codons
6	ACO-03	638	pET-51b(+)_CO-03	pET-51b(+)	Strep-Tag-II at the N-term	BamHI – HindIII // Additional C before ATG // Two stop codons
7	ACO-03 C26N	678	pET-51b(+)_CO-03 C26N	pET-51b(+)	Strep-Tag-II at the N-term	BamHI – HindIII // Additional C before ATG // Two stop codons
8	ACO-04	639	pET-51b(+)_CO-04	pET-51b(+)	Strep-Tag-II at the N-term	BamHI – HindIII // Additional C before ATG // Two stop codons
9	ACO-05	n.a.	pET-51b(+)_CO-05	pET-51b(+)	Strep-Tag-II at the N-term	BamHI – HindIII // Additional C before ATG // Two stop codons
10	ACO-06	640	pET-51b(+)_CO-06	pET-51b(+)	Strep-Tag-II at the N-term	BamHI – HindIII // Additional C before ATG // Two stop codons
11	ACO-07	641	pET-51b(+)_CO-07	pET-51b(+)	Strep-Tag-II at the N-term	BamHI – HindIII // Additional C before ATG // Two stop codons
12	ACO-08	n.a.	pET-51b(+)_CO-08	pET-51b(+)	Strep-Tag-II at the N-term	BamHI – HindIII // Additional C before ATG // Two stop codons
13	ACO-09	642	pET-51b(+)_CO-09	pET-51b(+)	Strep-Tag-II at the N-term	BamHI – HindIII // Additional C before ATG // Two stop codons
14	ACO-10	643	pET-51b(+)_CO-10	pET-51b(+)	Strep-Tag-II at the N-term	BamHI – HindIII // Additional C before ATG // Two stop codons
15	ACO-11	n.a.	pET-51b(+)_CO-11	pET-51b(+)	Strep-Tag-II at the N-term	BamHI – HindIII // Additional C before ATG // Two stop codons
16	ACO-12	n.a.	pET-51b(+)_CO-12	pET-51b(+)	Strep-Tag-II at the N-term	BamHI – HindIII // Additional C before ATG // Two stop codons
17	ACO-13	644	pET-51b(+)_CO-13	pET-51b(+)	Strep-Tag-II at the N-term	BamHI – HindIII // Additional C before ATG // Two stop codons

Entry	Enzyme	pEG[a]	Name	Vector Backbone	TAG	Cloning Strategy
18	ACO-14	645	pET-51b(+)_CO-14	pET-51b(+)	Strep-Tag-II at the N-term	BamHI – HindIII // Additional C before ATG // Two stop codons
19	ACO-15	646	pET-51b(+)_CO-15	pET-51b(+)	Strep-Tag-II at the N-term	BamHI – HindIII // Additional C before ATG // Two stop codons
20	ACO-16	647	pET-51b(+)_CO-16	pET-51b(+)	Strep-Tag-II at the N-term	BamHI – HindIII // Additional C before ATG // Two stop codons
21	ACO-17	648	pET-51b(+)_CO-17	pET-51b(+)	Strep-Tag-II at the N-term	BamHI – HindIII // Additional C before ATG // Two stop codons
22	ACO-18	n.a.	pET-51b(+)_CO-18	pET-51b(+)	Strep-Tag-II at the N-term	BamHI – HindIII // Additional C before ATG // Two stop codons
23	ACO-19	n.a.	pET-51b(+)_CO-19	pET-51b(+)	Strep-Tag-II at the N-term	BamHI – HindIII // Additional C before ATG // Two stop codons
24	ACO-20	649	pET-51b(+)_CO-20	pET-51b(+)	Strep-Tag-II at the N-term	BamHI – HindIII // Additional C before ATG // Two stop codons
25	ACO-21	650	pET-51b(+)_CO-21	pET-51b(+)	Strep-Tag-II at the N-term	BamHI – HindIII // Additional C before ATG // Two stop codons
26	ACO-22	651	pET-51b(+)_CO-22	pET-51b(+)	Strep-Tag-II at the N-term	BamHI – HindIII // Additional C before ATG // Two stop codons
27	ACO-23	652	pET-51b(+)_CO-23	pET-51b(+)	Strep-Tag-II at the N-term	BamHI – HindIII // Additional C before ATG // Two stop codons
28	ACO-24	653	pET-51b(+)_CO-24	pET-51b(+)	Strep-Tag-II at the N-term	BamHI – HindIII // Additional C before ATG // Two stop codons

[a]pEG (plasmid elk group): depository number for the internal plasmid database. Only soluble expressed proteins are included in the database.

REFERENCES

[1] M. Remmert, A. Biegert, A. Hauser, J. Soding, *Nat Methods* **2011**, *9*, 173-175.

[2] L. E. Baum, T. Petrie, *The Annals of Mathematical Statistics* **1966**, *37*, 1554-1563.

[3] S. F. Altschul, W. Gish, W. Miller, E. W. Myers, D. J. Lipman, *J. Mol. Biol.* **1990**, *215*, 403-410.

[4] L. Zhao, Y. Xie, L. Chen, X. Xu, C. X. Zhao, F. Cheng, *Process Biochem.* **2018**, *71*, 76-81.

[5] M. Yamada, Y. Okada, T. Yoshida, T. Nagasawa, *Biotechnol. Lett.* **2008**, *30*, 665-670.

[6] a) X.-Y. Lu, X.-M. Wu, B.-D. Ma, Y. Xu, *Catalysts* **2021**, *11*, 1199-1199; b) Q. Wang, X. Wu, X. Lu, Y. He, B. Ma, Y. Xu, *Appl. Biochem. Biotechnol.* **2021**, *193*, 1116-1128; c) J.-Y. Ryu, J. Seo, S. Park, J.-H. Ahn, Y. Chong, M. J. Sadowsky, H.-G. Hur, *Biosci. Biotechnol. Biochem.* **2013**, *77*, 289-294.

[7] P. C. Loewen, J. Switala, J. P. Wells, F. Huang, A. T. Zara, J. S. Allingham, M. C. Loewen, *BMC Biochem.* **2018**, *19*, 8-8.

[8] J. Ni, Y.-T. Wu, F. Tao, Y. Peng, P. Xu, *J. Am. Chem. Soc.* **2018**, *140*, 16001-16005.

[9] R. Chenna, H. Sugawara, T. Koike, R. Lopez, T. J. Gibson, D. G. Higgins, J. D. Thompson, *Nucleic Acids Res.* **2003**, *31*, 3497-3500.

[10] T. J. Wheeler, J. D. Kececioglu, *Bioinformatics* **2007**, *23*, i559-568.

[11] J. Jin, H. Mazon, R. H. H. van den Heuvel, D. B. Janssen, M. W. Fraaije, *FEBS J.* **2007**, *274*, 2311-2321.

[12] A. Okawara, K. Hada, R. Kurotani, K. Teruya, H. Konno, *Tetrahedron Lett.* **2023**, *127*, 154665.

[13] B. Kokić, Ž. Selaković, A. M. Nikolić, A. Andrijević, B. Anđelković, V. Ajdačić, I. M. Opsenica, *Eur. J. Org. Chem.* **2022**, *2022*, e202201112.
